# Supplementary material for: Virtual library docking for cannabinoid-1 receptor agonists with reduced side effects
Source: Nat Commun. 2025 Mar 6;16:2237. doi: 10.1038/s41467-025-57136-7 (PMC11882969; doi:10.1038/s41467-025-57136-7)
Supplement: Supplementary file 1 — Supplementary Information [file 41467_2025_57136_MOESM1_ESM.pdf]

# Supplementary Information for

## Virtual library docking for cannabinoid-1 receptor agonists with reduced side effects

Tia A. Tummino[1,2,†], Christos Iliopoulos-Tsoutsouvas[3,†], Joao M. Braz[4,†], Evan S. O'Brien[5], Reed M. Stein[1,2], Veronica Craik[4], Ngan K. Tran[3], Suthakar Ganapathy[3], Fangyu Liu[1], Yuki Shiimura[5,6], Fei Tong[3], Thanh C. Ho[3], Dmytro S. Radchenko[7], Yurii S. Moroz[7,8,9], Sian Rodriguez Rosado[4], Karnika Bhardwaj[4], Jorge Benitez[4], Yongfeng Liu[10], Herthana Kandasamy[11], Claire Normand[11], Meriem Semache[11], Laurent Sabbagh[11], Isabella Glenn[1], John J. Irwin[1], Kaavya Krishna Kumar[5,*], Alexandros Makriyannis[3,12*], Allan I. Basbaum[4,*], & Brian K. Shoichet[1,*]

†Contributed equally.

*Correspondence: kaavyak@stanford.edu, a.makriyannis@northeastern.edu, allan.basbaum@ucsf.edu, bshoichet@gmail.com

This file includes:

Supplementary Figures 1 to 11
Supplementary Tables 1 to 13
Supplementary Methods
Supplementary References

## Supplementary Figures

a

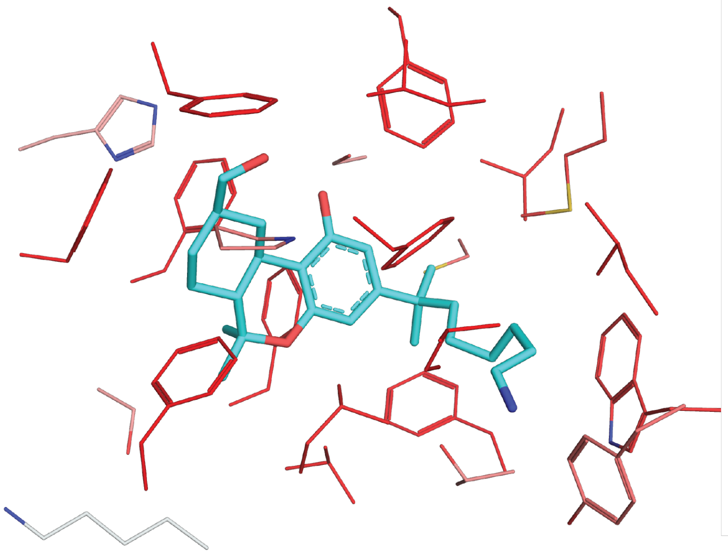

b

| Residue Number | Residue Type | Polarity | Kyte & Doolittle Scale [-4.5-4.5] | Eisenberg Scale [-2.5-1.38] |
|---|---|---|---|---|
| 108 | PHE | NP | 2.80 | 1.19 |
| 170 | PHE | NP | 2.80 | 1.19 |
| 173 | SER | P | -0.80 | -0.18 |
| 174 | PHE | NP | 2.80 | 1.19 |
| 177 | PHE | NP | 2.80 | 1.19 |
| 178 | HIS | C/P | -3.20 | -0.4 |
| 189 | PHE | NP | 2.80 | 1.19 |
| 192 | LYS | C | -3.90 | -1.5 |
| 193 | LEU | NP | 3.80 | 1.06 |
| 196 | VAL | NP | 4.20 | 1.08 |
| 197 | THR | P | -0.70 | -0.05 |
| 200 | PHE | NP | 2.80 | 1.19 |
| 267 | ILE | NP | 4.50 | 1.38 |
| 268 | PHE | NP | 2.80 | 1.19 |
| 269 | PRO | NP | -1.60 | 0.12 |
| 271 | ILE | NP | 4.50 | 1.38 |
| 275 | TYR | P | -1.30 | 0.26 |
| 276 | LEU | NP | 3.80 | 1.06 |
| 279 | TRP | NP | -0.90 | 0.81 |
| 359 | LEU | NP | 3.80 | 1.06 |
| 363 | MET | NP | 1.90 | 0.64 |
| 379 | PHE | NP | 2.80 | 1.19 |
| 383 | SER | P | -0.80 | -0.18 |
| 386 | CYS | NP | 2.50 | 0.29 |
| | | **Average** | 1.59 | 0.68 |

Supplementary Figure 1. Hydrophobicity calculations for the hCB1R orthosteric pocket based on PDB: 5XR8. Residues within 5 Å of AM841 are considered. a. Depiction of the hCB1 orthosteric pocket, colored by the Eisenberg Scale, where darker red colors indicate more hydrophobic residues, and lighter red or gray colors indicate less hydrophobic residues. b. A table of the residues within 5 Å of AM841, with their polarity class (nonpolar (NP), polar (P), or charged (C)), and two hydrophobicity scores indicated.

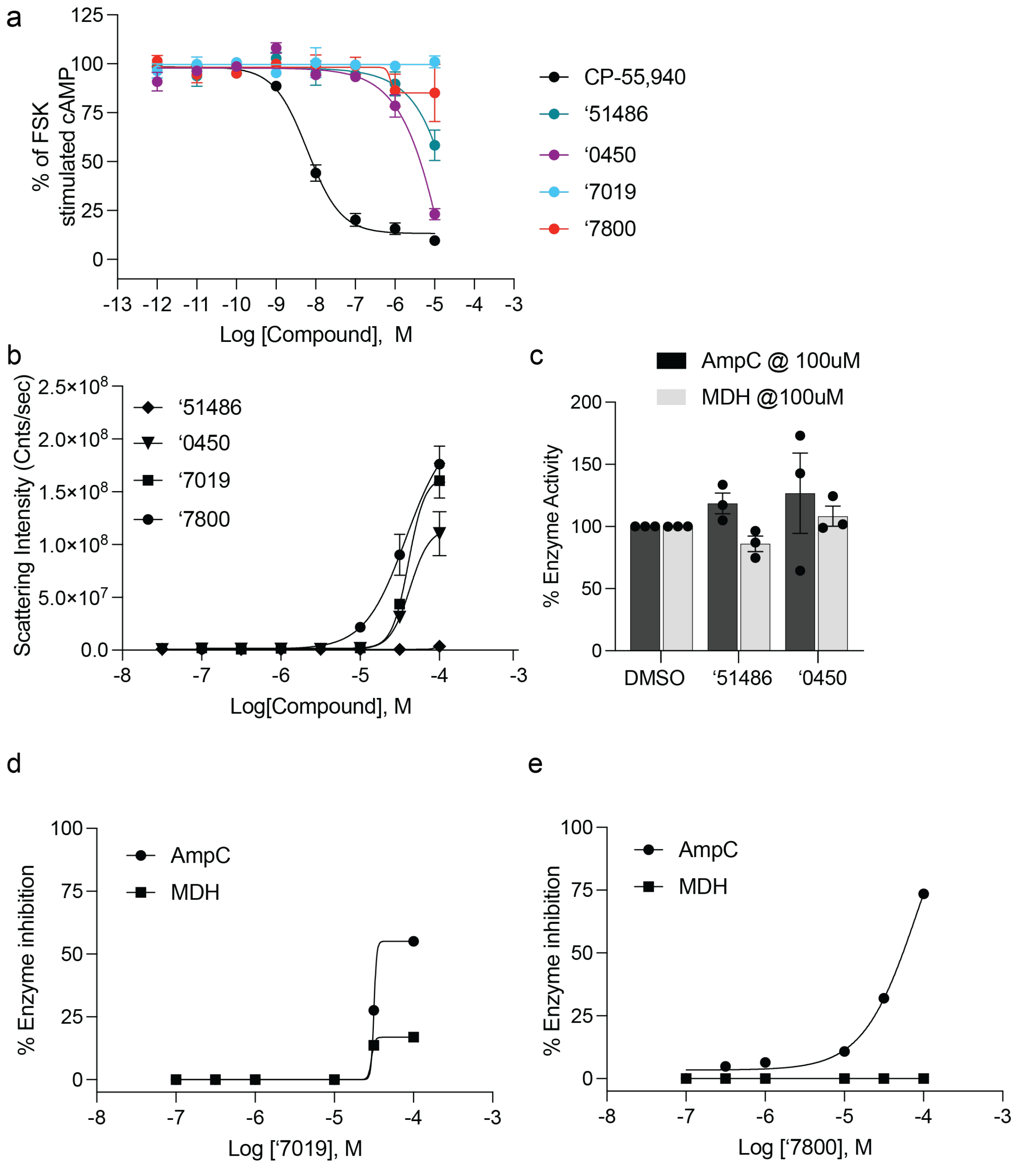


Supplementary Figure 2. Functional measurements for a subset of screening hits. a. Functional cAMP inhibition at hCB1R by the four most potent docking hits. b. Scattering intensity in dynamic light scattering experiments of colloidal aggregation. c. Inhibition of the off-target enzymes MDH and AmpC Beta-lactamase at 100 μM. d. and e. Single-point inhibition of the off-target enzymes MDH and AmpC Beta-lactamase by ‘7019 (D.) and ’7800 (E.). All data represent mean ± SEM of three independent experiments in triplicate except b. which represents one independent experiment in triplicate.

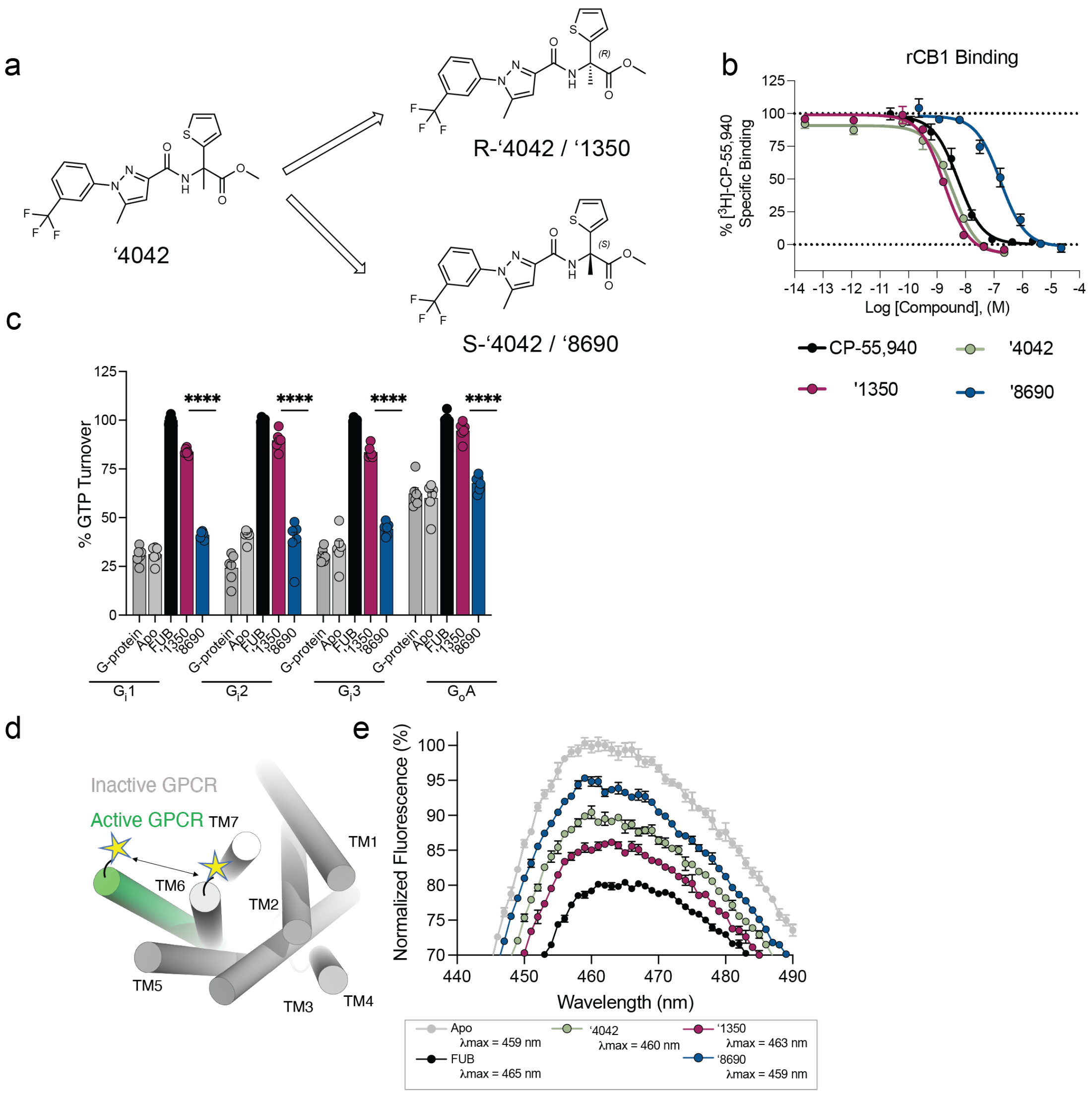


Supplementary Figure 3. Additional pharmacological characterization of '4042 and its enantiomers. a. Chiral column purification led to the separation of two independent enantiomers, '1350 and '8690. '1350 was determined to be *R*-'4042 from the Cryo-EM structure. b. Radioligand competition binding data for '4042, '1350, and '8690 versus CP-55,940. c. GTPase Glo assay characterizing GTP turnover of G proteins $G_{i1\text{-}3/o}$. d. Schematic of the environmentally sensitive fluorophore Monobromobimane (Bimane) which when site-specifically labeled (e.g. on TM6) acts as a conformational reporter. e. Compared to the apo (grey), the spectrum of full agonist MDMB-fubinaca (Fub)-bound CB1 (black) shows a decrease in intensity and a blue-shift in $\lambda_{max}$ (Apo 459 nm to Fub 465 nm). The bimane spectrum of '8690 ($\lambda_{max}$ 459 nm, blue) is more similar to apo and the spectrum of '1350 ($\lambda_{max}$ 463 nm, magenta) is closer to that of Fub. The spectrum of the racemate, '4042 (green) is between '1350 (*R*-'4042) and '8690 (*S*-'4042). All data represent mean ± SEM of three independent experiments in triplicate.

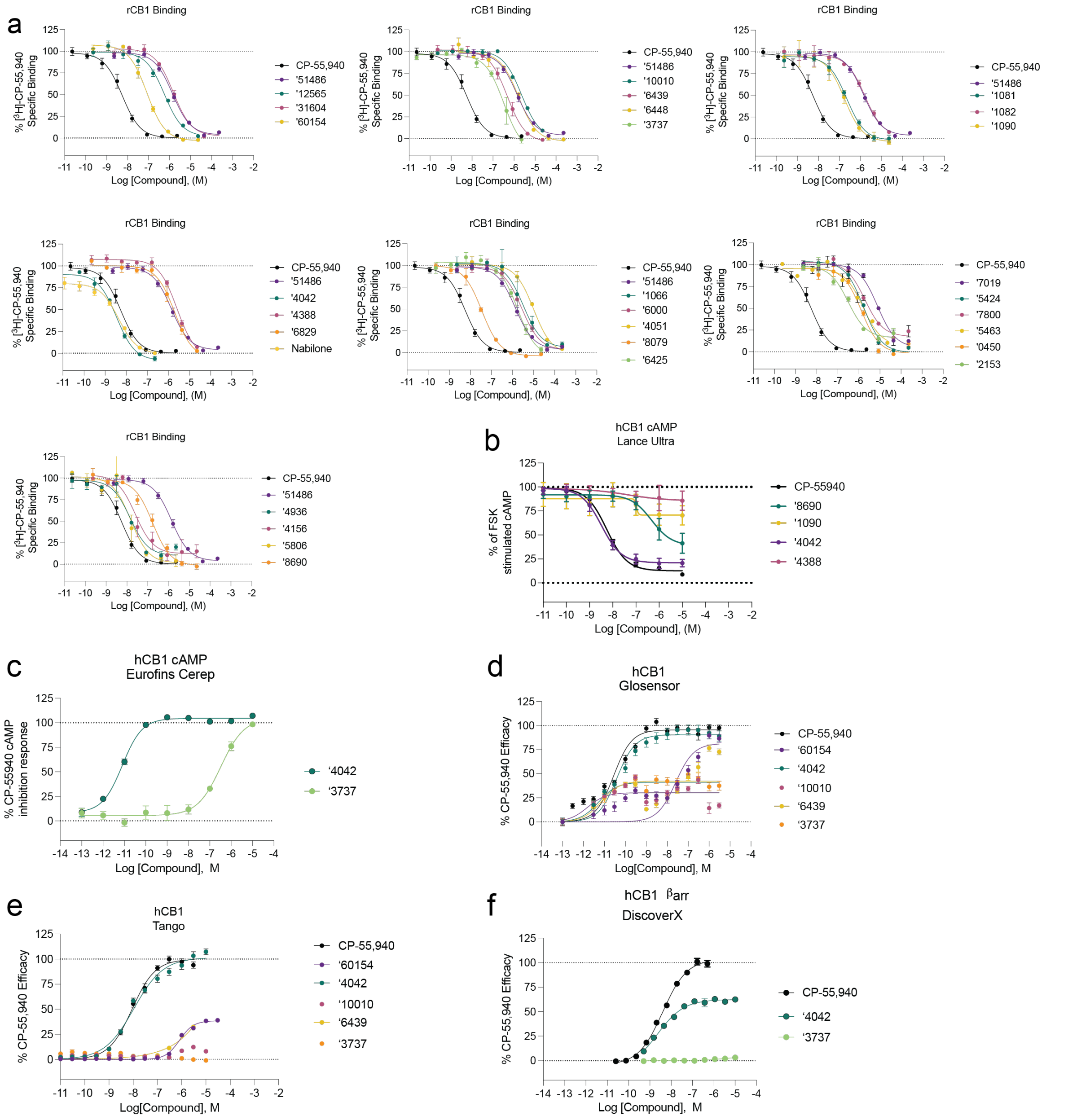


Supplementary Figure 4. CB1 binding and functional data for analogs. a. Competition binding data for primary hits and a subset of their analogs at rCB1. b.-d. Functional cAMP inhibition for a subset of analogs at hCB1 across three separate assays. e.-f. Functional ß$_{arr}$ recruitment for a subset of analogs. All data represent mean ± SEM of at least 2 independent experiments in triplicate except c. and f. which represent one independent experiment in triplicate. Best fit values can be found in Supplementary Table 2.

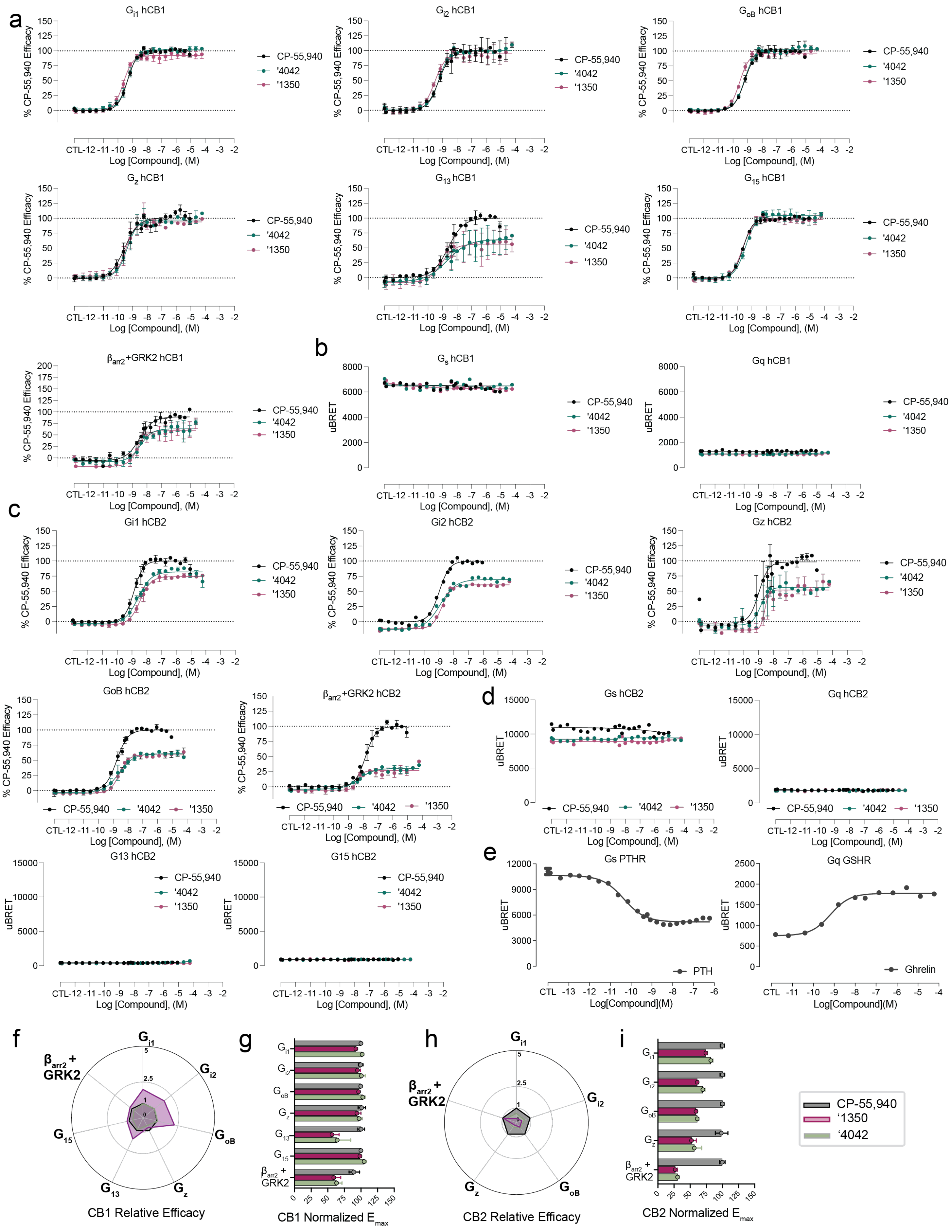

a
Gi1 hCB1
Gi2 hCB1
GoB hCB1
Gz hCB1
G13 hCB1
G15 hCB1
βarr2+GRK2 hCB1
% CP-55,940 Efficacy
Log [Compound], (M)
CP-55,940
'4042
'1350
b
Gs hCB1
Gq hCB1
uBRET
c
Gi1 hCB2
Gi2 hCB2
Gz hCB2
GoB hCB2
βarr2+GRK2 hCB2
d
Gs hCB2
Gq hCB2
G13 hCB2
G15 hCB2
e
Gs PTHR
Gq GSHR
PTH
Ghrelin
Log[Compound](M)
f
CB1 Relative Efficacy
g
CB1 Normalized Emax
h
CB2 Relative Efficacy
i
CB2 Normalized Emax
CP-55,940
'1350
'4042

Supplementary Figure 5. hCB1/2 functional data for select analogs in the bioSens-All® platform. If CP-55,940 had no response, data were left unnormalized and raw uBRET was plotted and the values were not included in follow-up analyses. If CP-55,940 had a response, data was normalized to 100% of CP-55,940 activity. The normalized versus unnormalized data is separated by subpanel. a. Normalized activity for select analogs versus a panel of sensors in hCB1-expressing cells. b. Raw BRET activity for select ana- logs versus $G_s$ and $G_q$ in hCB1-expressing cells. c. Normalized activity for select analogs versus a panel of sensors in hCB2-expressing cells. d. Raw BRET activity for select analogs versus $G_s$, $G_q$, $G_{12}$, and $G_{15}$ in hCB2-expressing cells. e. Positive control raw BRET activity for PTHR (activated by PTH) and GSHR (activated by Ghrelin), known Gs and Gq recruiting GPCRs, respectively. f. Relative efficacy ($10^{\Delta log(Emax/EC50)}$) of '1350 and '4042 compared to CP-55,940 at hCB1. g. Normalized $E_{max}$ from the experiments in f. h. Relative efficacy of '1350 and '4042 compared to CP-55940 at hCB2. i. Normalized $E_{max}$ from the experiments in h. Best fit and relative efficacy values can be found in Supplementary Tables 4, 5, & 7. Data in a.–e. represent mean ± SEM.

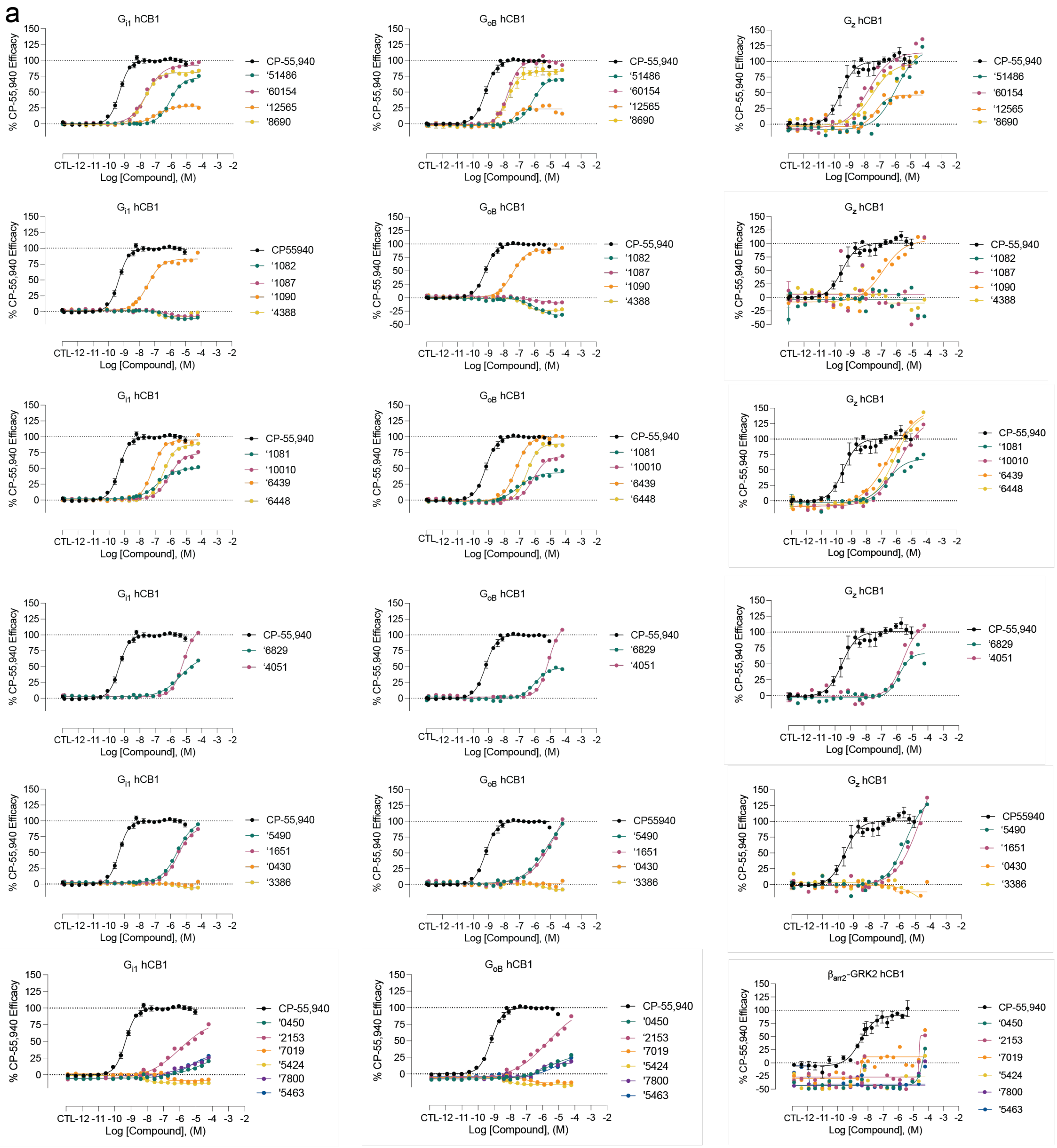


Supplementary Figure 6. hCB1 functional data for select analogs in the bioSens-All® platform. a. Normalized activity for select analogs versus a panel of sensors in hCB1-expressing cells. Best fit values can be found in Supplementary Table 3. Data represent mean ± SEM from two to three experiments.

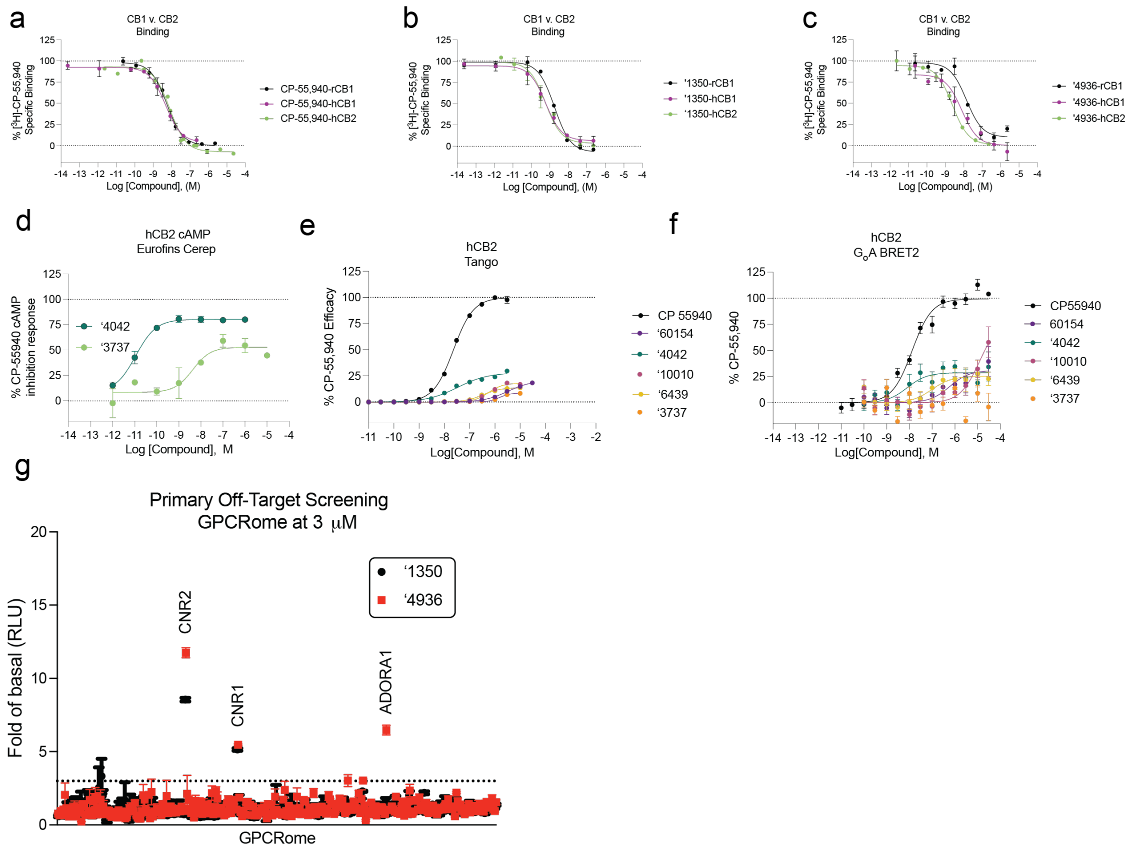


Supplementary Figure 7. Off-target CB2R binding and GPCRome functional data for select analogs. Comparison of (a.) CP-55,940 (rCB1 and hCB2: *n* = 3 experiments in triplicate; hCB1: *n* = 2 experiment in triplicate), (b.) '1350 (rCB1: *n* = 3 experiments in triplicate; hCB1/2: *n* = 2 experiments in triplicate), and (c.) '4936 (all *n* = 2 experiments in triplicate), at CB1 and CB2. d.-f. Functional cAMP inhibition for a subset of analogs at hCB2 across three separate assays. e. TANGO screens against a panel of 320 GPCRs for '1350 and '4936. For d.-g., all data represent mean ± SEM of three independent experiments in triplicate except d. which represents one independent experiment in triplicate and g. which is one independent experiment in quadruplicate. Best fit values can be found in Supplementary Table 6.

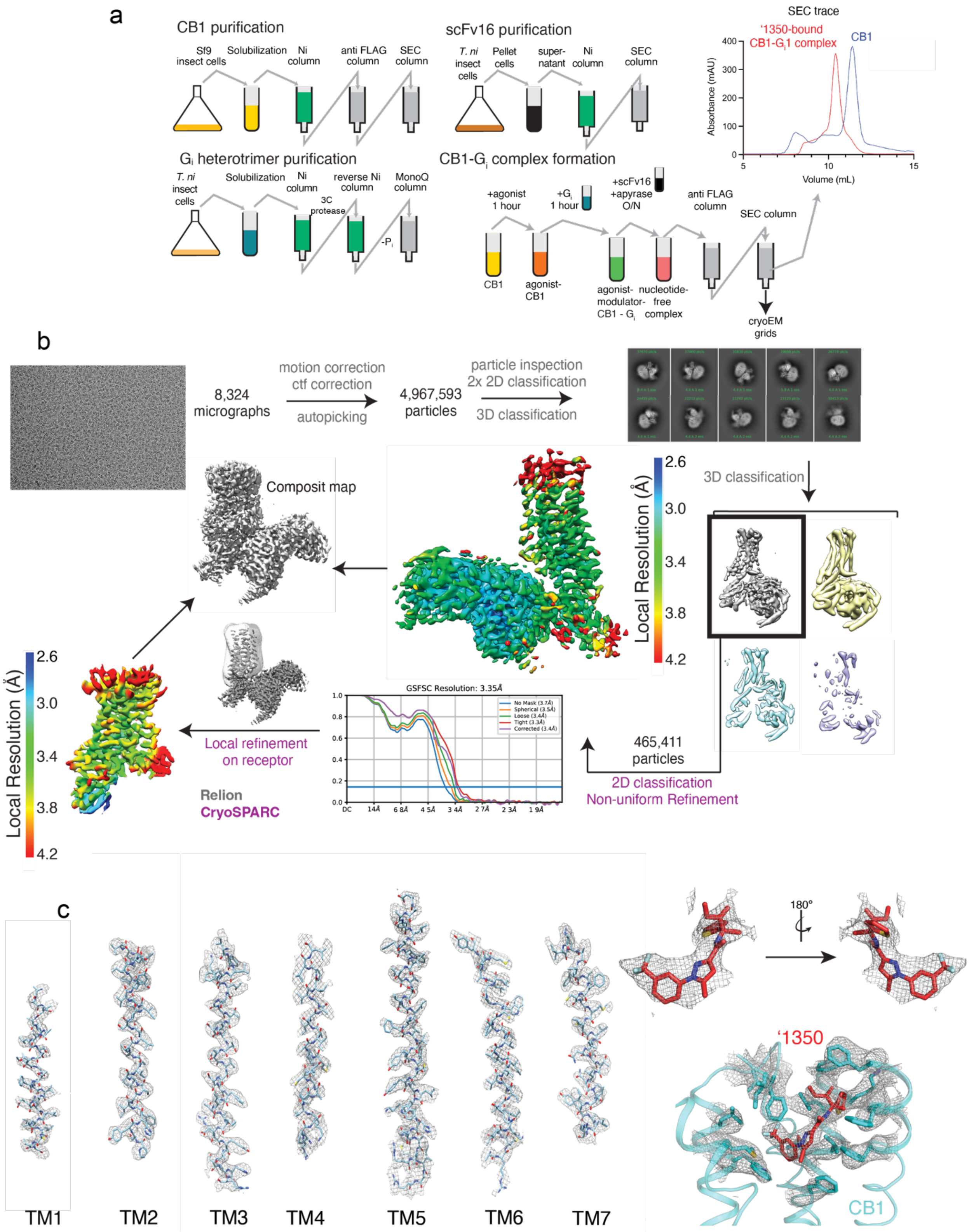
a
CB1 purification
Sf9 insect cells
Solubilization
Ni column
anti FLAG column
SEC column
scFv16 purification
T. ni insect cells
Pellet cells
super-natant
Ni column
SEC column
SEC trace
'1350-bound CB1-Gi1 complex
CB1
Absorbance (mAU)
Volume (mL)
Gi heterotrimer purification
T. ni insect cells
Solubilization
Ni column
3C protease
reverse Ni column
MonoQ column
-Pi
CB1-Gi complex formation
+agonist 1 hour
+Gi 1 hour
+scFv16 +apyrase O/N
anti FLAG column
SEC column
CB1
agonist-CB1
agonist-modulator-CB1 - Gi
nucleotide-free complex
cryoEM grids
b
8,324 micrographs
motion correction
ctf correction
autopicking
4,967,593 particles
particle inspection
2x 2D classification
3D classification
Composit map
Local Resolution (Å)
3D classification
465,411 particles
2D classification
Non-uniform Refinement
GSFSC Resolution: 3.35Å
Local refinement on receptor
Relion
CryoSPARC
c
180°
'1350
CB1
TM1
TM2
TM3
TM4
TM5
TM6
TM7

Supplementary Figure 8. Cryo-EM sample preparation and data processing. a. Purification of hCB1, scFv16, the $G_i$ heterotrimer, and complex formation protocols. b. Cryo-EM data processing flow chart of CB1, including particle selection, classifications, and density map reconstruction. c. Density for the transmembranes and around the ligand binding pocket. Details can be found in Supplementary Table 8.

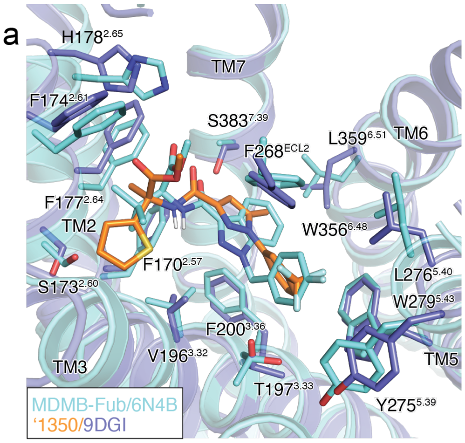

Supplementary Figure 9. Comparison of the '1350-CB1R (PDB: 9DGI) structure to MDMB-Fub-CB1R (PDB: 6N4B). a. '1350 (orange) bound to CB1R (purple compared to MDMB-Fub-CB1R (teal). Similar occupation of the binding pockets by both ligands.

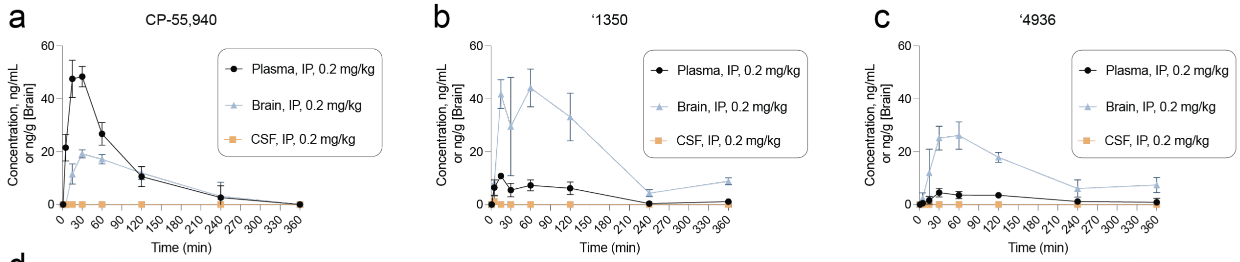


d

| Compound | Compartment | Dose Route | $T_{max}$, min | $C_{max}$ ng/mL (g) | AUC 0 → tlast ng*min/ml (g) | AUC 0 → inf ng*min/ml (g) | $T_{1/2}$ | $K_{el}$ |
|---|---|---|---|---|---|---|---|---|
| CP-55,940 | Plasma | 0.2 mg/kg IP | 30 | 48.4 | 3360 | 3990 | 41.5 | 0.0167 |
| | Brain | | 30 | 19.2 | 1700 | 3880 | 127 | 0.098 |
| | CSF | | ND | BQL | BQL | ND | ND | ND |
| '1350 | Plasma | 0.2 mg/kg IP | 15 | 10.8 | 1440 | 1510 | 102 | 0.0068 |
| | Brain | | 60 | 44.1 | 7720 | 8180 | 112 | 0.0062 |
| | CSF | | ND | BQL | BQL | ND | ND | ND |
| '4936 | Plasma | 0.2 mg/kg IP | 30 | 4.54 | 675 | 865 | 111 | 0.006 |
| | Brain | | 60 | 26.2 | 5260 | 5610 | 125 | 0.0055 |
| | CSF | | ND | BQL | BQL | ND | ND | ND |

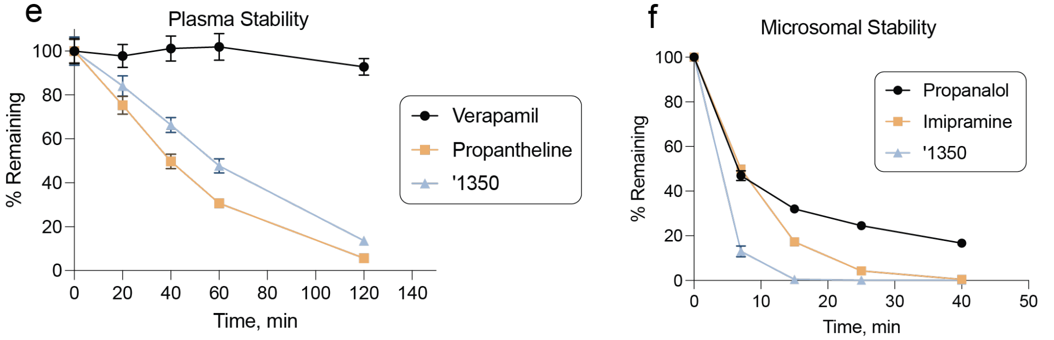


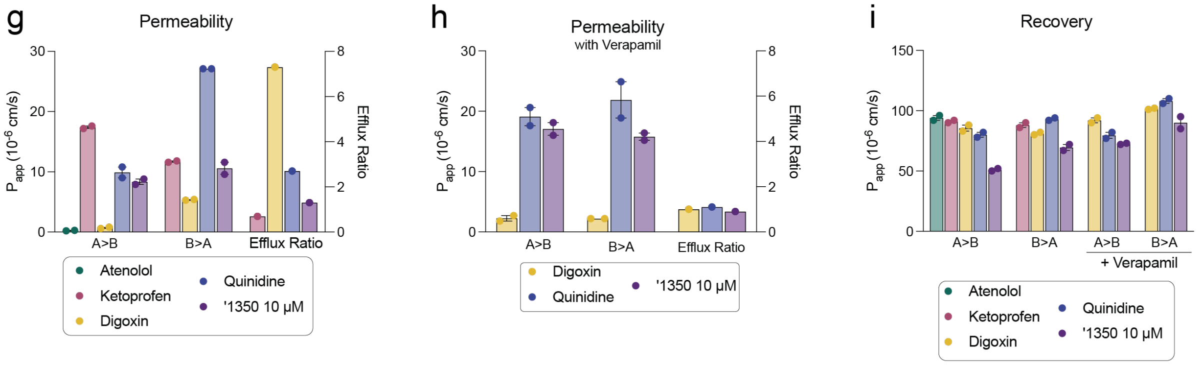

Supplementary Figure 10. Drug Metabolism and pharmacokinetic profiles. Pharmacokinetic profile of CP-55,940 (a.), ‘1350 (b.), ‘4936 (c.) after a single 0.2 mg/kg dose in brain, CSF, and plasma compartments. Data represent mean ± SEM of 3 animals per timepoint. d. Best-fit parameters of the pharmacokinetic data. e. Mouse plasma stability of ‘1350 compared to verapamil (stable) and propantheline (unstable). f. Mouse liver microsome stability of ‘1350 compared to propanalol and imipramine. g. Permeability of ‘1350 and various control molecules to MDRI-MDCKII cells. h. Permeability of ‘1350 and various control molecules to MDRI-MDCKII cells in the presence of the P-gp inhibitor verapamil. i. Recover of various molecules after the permeability assay. All data represent mean ± SEM of three independent animals per timepoint (a.-c.) or two incubations (e.-i).

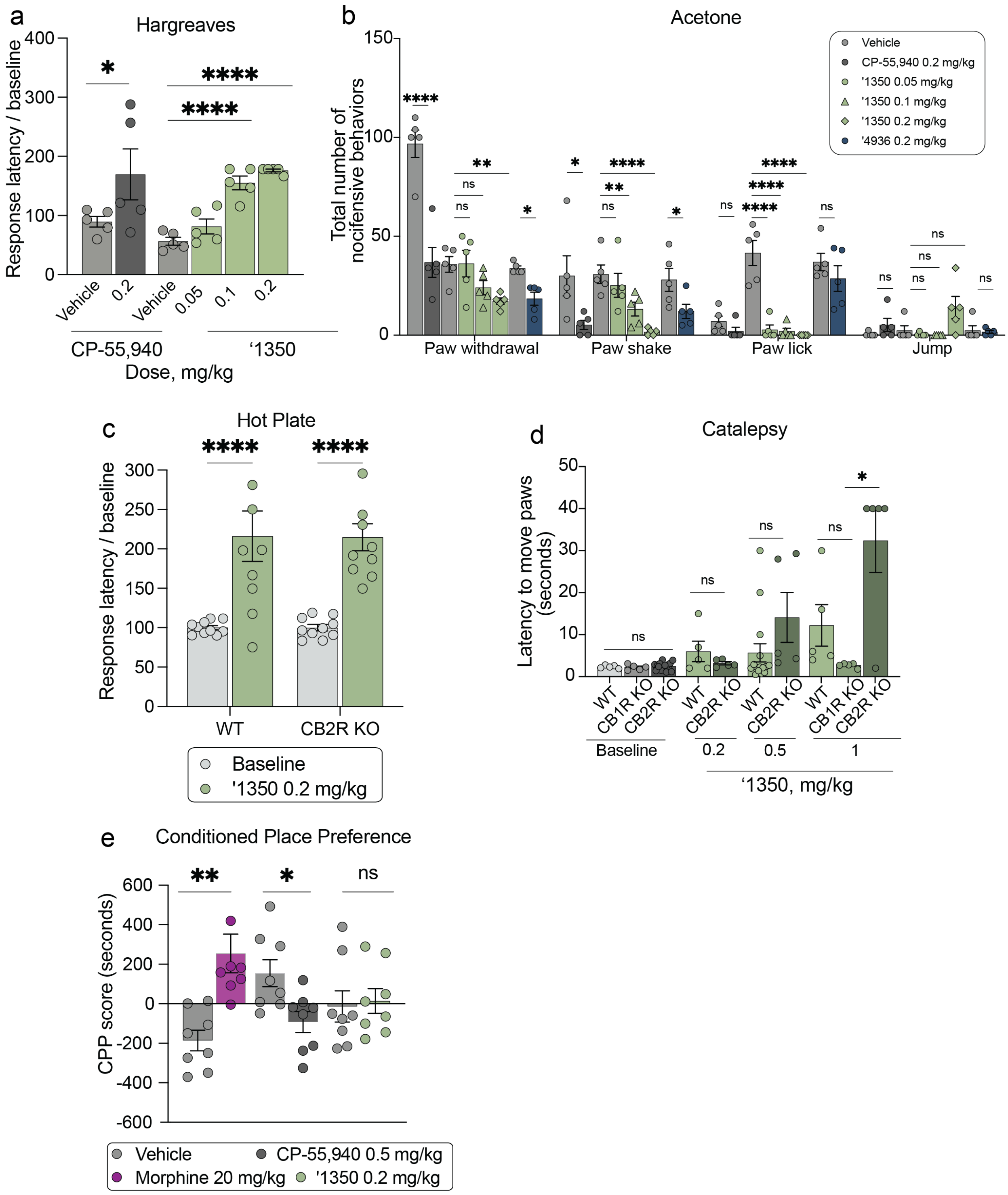
a
Hargreaves
Response latency / baseline
Vehicle
0.2
Vehicle
0.05
0.1
0.2
CP-55,940
'1350
Dose, mg/kg
b
Acetone
Total number of nocifensive behaviors
Vehicle
CP-55,940 0.2 mg/kg
'1350 0.05 mg/kg
'1350 0.1 mg/kg
'1350 0.2 mg/kg
'4936 0.2 mg/kg
Paw withdrawal
Paw shake
Paw lick
Jump
c
Hot Plate
Response latency / baseline
WT
CB2R KO
Baseline
'1350 0.2 mg/kg
d
Catalepsy
Latency to move paws (seconds)
WT
CB1R KO
CB2R KO
Baseline
0.2
0.5
1
'1350, mg/kg
e
Conditioned Place Preference
CPP score (seconds)
Vehicle
CP-55,940 0.5 mg/kg
Morphine 20 mg/kg
'1350 0.2 mg/kg

Supplementary Figure 11. Additional analgesic and side-effect profiles of ‘1350 compared to CP-55.940. a. Hargreaves assay (all $n$ = 5; one-way ANOVA, ‘1350: $F(3, 16) = 37.9$, $P <$ 0.0001; two-tailed unpaired $t$-test, CP-55,940: $t(8) = 1.8$, $P = 0.01$). Asterisks define differences to vehicle after Dunnett’s multiple comparisons post-hoc correction (‘1350) or $t$-test. b. Acetone test from Fig. 4B broken down by behavior (all $n$ = 5, two-way ANOVAs; ‘1350: behavior x dose interaction: $F(9, 64) = 8.9$, $P < 0.0001$; behavior: $F(3, 64) = 28.5$, $P <$ 0. 0001; dose: $F(3, 64) = 21.4$, $P < 0.0001$; CP-55,940: behavior x dose interaction: $F(3, 32)$ = 13.9, $P < 0.0001$; behavior: $F(3, 32) = 61.02$, $P <$ 0. 0001; dose: $F(1, 32) = 30.7$, $P < 0.0001$; ‘4936: behavior x dose interaction: $F(3, 32) = 1.59$, $P = 0.21$; behavior: $F(3, 32) = 21.94$, $P <$ 0. 0001; dose: $F(1, 32) = 12.92$, $P = 0.0011$,Asterisks define differences to vehicle after Dunnett’s (‘1350) or Šídák’s (CP-55,940 and ‘4936) multiple comparisons post-hoc correction; vehicle vs. ‘1350 0.05 mg/kg, withdrawal $P = 0.99$; shake $P = 0.60$; lick $P < 0.0001$; jump $P = 0.97$; 0.1 mg/kg, withdrawal $P = 0.083$; shake $P = 0.005$; lick $P < 0.0001$; jump $P$ = 0.95; 0.2 mg/kg, withdrawal $P = 0.0029$; shake $P < 0.0001$; lick $P < 0.0001$; jump $P = 0.09$; vs. CP-55,940, withdrawal $P < 0.0001$; shake $P = 0.01$; lick $P = 0.95$; jump $P = 0.96$; vs. ‘4936, withdrawal $P = 0.04$; shake $P = 0.03$; lick $P = 0.47$; jump $P = 0.99$. c. Wildtype (WT) vs. CB2R knockout (KO) mice (all $n$ = 10; two-way ANOVA; genotype x drug interaction and genotype: $F(1, 36) = 0.002$, $P = 0.96$; drug: $F(1, 36) = 39.8$, $P < 0.0001$; asterisks define differences to baseline after Fisher’s LSD. d. Catalepsy in WT, CB1R, and CB2R KO (all $n$ = 5 except CB2R KO baseline and WT ‘1350 0.5 mg/kg; one-way ANOVAs; baseline: $F(2, 22) = 0.15$, $P = 0.86$; 1 mg/kg: $F(2, 12) = 8.4$, $P = 0.005$, asterisks define differences to vehicle after Dunnett’s multiple comparisons post-hoc correction; two-tailed unpaired $t$-tests; 0.2 mg/kg: $t(8) = 1.1$, $P$ = 0.13; 0.5 mg/kg: $t(18) = 1.7$, $P = 0.17$). e. Conditioned Place Preference (CPP) test (all $n$ = 8; two-tailed unpaired t-tests, morphine: $t(14) = 4.0$, $P = 0.0015$; CP-55,940: $t(14) = 2.9$, $P$ = 0.01; ‘1350: $t(14) = 0.28$, $P = 0.78$). For all statistical tests: ns, not significant, *$P < 0.05$, **$P < 0.01$, ***$P < 0.001$, ****$P < 0.0001$. Data represent mean ± SEM; $n$ denotes number of independent animals.

## Supplementary Tables

Supplementary Table 1. Binding affinities for hits identified in initial CB1 docking screen.

| Compound | Global rank | rCB1 affinity[a]<br>$K_i$ [95% CI] nM<br>$pK_i$ [95% CI] | Tc[b] | Nearest ChEMBL ligand[c] |
|---|---|---|---|---|
| 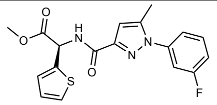<br>‘51486 | 117390 | 731 [552 – 969]<br>6.14 [6.01 –6.26] | 0.30 | 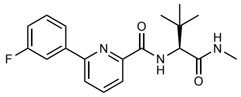<br>CHEMBL4110127 |
| 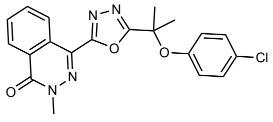<br>‘0450 | 6582 | 691 [459 – 1033]<br>6.16 [5.99 – 6.34] | 0.36 | 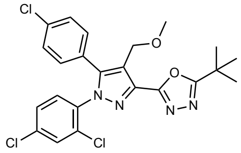<br>CHEMBL519214 |
| 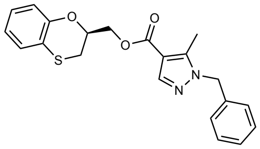<br>‘7800 | 12210 | 1007 [615 – 1654]<br>6.0 [5.78 – 6.21] | 0.28 | 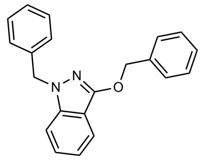<br>CHEMBL3116279 |
| 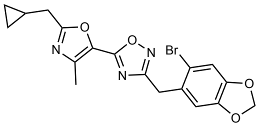<br>‘7019 | 20488 | 4039 [3027 – 5379]<br>5.39 [5.27 –5.52] | 0.24 | 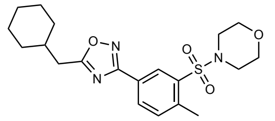<br>CHEMBL472680 |
| 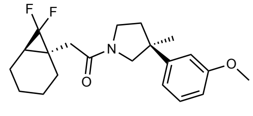<br>‘7218 | 29322 | 52.2% [24.79] | 0.31 | 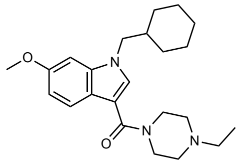<br>CHEMBL3347301 |
| 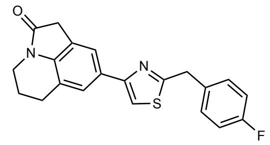<br>‘1038 | 47606 | 53.6% [2.91] | 0.28 | 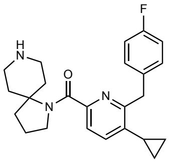<br>CHEMBL3890211 |
| 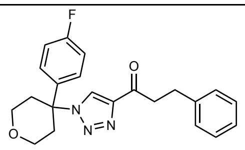<br>‘7337 | 24720 | 57.0% [3.04] | 0.29 | 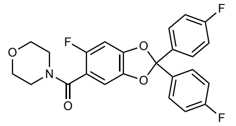<br>CHEMBL259699 |
| 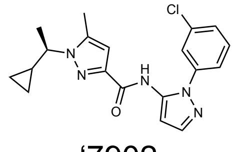<br>‘7902 | 139929 | 57.1% [0.02] | 0.31 | 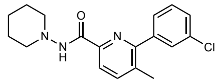<br>CHEMBL3915046 |

| | | | | |
|---|---|---|---|---|
| 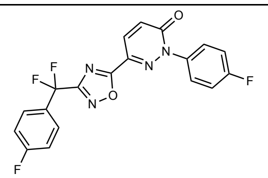 '2443 | 21964 | 51.1% [4.87] | 0.23 | 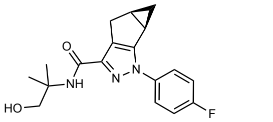 CHEMBL3354970 |

[a]Binding affinity to rCB1 represented as Ki [95% CI] and pKi [95% CI] from three independent experiments in triplicate when measured. Otherwise, % radioligand displacement [S.D] from three replicates in a single-point competition experiment at 10 μM

[b]Tanimoto coefficient (Tc) based on ECFP4 fingerprints

[c]Corresponding ChEMBL ligand with the most similar fingerprint

Supplementary Table 2. Binding affinities and functional activities for active analogs at CB1.

| Compound | | CB1[a] binding<br>$K_i$ [95% CI] (nM)<br>$pK_i$ [95% CI]<br>$E_{max}$ [SEM] | hCB1 Lance Ultra cAMP<br>$EC_{50}$ [95% CI] (nM)<br>$pEC_{50}$ [95% CI]<br>$E_{max}$ [SEM] | hCB1 Cerep cAMP<br>$EC_{50}$ [95% CI] (nM)<br>$pEC_{50}$ [95% CI]<br>$E_{max}$ [95% CI] | hCB1 Glosensor cAMP<br>$EC_{50}$ [95% CI] (nM)<br>$pEC_{50}$ [95% CI]<br>$E_{max}$ [95% CI] | hCB1 Tango ß-arrestin recruitment<br>$EC_{50}$ [95% CI] (nM)<br>$pEC_{50}$ [95% CI]<br>$E_{max}$ [95% CI] | hCB1 DiscoverX ß-arrestin recruitment<br>$EC_{50}$ [95% CI] (nM)<br>$pEC_{50}$ [95% CI]<br>$E_{max}$ [95% CI] |
|---|---|---|---|---|---|---|---|
| 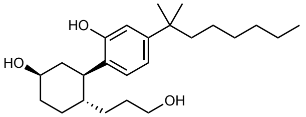<br>CP–55,940 | rCB1 | 2.9 [2.05 – 4.2]<br>8.5 [8.4 – 8.7]<br>98% [3.5] | 5.5 [4.4 – 6.8]<br>8.3 [8.2 – 8.4]<br>86% [2.0] | -- | 0.028 [0.02 – 0.04]<br>10.6 [10.5 – 10.7]<br>96% [93 – 99] | 8.9 [7.5 – 10.6]<br>8.1 [8.0 – 8.1]<br>100% [96 – 104] | 4.0 [3.2 – 4.9]<br>8.4 [8.3 – 8.5]<br>108% [99 – 109] |
| | hCB1 | 2.5 [1.7 – 3.5]<br>8.6 [8.5 – 8.8]<br>88.4% [3.2] | | | | | |
| 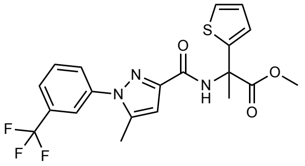<br>‘4042 | | 1.86 [1.37 – 2.52]<br>8.7 [8.6 – 8.9]<br>99% [3.0] | 3.3 [1.9 – 5.6]<br>8.5 [8.3 – 8.7]<br>78% [78 – 79] | 0.008 [0.006 – 0.01]<br>11.1 [11.0 – 11.2]<br>96% [102 – 107] | 0.039 [2.9 – 5.4]<br>10.4 [10.3 – 10.5]<br>91% [87 – 94] | 10.7 [8.7 – 13.3]<br>8.0 [7.9 – 8.1]<br>102% [98 – 105] | 2.3 [2.5 –4.8]<br>8.7 [8.3 – 9.6]<br>71% [60 – 65] |
| 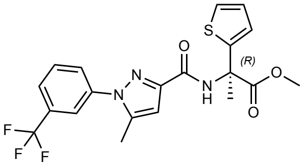<br>‘1350 | rCB1 | 0.95 [.74 – 1.2]<br>9.02 [8.9 – 9.1]<br>106% [2.9] | 1.6 [0.7 – 3.6]<br>8.8 [8.4 – 9.2]<br>77% [6.5] | -- | -- | -- | -- |
| | hCB1 | 0.32 [.17 – .6]<br>9.5 [9.2 – 9.8]<br>88% [4.7] | | | | | |
| 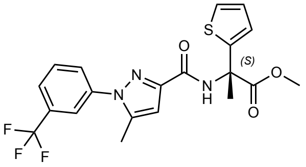<br>‘8690 | | 90.2 [56.7 – 143]<br>7.1 [6.9 – 7.3]<br>100% [4.0] | 473 [109 – 1822]<br>6.3 [5.8 – 7.0]<br>53% [8.2] | -- | -- | -- | -- |

| | | | | | | |
|---|---|---|---|---|---|---|
| 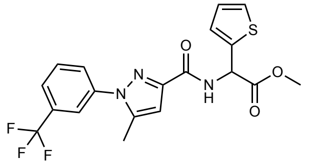<br>‘60154 | 44.3 [33.9 – 58.0]<br>7.4 [7.2 – 7.5]<br>110% [2.9] | 351 [93]<br>6.5 [7.0]<br>67% [13.2] | -- | 25.2 [16 – 40]<br>7.6 [7.4 – 7.8]<br>82% [74 – 189] | 819 [718 – 934]<br>6.1 [6.0 – 6.1]<br>39% [37 – 40] | -- |
| 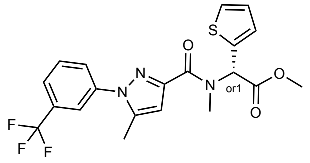<br>‘1066 | 1719 [736 – 4048]<br>5.8 [5.4 – 6.1]<br>97% [8.0] | -- | -- | -- | -- | -- |
| 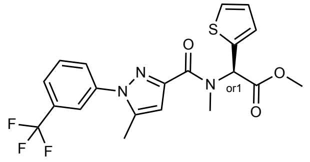<br>‘6000 | 1455 [943 – 2249]<br>5.8 [5.7 – 6.0]<br>96% [4.0] | -- | -- | -- | -- | -- |
| 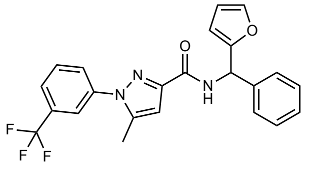<br>‘1081 | 116 [76.3 – 178]<br>6.9 [6.8 – 7.1]<br>96% [4.0] | -- | -- | -- | -- | -- |
| 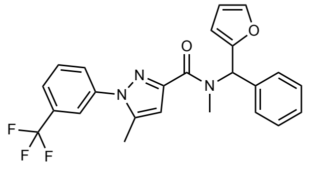<br>‘1082 | 850 [488 – 1491]<br>6.1 [5.8 – 6.3]<br>94% [5.7] | -- | -- | -- | -- | -- |

| | | | | | | | |
|---|---|---|---|---|---|---|---|
| 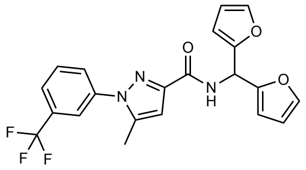<br>‘1090 | 90.8 [42.7 – 192]<br>7.0 [6.7 – 7.4]<br>99% [7.13] | | N.D. | -- | -- | -- | -- |
| 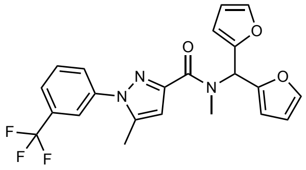<br>‘4388 | 1360 [998 – 1857]<br>5.9 [5.7 – 6.0]<br>113% [4.4] | | N.D. | -- | -- | -- | -- |
| 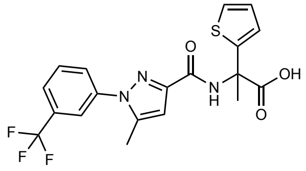<br>‘4051 | 5328 [3774–7507]<br>5.3 [5.1 – 5.4]<br>103% [3.8] | | -- | -- | -- | -- | -- |
| 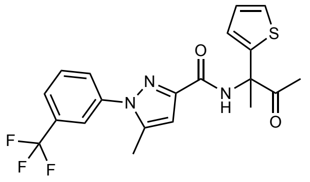<br>‘4156 | 13.5 [6.3 – 30.1]<br>7.9 [7.5 – 8.2]<br>91% [7.5] | | -- | -- | -- | -- | -- |
| 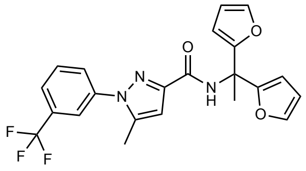<br>‘4936 | rCB1 | 7.5 [3.9 – 14.3]<br>8.1 [7.9 – 8.4]<br>88% [6.3] | 4.79 [2.4]<br>8.3 [8.6]<br>65% [4.5] | -- | -- | -- | -- |
| | hCB1 | 4.1 [1.2 – 1.5]<br>8.4 [7.9 – 8.8]<br>83% [6.5] | | | | | |

| | | | | | | |
|---|---|---|---|---|---|---|
| 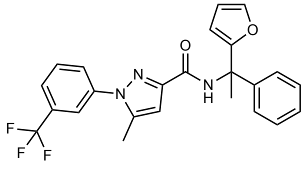<br>‘5806 | 8.0 [3.0 – 21.8]<br>8.1 [7.7 – 8.5]<br>100% [10.5] | -- | -- | -- | -- | -- |
| 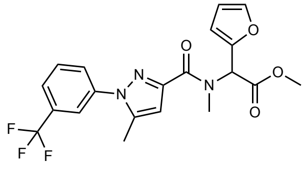<br>‘6425 | 934 [583 – 1501]<br>6.0 [5.8 – 6.2]<br>108% [5.8] | -- | -- | -- | -- | -- |
| 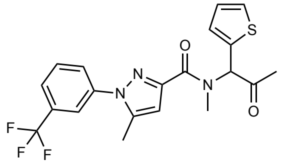<br>‘6829 | 1046 [669 – 1643]<br>6.0 [5.8 – 6.2]<br>106% [5.8] | -- | -- | -- | -- | -- |
| 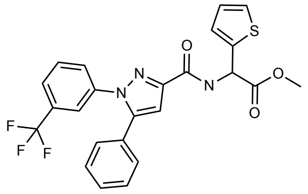<br>‘8079 | 18.5 [13.8 – 25.0]<br>7.7 [7.6 – 7.9]<br>104% [3.0] | -- | -- | -- | -- | -- |
| 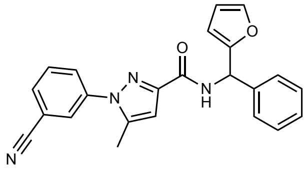<br>‘12565 | 301 [195 – 462]<br>6.5 [6.3 – 6.7]<br>97% [4.0] | -- | -- | -- | -- | -- |

| | | | | | | |
|---|---|---|---|---|---|---|
| 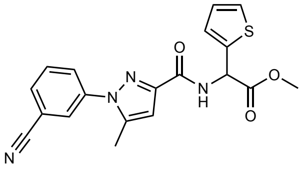 '31604 | 801 [596 –1,076]<br>6.1 [6.0 – 6.2]<br>100% [2.8] | -- | -- | -- | -- | -- |
| 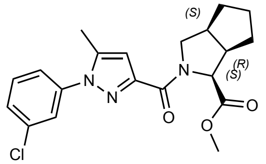 '10010 | 1,196 [952 – 1,505]<br>5.9 [5.8 – 6.0]<br>101% [2.9] | -- | -- | N.D | > 10,000 | -- |
| 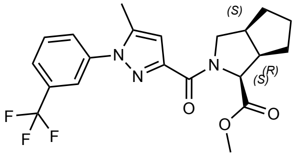 '6439 | 251 [173 – 364]<br>6.6 [6.4 – 6.8]<br>106% [3.9] | -- | -- | N.D. | > 10,000 | -- |
| 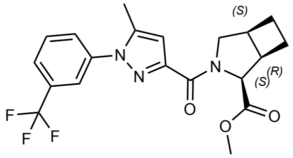 '6448 | 866 [564 – 1,317]<br>6.1 [5.9 – 6.3]<br>103% [4.1] | -- | -- | -- | -- | -- |
| 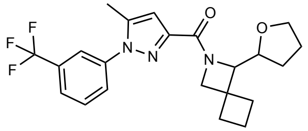 '3737 | 173 [94 – 322]<br>6.76 [6.5 – 7.03]<br>112% [8.8] | -- | 326 [168 – 1044]<br>6.5 [6.0 – 6.8]<br>99% [91 – 135] | N.D | > 10,000 | > 10,000 |
| 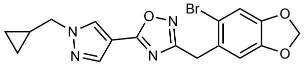 '5424 | 876 [683 – 1123]<br>6.1 [6.0 – 6.2]<br>104% [2.6] | -- | -- | -- | -- | -- |

| | | | | | | |
|---|---|---|---|---|---|---|
| 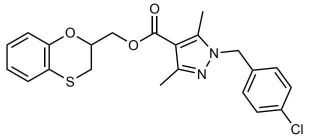 '5463 | 825 [396 – 1,755]<br>6.1 [5.8 – 6.4]<br>88% [6.6] | -- | -- | -- | -- | -- |
| 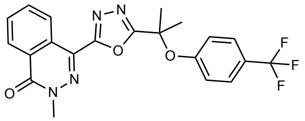 '2153 | 163 [90 – 287]<br>6.79 [6.5 – 7.0]<br>82.6% [4.5] | -- | -- | -- | -- | -- |
| 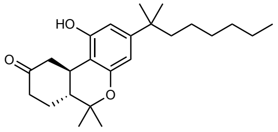 Nabilone | 2.3 [1.4 – 3.8]<br>8.6 [8.4 – 8.9]<br>81% [3.7] | -- | -- | -- | -- | -- |

N.D. = best fit values were not determined due to compound inactivity or poor data quality

-- Not tested

[a]All binding data is rCB1 unless otherwise labeled

Supplementary Table 3. Functional activities for select analogs versus a variety of transducers and hCB1 in the bioSens-All® platform.

| Compound | | hCB1 $G_{i1}$ | hCB1 $G_{oB}$ | hCB1 $G_z$ |
|---|---|---|---|---|
| CP–55,940 | $EC_{50}$ [95% CI] (nM) | 0.46 [0.4 – 0.5] | 0.63 [0.6 – 0.7] | 0.28 [0.18 – 0.5] |
| | $pEC_{50}$ [95% CI] | 9.3 [9.3 – 9.4] | 9.2 [9.2 – 9.3] | 9.6 [9.4 – 9.8] |
| | $E_{max}$ [95% CI] | 100 [98 – 102] | 100 [99 – 101] | 102 [102 – 104] |
| ‘51486 | $EC_{50}$ [95% CI] (nM) | 849 [745– 947] | 711 [579– 873] | 1118 [363– 3447] |
| | $pEC_{50}$ [95% CI] | 6.1 [6.0 – 6.1] | 6.2 [6.1 – 6.2] | 6.0 [5.5 – 6.4] |
| | $E_{max}$ [95% CI] | 73 [70 – 75] | 74 [70 – 75] | 130 [996 – 163] |
| ‘60154 | $EC_{50}$ [95% CI] (nM) | 2.5 [1.9 – 3.4] | 18.4 [15.8– 21.5] | 17.5 [7.5 – 41] |
| | $pEC_{50}$ [95% CI] | 7.6 [7.5 – 7.7] | 7.7 [7.7 – 7.8] | 7.8 [7.4 – 8.1] |
| | $E_{max}$ [95% CI] | 92 [90 – 94] | 100 [98 – 100] | 121 [103 – 140] |
| ‘1081 | $EC_{50}$ [95% CI] (nM) | 150 [116 – 197] | 91.7 [55 – 153] | 225 [73 – 694] |
| | $pEC_{50}$ [95% CI] | 6.8 [6.7 – 6.9] | 7.0 [6.8 – 7.3] | 6.7 [6.2 – 7.1] |
| | $E_{max}$ [95% CI] | 49 [48 – 51] | 45 [43 –47] | 72 [55 –89] |
| ‘1082 | $EC_{50}$ [95% CI] (nM) | | | |
| | $pEC_{50}$ [95% CI] | [a]N.D. | N.D. | N.D. |
| | $E_{max}$ [95% CI] | | | |
| ‘1087 | $EC_{50}$ [95% CI] (nM) | | | |
| | $pEC_{50}$ [95% CI] | N.D. | N.D. | N.D. |
| | $E_{max}$ [95% CI] | | | |
| ‘1090 | $EC_{50}$ [95% CI] (nM) | 35.6 [28.5 – 45] | 37.3 [30 – 47] | 126 [45 – 354] |
| | $pEC_{50}$ [95% CI] | 7.5 [7.3 – 7.5] | 7.4 [7.3 – 7.5] | 6.9 [6.5 – 7.4] |
| | $E_{max}$ [95% CI] | 81 [80 –82] | 90 [88 –92] | 115 [92 – 137] |
| ‘4388 | $EC_{50}$ [95% CI] (nM) | | | |
| | $pEC_{50}$ [95% CI] | N.D. | N.D. | N.D. |
| | $E_{max}$ [95% CI] | | | |
| ‘6829 | $EC_{50}$ [95% CI] (nM) | 4056 [2417 – 9228] | 1011 [706 – 1449] | 1347 [720 – 2522] |
| | $pEC_{50}$ [95% CI] | 5.4 [5.0 – 5.6] | 6.0 [5.8 – 6.2] | 5.9 [6.0 – 6.2] |
| | $E_{max}$ [95% CI] | 68 [57 – 77] | 49 [45 – 53] | 70 [57 –83] |
| ‘4051 | $EC_{50}$ [95% CI] (nM) | 6523 [5770 – 7511] | 7988 [6709 – 9511] | 2431 [1069 – 5531] |
| | $pEC_{50}$ [95% CI] | 5.2 [5.1 – 5.2] | 5.1 [5.0 – 5.2] | 5.6 [5.3 – 6.0] |
| | $E_{max}$ [95% CI] | 108 [104 – 113] | 115 [106 – 121] | 114 [86 –141] |
| ‘12565 | $EC_{50}$ [95% CI] (nM) | 104 [79 – 138] | 44.8 [27 – 73] | 54.6 [22.1 – 135] |
| | $pEC_{50}$ [95% CI] | 7.0 [6.9 – 7.1] | 7.4 [7.1 – 7.6] | 7.3 [6.9 – 7.7] |
| | $E_{max}$ [95% CI] | 28 [27 – 28] | 25 [24 –26] | 51 [41 – 61] |
| ‘10010 | $EC_{50}$ [95% CI] (nM) | 814 [717 – 932] | 801 [582– 1102] | 1396 [506 – 3853] |
| | $pEC_{50}$ [95% CI] | 6.1 [6.0 – 6.1] | 6.1 [6.0 – 6.2] | 5.9 [5.4 – 6.3] |
| | $E_{max}$ [95% CI] | 74 [70 – 74] | 71 [67 – 75] | 144 [113 –175] |
| ‘6439 | $EC_{50}$ [95% CI] (nM) | 65.5 [56.8 – 755] | 60.9 [52.9 – 70] | 339 [96 – 1195] |
| | $pEC_{50}$ [95% CI] | 7.2 [7.1 – 7.2] | 7.2 [7.2 – 7.3] | 6.5 [5.9 – 7.0] |
| | $E_{max}$ [95% CI] | 95 [93 – 96] | 100 [99 – 101] | 162 [125 –199] |
| ‘6448 | $EC_{50}$ [95% CI] (nM) | 345 [321 – 372] | 310 [267 – 360] | 728 [381 – 1393] |
| | $pEC_{50}$ [95% CI] | 6.5 [6.4 – 6.5] | 6.5 [6.4 – 6.6] | 6.1 [5.9 – 6.4] |
| | $E_{max}$ [95% CI] | 87 [86 – 88] | 90 [87 – 91] | 151 [130 –172] |
| ‘5490 | $EC_{50}$ [95% CI] (nM) | 2804 [2436 – 3285] | 6729 [2678 – 16910] | 2470 [998 – 6108] |
| | $pEC_{50}$ [95% CI] | 5.6 [5.5 – 5.6] | 5.2 [4.8 – 5.6] | 5.6 [5.2 – 6.1] |
| | $E_{max}$ [95% CI] | 98 [94 – 102] | 118 [94 – 143] | 140 [9109 – 172] |
| ‘1651 | $EC_{50}$ [95% CI] (nM) | 3026 [2486 – 3822] | 19780 [4343 – 90080] | 30840 [1133 – 839000] |
| | $pEC_{50}$ [95% CI] | 5.5 [5.4 – 5.6] | 4.7 [4.1 – 5.4] | 4.5 [3.1– 6.0] |
| | $E_{max}$ [95% CI] | 89 [84 – 94] | 152 [94 – 208] | 231 [34 – 428] |
| ‘0430 | $EC_{50}$ [95% CI] (nM) | | | |
| | $pEC_{50}$ [95% CI] | N.D. | N.D. | N.D. |
| | $E_{max}$ [95% CI] | | | |
| ‘3386 | $EC_{50}$ [95% CI] (nM) | N.D. | N.D. | N.D. |

| | | | | |
|---|---|---|---|---|
| | $pEC_{50}$ [95% CI]<br>$E_{max}$ [95% CI] | | | |
| ‘7019 | $EC_{50}$ [95% CI] (nM)<br>$pEC_{50}$ [95% CI]<br>$E_{max}$ [95% CI] | N.D. | N.D. | [b]-- |
| ‘5424 | $EC_{50}$ [95% CI] (nM)<br>$pEC_{50}$ [95% CI]<br>$E_{max}$ [95% CI] | N.D. | N.D. | -- |
| ‘7800 | $EC_{50}$ [95% CI] (nM)<br>$pEC_{50}$ [95% CI]<br>$E_{max}$ [95% CI] | 6793 [983]<br>5.2 [6.0]<br>34 [24] | 597 [398 – 1017]<br>6.2 [5.9 – 6.4]<br>21 [20 – 23] | -- |
| ‘5463 | $EC_{50}$ [95% CI] (nM)<br>$pEC_{50}$ [95% CI]<br>$E_{max}$ [95% CI] | 4941 [1763 – 26860000]<br>5.3 [3.4 – 5.8]<br>31 [24 – 88] | 7472 [1125]<br>5.1 [6.0]<br>32 [22] | -- |
| ‘0450 | $EC_{50}$ [95% CI] (nM)<br>$pEC_{50}$ [95% CI]<br>$E_{max}$ [95% CI] | 56310 [2177]<br>4.3 [5.7]<br>49 [26] | 2509<br>5.6 [3.0 – 6.2]<br>36 [30 – 117] | -- |
| ‘2153 | $EC_{50}$ [95% CI] (nM)<br>$pEC_{50}$ [95% CI]<br>$E_{max}$ [95% CI] | 1011 [371 – 6576]<br>6.0 [5.2 – 6.4]<br>89 [78 – 118] | 2061 [620 – 25390]<br>5.7 4.6 – 6.2]<br>109 [91 – 162] | -- |
| ‘8690 | $EC_{50}$ [95% CI] (nM)<br>$pEC_{50}$ [95% CI]<br>$E_{max}$ [95% CI] | 19.1 [14.4 – 25.8]<br>7.72 [7.6 – 7.8]<br>82 [77 – 85] | 18 [14 – 23]<br>7.8 [7.7 – 7.9]<br>83 [80 – 87] | 33 [15 – 117]<br>7.5 [6.9 – 7.8]<br>98 [86 – 121] |

[a]N.D. = best fit values were not able to be determined due to compound inactivity or poor data quality
[b]-- Not tested

Supplementary Table 4. Detailed functional activities for select analogs and control versus a variety of transducers and hCB1 in the bioSens-All® platform.

| Compound | | hCB1 $G_{i1}$ | hCB1 $G_{i2}$ | hCB1 $G_{oB}$ | hCB1 $G_z$ | hCB1 $G_{13}$ | hCB1 $G_{15}$ | hCB1 Barr2 + GRK2 |
|---|---|---|---|---|---|---|---|---|
| CP–55,940 | $EC_{50}$ [95% CI] (nM) | 0.46 [0.4 – 0.5] | 0.55 [0.4 – 0.7] | 0.63 [0.6 – 0.7] | 0.28 [0.18 – 0.5] | 2.4 [1.65 – 3.4] | 0.26 [0.22 – 2.9] | 3.1 [1.97 – 4.8] |
| | $pEC_{50}$ [95% CI] | 9.34 [9.3 – 9.4] | 9.26 [9.1 – 9.4] | 9.20 [9.2 – 9.3] | 9.55 [9.4 – 9.8] | 8.62 [8.5 – 8.8] | 9.59 [9.5 – 9.7] | 8.51 [8.3 – 8.7] |
| | $E_{max}$ [95% CI] | 100 [98 – 102] | 100 [96 – 103] | 100 [98 – 101] | 101 [96 – 107] | 100 [95 – 105] | 100 [98 – 102] | 89 [82 – 98] |
| ‘4042 | $EC_{50}$ [95% CI] (nM) | 0.48 [0.4 – 0.6] | 0.56 [0.4 – 0.9] | 0.64 [0.5 – 0.8] | 0.43 [0.3 – 0.6] | 2.1 [0.5 – 9.6] | 0.37 [0.28 – 0.49] | 3.6 [2.1 – 6.8] |
| | $pEC_{50}$ [95% CI] | 9.32 [9.2 – 9.4] | 9.25 [9.0 – 9.5] | 9.20 [9.1 – 9.3] | 9.37 [9.3 – 9.4] | 8.69 [8.0 – 9.3] | 9.43 [9.3 – 9.6] | 8.44 [8.2 – 8.7] |
| | $E_{max}$ [95% CI] | 102 [100 – 104] | 101 [95 – 106] | 103 [99 – 106] | 97 [93 – 102] | 64 [54 – 84] | 105 [102 – 108] | 72 [71 – 74] |
| ‘1350 | $EC_{50}$ [95% CI] (nM) | 0.23 [0.18 – 0.3] | 0.28 [0.19 – 0.4] | 0.29 [0.24 – 0.34] | 0.35 [0.22 – 0.53] | 0.83 [0.23 – 2.7] | 0.22 [0.18 – 0.27] | 2.2 [1.1 – 4.4] |
| | $pEC_{50}$ [95% CI] | 9.63 [9.5 – 9.7] | 9.54 [9.4 – 9.7] | 9.54 [9.5 – 9.6] | 9.46 [9.3 – 9.7] | 9.08 [8.6 – 9.6] | 9.66 [9.6 – 9.8] | 8.66 [8.4 – 9.0] |
| | $E_{max}$ [95% CI] | 92 [90 – 95] | 95 [90 – 99] | 98 [94 – 98] | 94 [88 – 100] | 56 [48 – 67] | 99 [96 – 101] | 63 [60 – 72] |

Supplementary Table 5. Relative efficacy for ‘4042 and ‘1350 vs. CP-55,940.

| Target | Sensor | Compound | Mean log ($E_{max}/EC_{50}$) | SEM log ($E_{max}/EC_{50}$) | Mean Δlog ($E_{max}/EC_{50}$) | SEM Δlog ($E_{max}/EC_{50}$) | t-test to CP-55,940[a] | RE[b] |
|---|---|---|---|---|---|---|---|---|
| hCB1 | $G_{i1}$ | CP-55,940 | 9.34 | 0.07 | 0.00 | 0.09 | | 1.00 |
| | | '4042 | 9.33 | 0.14 | -0.01 | 0.15 | $t(5) = 0.15$, $P = 0.9$ | 0.97 |
| | | '1350 | 9.59 | 0.06 | 0.30 | 0.09 | $t(5) = 11.9$, $P < 0.0001$ | 1.98 |
| | $G_{i2}$ | CP-55,940 | 9.24 | 0.13 | 0.00 | 0.18 | | 1.00 |
| | | '4042 | 9.25 | 0.03 | 0.01 | 0.13 | $t(2) = 0.23$, $P = 0.8$ | 1.01 |
| | | '1350 | 9.52 | 0.03 | 0.28 | 0.13 | $t(2) = 10.6$, $P = 0.009$ | 1.89 |
| | $G_{oB}$ | CP-55,940 | 9.19 | 0.07 | 0.00 | 0.10 | | 1.00 |
| | | '4042 | 9.21 | 0.09 | 0.02 | 0.11 | $t(5) = 0.25$, $P = 0.8$ | 1.04 |
| | | '1350 | 9.09 | 0.44 | 0.35 | 0.44 | $t(5) = 19.5$, $P < 0.0001$ | 2.24 |
| | $G_z$ | CP-55,940 | 9.53 | 0.23 | 0.00 | 0.33 | | 1.00 |
| | | '4042 | 9.32 | 0.19 | -0.21 | 0.30 | $t(4) = 1.1$, $P = 0.3$ | 0.62 |
| | | '1350 | 9.42 | 0.22 | -0.11 | 0.32 | $t(3) = 0.68$, $P = 0.5$ | 0.77 |
| | $G_{13}$ | CP-55,940 | 8.63 | 0.23 | 0.00 | 0.32 | | 1.00 |
| | | '4042 | 8.59 | 0.25 | -0.04 | 0.33 | $t(2) = 0.16$, $P = 0.9$ | 0.91 |
| | | '1350 | 8.85 | 0.05 | 0.22 | 0.23 | $t(2) = 4.5$, $P = 0.046$ | 1.64 |
| | $G_{15}$ | CP-55,940 | 9.59 | 0.02 | 0.00 | 0.02 | | 1.00 |
| | | '4042 | 9.46 | 0.12 | -0.13 | 0.12 | $t(2) = 1.09$, $P = 0.38$ | 0.74 |
| | | '1350 | 9.65 | 0.06 | 0.06 | 0.06 | $t(2) = 0.94$, $P = 0.45$ | 1.14 |
| | $ß_{arr2}$+ GRK2 | CP-55,940 | 8.28 | 0.25 | 0.00 | 0.35 | | 1.00 |
| | | '4042 | 8.19 | 0.03 | -0.09 | 0.03 | $t(3) = 3.77$, $P = 0.03$ | 0.81 |
| | | '1350 | 8.34 | 0.08 | 0.06 | 0.08 | $t(4) = 0.95$, $P = 0.4$ | 1.16 |
| hCB2 | $G_{i1}$ | CP-55,940 | 8.86 | 0.12 | 0.00 | 0.17 | | 1.00 |
| | | '4042 | 8.49 | 0.03 | -0.20 | 0.13 | $t(2) = 1.0$, $P = 0.4$ | 0.63 |
| | | '1350 | 8.32 | 0.10 | -0.54 | 0.16 | $t(2) = 5.3$, $P = 0.03$ | 0.29 |
| | $G_{i2}$ | CP-55,940 | 8.97 | 0.00 | 0.00 | 0.00 | | 1.00 |
| | | '4042 | 8.74 | 0.00 | -0.23 | 0.00 | [c]-- | 0.59 |
| | | '1350 | 8.57 | 0.00 | -0.40 | 0.00 | -- | 0.40 |
| | $G_{oB}$ | CP-55,940 | 8.76 | 0.09 | 0.00 | 0.13 | | 1.00 |
| | | '4042 | 8.43 | 0.19 | -0.33 | 0.21 | $t(2) = 1.7$, $P = 0.2$ | 0.47 |

| | | | | | | | | |
|---|---|---|---|---|---|---|---|---|
| | | '1350 | 8.36 | 0.01 | -0.40 | 0.09 | $t(2) = 33.7$, $P = 0.009$ | 0.40 |
| | $G_z$ | CP-55,940 | 8.90 | 0.39 | 0.00 | 0.55 | | 1.00 |
| | | '4042 | 8.48 | 0.51 | -0.42 | 0.64 | $t(2) = 0.8$, $P = 0.5$ | 0.38 |
| | | '1350 | 8.24 | 0.23 | -0.65 | 0.45 | $t(2) = 2.8$, $P = 0.1$ | 0.22 |
| | $ß_{arr2}$+ GRK2 | CP-55,940 | 7.83 | 0.13 | 0.00 | 0.18 | | 1.00 |
| | | '4042 | 7.74 | 0.00 | -0.09 | 0.13 | $t(2) = 43.7$, $P = 0.005$ | 0.82 |
| | | '1350 | 7.79 | 0.09 | -0.04 | 0.16 | $t(2) = 0.41$, $P = 0.7$ | 0.92 |

[a]Statistical significance of test compounds vs. CP-55,940 Mean Δlog ($E_{max}/EC_{50}$) by unpaired two-tailed t-test.
[b]RE, relative efficacy = $10^{\Delta \log (Emax/EC50)}$
[c]--, not determined

Supplementary Table 6. Binding affinities and functional activities for select active analogs at CB2.

| Compound | hCB2 binding<br>$EC_{50}$ [95% CI] (nM)<br>$pEC_{50}$ [95% CI]<br>$E_{max}$ [SEM] | hCB2<br>Cerep cAMP<br>$EC_{50}$ [95% CI] (nM)<br>$pEC_{50}$ [95% CI]<br>$E_{max}$ [95% CI] | hCB2<br>BRET2 + GoA<br>$EC_{50}$ [95% CI] (nM)<br>$pEC_{50}$ [95% CI]<br>$E_{max}$ [95% CI] | hCB2<br>Tango<br>ß-arrestin<br>recruitment<br>$EC_{50}$ [95% CI] (nM)<br>$pEC_{50}$ [95% CI]<br>$E_{max}$ [95% CI] |
|---|---|---|---|---|
| 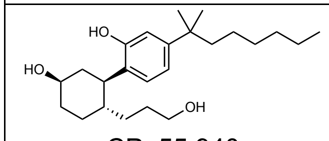<br>CP–55,940 | 4.3 [3.3 – 5.8]<br>8.4 [8.2 – 8.5]<br>100% [2.8] | -- | 13.1 [9.4 – 18.5]<br>7.9 [7.7 – 8.0]<br>99% [94 – 105] | 22.9 [21.3 – 24.8]<br>7.6 [7.60 – 7.67]<br>100% [98 – 102] |
| 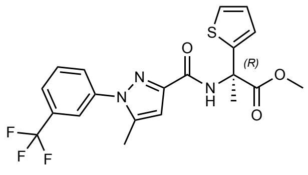<br>‘1350 | 2.9 [1.1 – 7.4]<br>8.5 [8.1 – 8.9]<br>73% [5.7] | | | |
| 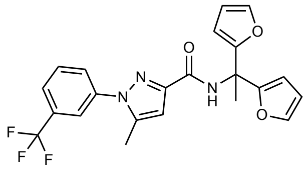<br>‘4936 | 6.7 [2.6 – 14.2]<br>8.2 [7.8– 8.6]<br>78% [5.7] | | | |
| 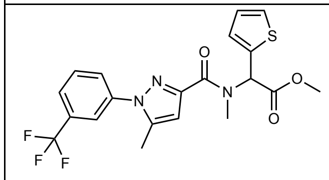<br>‘4042 | [b]-- | 0.011 [0.002 – 0.02]<br>10.95 [10.7 – 11.6]<br>72% [77 – 84] | 7.9 [1.6 – 40.6]<br>8.1 [7.4 – 8.8]<br>29% [22 – 36] | 33.9 [21.7 – 61.5]<br>7.5 [7.2 – 7.7]<br>28% [26 – 32] |

| | | | | |
|---|---|---|---|---|
| 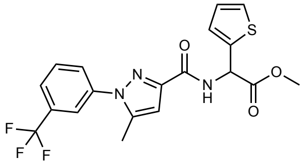<br>‘60154 | -- | -- | 594 [125 – 2813]<br>6.2 [5.6 – 6.9]<br>31% [20 – 42] | 3341 [2707 – 4371]<br>5.5 [5.4 – 5.6]<br>21% [19 – 23] |
| 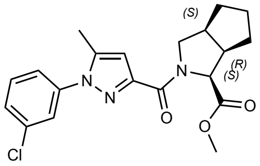<br>10010 | -- | -- | [a]N.D. | 556 [506 – 611]<br>6.3 [6.2 – 6.3]<br>18% [17 – 19] |
| 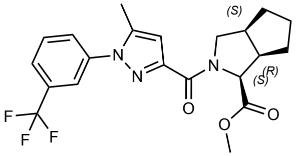<br>‘6439 | -- | -- | 97.1 [12.6 – 749]<br>7.0 [6.13 – 67.9]<br>81% [34 – 128] | 338 [282– 415]<br>6.5 [6.4 – 6.6]<br>14% [13 – 15] |
| 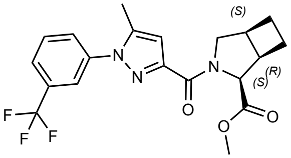<br>‘6448 | -- | -- | -- | -- |
| 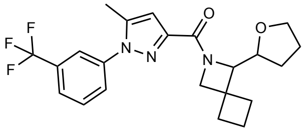<br>‘3737 | -- | 4.3 [.0004 – 22.6]<br>8.4 [7.7 – 12.5]<br>45% [42 – 80%] | N.D. | 1500 [1274 – 1805]<br>5.8 [5.7 – 5.9]<br>9% [8 – 9] |

[a]N.D. = best fit values were not determined due to compound inactivity or poor data quality
[b]-- Not tested

Supplementary Table 7. Functional activities for select analogs and controls versus a variety of transducers and hCB2 in the bioSensAll platform.

| Compound | | hCB2 $G_{i1}$ | hCB2 $G_{i2}$ | hCB2 $G_{oB}$ | hCB2 $G_z$ | hCB2 Barr2 + GRK2 |
|---|---|---|---|---|---|---|
| CP–55,940 | $EC_{50}$ [95% CI] (nM) | 1.4 [1.1 – 1.7] | 1.1 [0.9 – 1.3] | 1.7 [1.5 – 2.1] | 1.05 [0.6 – 1.9] | 14.3 [11.6 – 17.9] |
| | $pEC_{50}$ [95% CI] | 8.87 [8.7 – 8.9] | 8.97 [8.9 – 9.1] | 8.76 [8.7 – 8.8] | 8.98 [8.7 – 9.2] | 7.84 [7.8 – 7.9] |
| | $E_{max}$ [95% CI] | 100 [97 – 103] | 100 [97 – 104] | 100 [98 – 103] | 98 [89 – 109] | 100 [95 – 104] |
| ‘4042 | $EC_{50}$ [95% CI] (nM) | 2.7 [2.0 – 3.4] | 1.3 [0.9 – 1.7] | 2.5 [1.9 – 3.3] | 1.36 [0.5 – 3.1] | 5.5 [3.5 – 8.7] |
| | $pEC_{50}$ [95% CI] | 8.58 [8.5 – 8.7] | 8.90 [8.8 – 9.0] | 8.60 [8.5 – 8.7] | 8.87 [8.5 – 9.3] | 8.26 [8.1 – 8.5] |
| | $E_{max}$ [95% CI] | 82 [79 – 85] | 70 [67 – 73] | 61 [59 – 63] | 56 [47 – 68] | 33 [33 – 33] |
| ‘1350 | $EC_{50}$ [95% CI] (nM) | 3.55 [2.98 – 4.2] | 1.6 [1.4 – 1.9] | 2.6 [2.15 – 3.1] | 2.6 [1.6 – 3.9] | 4.2 [2.8 – 6.3] |
| | $pEC_{50}$ [95% CI] | 8.45 [8.4 – 8.5] | 8.79 [8.7 – 8.9] | 8.58 [8.5 – 8.7] | 8.58 [8.4 – 8.8] | 8.38 [8.2 – 8.6] |
| | $E_{max}$ [95% CI] | 74 [72 – 77] | 61 [59 –63] | 59 [57 – 61] | 52 [46 – 60] | 30 [28 –33] |

Supplementary Table 8. Cryo-EM data collection, model refinement, and validation statistics.

| **Data Collection** | **Global Refinement** | | |
|---|---|---|---|
| Voltage (kV) | 300 | | |
| Magnification | 96,000 | | |
| Total electron dose ($e^-/Å^2$) | 56.6 | | |
| Defocus range (μm) | -0.7 - -2.0 | | |
| Calibrated pixel size (Å) | 0.8521 | | |
| Micrographs collected | 8324 | | |
| **Data Processing** | | | |
| Extracted particles | 4,967,593 | | |
| Particles used for final reconstruction | 465,411 | | |
| Final map resolution (Å, 0.143 FSC) | 3.3 | | |
| Map resolution range (Å) | 2.6 - 4.2 | | |
| Map sharpening B factor ($Å^2$) | 175.3 | | |
| **Model Content** | | | |
| Initial models used (PDB code) | 6N4B (CB1/$G_i$/scFv16) | | |
| Total number of atoms | 8,528 | | |
| No. of protein residues | 1116 | | |
| No. of ligands | 1 | | |
| **Model Validation** | **CB1:$G_i$** | **CB1 alone** | **CB1:$G_i$ composite** |
| PDB. | 8GAG | 9DGI | 9EGO |
| EMDB | EMD-29898 | EMD-46828 | EMD-47992 |
| CC map vs. model (%) RMSD | 69.88 | 73.64 | 75.40 |
| Bond lengths (Å) / Bond angles (°) | 0.006 / 0.829 | 0.005/1.265 | 0.007/1.029 |
| Ramachandran plot statistics | | | |
| Favored (%) | 87.86 | 89.49 | 93.93 |
| Allowed (%) | 12.14 | 10.12 | 5.97 |
| Outliers (%) | 0.0 | 0.39 | 0.09 |
| Rotamer outliers (%) | 0.0 | 0.99 | 0.34 |
| C-beta deviations | 0.0 | 0.0 | 0.0 |
| Clash score | 10.08 | 1.47 | 2.79 |

Supplementary Table 9. Aqueous solubility for lead compound ‘1350.

| Compound | PBS solubility, pH 7.4, µM | | | |
|---|---|---|---|---|
| | Rep. 1 | Rep. 2 | Mean | SE |
| Ondansetron | 121 | 119 | 120 | 0.7 |
| ‘1350 | 23 | 24 | 23 | 0.3 |

Supplementary Table 10. Mouse plasma protein binding for lead compound ‘1350.

| Compound | Type of plasma | Conc, μM[a] | Rep.[b] | Area Ratio | | % of bound compound | Mean % of bound compound | Recovery % | Stability % |
|---|---|---|---|---|---|---|---|---|---|
| | | | | Buffer | Plasma | | | | |
| Verapamil | Mouse | 1.0 | 1 | 3.51E-02 | 2.87E-01 | 88 | 88 | 91 | 106 |
| | | | 2 | 3.54E-02 | 2.85E-01 | 88 | | | |
| ‘1350 | Mouse | 1.0 | 1 | 8.97E-04 | 1.56E-02 | 94 | 94* | 107 | 11 |
| | | | 2 | 9.36E-04 | 1.69E-02 | 94 | | | |

[a]Concentration
[b]Replicate number
*Parameter should be considered as approximate, compound show low stability in mouse plasma

Supplementary Table 11. Mouse plasma stability for lead compound ‘1350.

| Compound | Time, min | Area Ratio | | Mean Area Ratio | Mean % Remaining | $T_{1/2}$, min |
|---|---|---|---|---|---|---|
| | | Rep. 1 | Rep. 2 | | | |
| Verapamil | 0 | 7.05E-01 | 6.50E-01 | 6.77E-01 | 100 | 1263* |
| | 20 | 6.86E-01 | 6.39E-01 | 6.63E-01 | 98 | |
| | 40 | 7.12E-01 | 6.59E-01 | 6.85E-01 | 101 | |
| | 60 | 7.20E-01 | 6.60E-01 | 6.90E-01 | 102 | |
| | 120 | 6.24E-01 | 6.33E-01 | 6.29E-01 | 93 | |
| Propantheline | 0 | 1.09E+00 | 1.01E+00 | 1.05E+00 | 100 | 28 |
| | 20 | 8.21E-01 | 7.61E-01 | 7.91E-01 | 75 | |
| | 40 | 5.51E-01 | 4.93E-01 | 5.22E-01 | 50 | |
| | 60 | 3.34E-01 | 3.09E-01 | 3.22E-01 | 31 | |
| | 120 | 6.04E-02 | 5.75E-02 | 5.90E-02 | 6 | |
| ‘1350 | 0 | 2.86E-01 | 2.61E-01 | 2.73E-01 | 100 | 41 |
| | 20 | 2.37E-01 | 2.23E-01 | 2.30E-01 | 84 | |
| | 40 | 1.86E-01 | 1.77E-01 | 1.81E-01 | 66 | |
| | 60 | 1.24E-01 | 1.37E-01 | 1.31E-01 | 48 | |
| | 120 | 3.73E-02 | 3.73E-02 | 3.73E-02 | 14 | |

*Parameter should be considered as approximate due to the high stability of the compound

Supplementary Table 12. Mouse microsomal stability for lead compound 1350.

| Compound | Time, min | Analyte Peak Area | | Mean Analyte Peak Area | Mean % Remaining | $R^2$ | $k_{el}$, $min^{-1}$ | $t_{1/2}$, min | $Cl_{int}$, µL/min/mg | Mean % Remaining without cofactor |
|---|---|---|---|---|---|---|---|---|---|---|
| | | Rep. 1 | Rep. 2 | | | | | | | |
| Propranolol | 0 | 1.53E-01 | 1.50E-01 | 1.51E-01 | 100 | 0.894 | 0.041 | 16.9 | 99 | 100 |
| | 7 | 7.43E-02 | 6.79E-02 | 7.11E-02 | 47 | | | | | |
| | 15 | 5.08E-02 | 4.64E-02 | 4.86E-02 | 32 | | | | | |
| | 25 | 3.83E-02 | 3.61E-02 | 3.72E-02 | 25 | | | | | |
| | 40 | 2.70E-02 | 2.36E-02 | 2.53E-02 | 17 | | 106 | | | |
| Imipramine | 0 | 1.40E+00 | 1.41E+00 | 1.40E+00 | 100 | 0.997 | 0.135 | 5.1 | 325 | 100 |
| | 7 | 7.04E-01 | 6.98E-01 | 7.01E-01 | 50 | | | | | |
| | 15 | 2.20E-01 | 2.67E-01 | 2.43E-01 | 17 | | | | | |
| | 25 | 5.44E-02 | 6.58E-02 | 6.01E-02 | 4 | | | | | |
| | 40 | 6.38E-03 | 7.22E-03 | 6.80E-03 | 0 | | 104 | | | |
| ‘1350 | 0 | 9.08E-01 | 9.27E-01 | 9.17E-01 | 100 | 0.990 | 0.360* | 1.9* | 868* | 100 |
| | 7 | 9.72E-02 | 1.42E-01 | 1.20E-01 | 13 | | | | | |
| | 15 | 3.31E-03 | 5.15E-03 | 4.23E-03 | 0 | | | | | |
| | 25 | 1.28E-03 | 1.67E-03 | 1.48E-03 | 0 | | | | | |
| | 40 | 1.42E-03 | 9.18E-04 | 1.17E-03 | 0 | | 100 | | | |

*Due to rapid degradation of compound, the metabolic stability parameters are considered ambigous

Supplementary Table 13. MPO scores for lead compounds and CB1 controls.

| Compound | Structure | Physicochemical properties | | | | | | MPO Score |
|---|---|---|---|---|---|---|---|---|
| | | MW[a] (g/mol) | clogP[b] | tPSA[c] | pKa[d] | clogD$_{7.4}$[e] | HBD[f] | |
| '1350 | 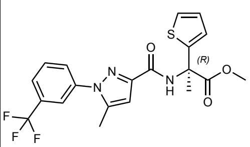 | 437 | 4.08 | 73.22 | 0.07 | 4.50 | 1 | 3.7 |
| '4936 | 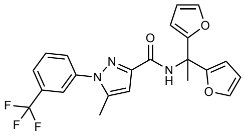 | 429 | 5.08 | 73.20 | 0.08 | 4.37 | 1 | 3.3 |
| CP-55,940 | 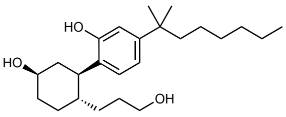 | 377 | 5.66 | 60.69 | -2.02 | 5.90 | 3 | 3.0 |
| WIN 55-212,2 | 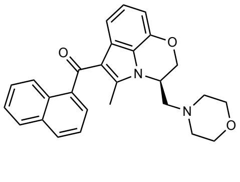 | 427 | 4.60 | 43.70 | 6.93 | 4.35 | 0 | 3.7 |
| MDMB-Fub | 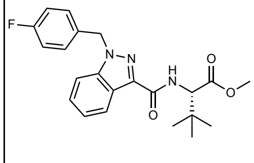 | 397 | 3.54 | 73.22 | -0.85 | 4.21 | 1 | 4.3 |
| Rimonabant | 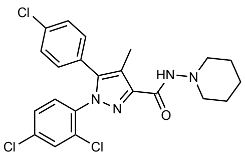 | 464 | 5.94 | 50.16 | 1.68 | 5.91 | 1 | 3.1 |

[a]MW = Molecular Weight in g/mol
[b]cLogP = calculated LogP
[c]tPSA = Topological Polar Surface Area
[d]pKa = negative log of the acid dissociation constant
[e]cLogD$_{7.4}$ = calculated log distribution coefficient at pH 7.4
[f]HBD = Hydrogen bond donors

## Supplementary Methods

### Synthetic procedures.

**Method 1.** A carboxylic acid (100 mg), 0.7 ml of acetonitrile, a hydrazide (1.1 mol. eq. to the acid), and triethylamine (TEA, 1.1 mol. eq. to the hydrazide) were placed into a 4 ml capped glass vial and the mixture was stirred for 30 min at RT. Then, chloro-N,N,N′,N′-tetramethylformamidinium hexafluorophosphate (1.2 mol. eq. to the acid) was added. If the solution was transparent, the mixture was left for 48 hours at RT as is; otherwise, the vial was placed in the ultrasonic bath and left for 2-3 hours. Then, the mixture was heated in an oven for 6 hours at 100$^{o}$C. The solvent and volatile components were evaporated under reduced pressure to give the crude product. The product was further purified by HPLC.

**Method 2.** A carboxylic acid (100 mg), 0.6 ml of DMSO, alcohol (1.1 mol. eq. to the acid), TEA (1.1 mol. eq. to the amine if it is in the form of hydrochloride), and 4-dimethylaminopyridine (DMAP, catalytic amount) were placed into a 4 ml capped glass vial and the mixture was stirred for 15 min at RT. Then, 1,1'-carbonyldiimidazole (CDI, 1.2 mol. eq. to the acid) was added. If the solution was transparent, the mixture was left for 72 hours at RT as is; otherwise, the vial was placed in the ultrasonic bath and left for 2-3 hours. After the reaction is completed, 40 uL of formic acid was added and the mixture was shaken for 15 min at RT. The solvent and volatile components were evaporated under reduced pressure to give the crude product. The product was further purified by HPLC.

**Method 3.** An amine (100 mg), a carboxylic acid (1.1 mol. eq. to the amine), TEA (1.1 mol. eq. to the amine if it is in the form of hydrochloride), and 0.5 ml of DMSO were placed into a 4 ml capped glass vial and the mixture was stirred for 30 min at RT. Then, 1-ethyl-3-(3-dimethylaminopropyl)carbodiimide (EDC, 1.2 mol. eq. to the amine) was added and the mixture was stirred for 1 hour. If the solution was transparent, the mixture was left overnight at room temperature as is; otherwise, the vial was placed in the ultrasonic bath and left overnight. The solution was filtered, and the solvent and volatile components were evaporated under reduced pressure to give the crude product. The product was further purified by HPLC.

**Method 4.** A thiourea (100 mg), 0.7 ml of acetonitrile, and an α-halo ketone (1.1 mol. eq. to the acid) were placed into a 4 ml capped glass vial and the mixture was shaken for 30 min at RT. Then, the mixture was heated in an oven for 4 hours at 100$^{o}$C. The solution was cooled down to RT and 0.2 ml of TEA, C18 molecular sieves, and 2 ml of acetonitrile were added. The mixture was shaken for 15 min at RT and the sieves were filtered off. The solvent and volatile components were evaporated under reduced pressure to give the crude product. The product was further purified by HPLC.

**Method 5.** The previously published procedure was utilized.[80]

**Method 6.** An azide (100 mg), an alkyne (1 mol. eq. to the azide), and 0.5 ml of dioxane were placed into a 4 ml capped glass vial and the mixture was stirred for 30 min at RT. Then, the sodium ascorbate (0.1 mol. eq. to the azide) and copper acetate (0.25 mol-% to the azide) were added and the mixture was heated in the oven for 6 hours at 60$^{o}$C. After completion of the reaction, 2 mL of methanol was added followed by C18 sieves. The vial was shaken for 30 min at RT and the sieves were filtered off. The filtrate was evaporated to give a crude product. The product was further purified by HPLC.

**Spectral Description.**

**ethyl 1-(1-(3-chlorophenyl)-5-methyl-1H-pyrazole-3-carboxamido)cyclohexane-1-carboxylate – '64801, PV-001795007500 (Method 3).**

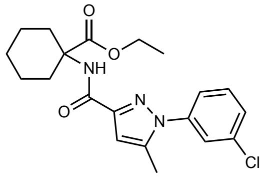

The compound was synthesized from ethyl 1-aminocyclohexane-1-carboxylate hydrochloride (Catalog # EN300-43194, 100 mg, 0.48 mmol) and 1-(3-chlorophenyl)-5-methyl-1H-pyrazole-3-carboxylic acid (Catalog # EN300-127105, 125 mg, 0.53 mmol).
Yield: 55%; purity, >95% (assessed by LC/MS).
$^1$H NMR (500 MHz, DMSO-$d_6$) δ 7.82 (s, 1H), 7.78 (t, $J$ = 2.0 Hz, 1H), 7.64 – 7.51 (m, 2H), 6.64 (d, $J$ = 1.0 Hz, 1H), 4.04 (q, $J$ = 7.1 Hz, 1H), 2.35 (t, $J$ = 2.0 Hz, 3H), 2.08 – 1.98 (m, 2H), 1.78 (ddd, $J$ = 13.8, 10.1, 3.8 Hz, 2H), 1.55 – 1.42 (m, 6H), 1.29 (d, $J$ = 11.6 Hz, 1H), 1.11 (t, $J$ = 7.1 Hz, 2H).
$^{13}$C NMR (151 MHz, DMSO-$d_6$) δ 173.9, 161.3, 147.1, 141.6, 140.6, 134.0, 131.3, 128.6, 125.2, 123.9, 108.0, 60.6, 58.5, 32.5, 25.3, 21.7, 14.5, 12.5.
LC/MS (APSI) m/z [M+H] calculated for $C_{20}H_{25}ClN_3O_3$: 390.2; found: 390.1.

**methyl 2-(5-methyl-1-(3-(trifluoromethyl)phenyl)-1H-pyrazole-3-carboxamido)-2-(thiophen-2-yl)acetate – '60154, PV-001796383931 (Method 3).**

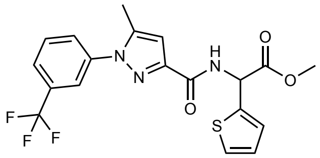

The compound was synthesized from methyl 2-amino-2-(thiophen-2-yl)acetate hydrochloride (Catalog # EN300-60031, 100 mg, 0.48 mmol) and 5-methyl-1-[3-(trifluoromethyl)phenyl]-1H-pyrazole-3-carboxylic acid (Catalog # EN300-260239, 143 mg, 0.53 mmol).
Yield: 66%; purity, >95% (assessed by LC/MS).
$^1$H NMR (500 MHz, DMSO-$d_6$) δ 8.90 (d, $J$ = 7.5 Hz, 1H), 8.00 (d, $J$ = 2.1 Hz, 1H), 7.98 – 7.92 (m, 1H), 7.88 – 7.83 (m, 1H), 7.79 (t, $J$ = 7.9 Hz, 1H), 7.48 (dt, $J$ = 5.1, 1.1 Hz, 1H), 7.15 (dd, $J$ = 3.5, 1.0 Hz, 1H), 7.02 – 6.96 (m, 1H), 6.75 (d, $J$ = 1.0 Hz, 1H), 5.87 (d, $J$ = 7.5 Hz, 1H), 3.68 (s, 3H), 2.37 (s, 3H).
$^{13}$C NMR (151 MHz, DMSO-$d_6$) δ 170.4, 163.4, 161.3, 147.8, 146.6, 141.9, 141.6, 139.9, 139.5, 139.0, 138.4, 131.1, 131.0, 130.7, 130.5, 129.8, 129.2, 128.9, 127.6, 127.1, 126.9, 125.4, 125.4, 125.0, 123.2, 122.1, 122.1, 122.0, 121.9, 108.3, 108.1, 53.6, 53.0, 51.9, 12.6, 12.5.
LC/MS (APSI) m/z [M+H] calculated for $C_{19}H_{17}F_3N_3O_3S$: 424.1; found: 424.2.

**methyl 2-(1-(3-chlorophenyl)-5-methyl-1H-pyrazole-3-carboxamido)-2-(4-(trifluoromethyl)phenyl)acetate – '14083, PV-001818789715 (Method 3).**

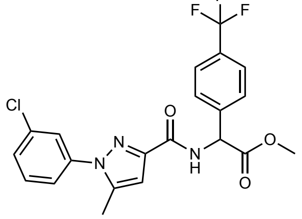

The compound was synthesized from methyl 2-amino-2-[4-(trifluoromethyl)phenyl]acetate hydrochloride (Catalog # EN300-316396, 100 mg, 037 mmol) and 1-(3-chlorophenyl)-5-methyl-1H-pyrazole-3-carboxylic acid (Catalog # EN300-127105, 97 mg, 0.41 mmol).
Yield: 59%; purity, >95% (assessed by LC/MS).
$^{1}$H NMR (500 MHz, DMSO-$d_6$) δ 8.99 (d, $J$ = 7.5 Hz, 1H), 7.78 – 7.70 (m, 3H), 7.68 (d, $J$ = 7.9 Hz, 2H), 7.64 – 7.52 (m, 3H), 6.69 (s, 1H), 5.81 (d, $J$ = 7.5 Hz, 1H), 3.66 (s, 2H), 2.35 (s, 3H).
$^{13}$C NMR (151 MHz, DMSO-$d_6$) δ 170.7, 161.4, 146.4, 142.0, 141.8, 140.5, 134.0, 131.4, 129.5, 129.1, 128.9, 128.8, 125.8, 125.8, 125.7, 125.5, 125.3, 123.9, 123.7, 108.1, 55.9, 53.1, 12.50.
LC/MS (APSI) m/z [M+H] calculated for $C_{21}H_{18}ClF_3N_3O_3$: 452.1; found: 452.0.

**(2,3-dihydrobenzo[b][1,4]oxathiin-2-yl)methyl 4-((1H-pyrrol-1-yl)methyl)benzoate – '30177, Z1124220507 (Method 2).**

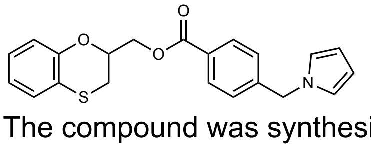

The compound was synthesized from (2,3-dihydro-1,4-benzoxathiin-2-yl)methanol (Catalog # EN300-59707, 100 mg, 0.55 mmol ) and 4-[(1H-pyrrol-1-yl)methyl]benzoic acid (Catalog # EN300-69560, 121 mg, 0.6 mmol).
Yield: 26%; purity, >95% (assessed by LC/MS).
$^{1}$H NMR (500 MHz, DMSO-$d_6$) δ 7.97 – 7.90 (m, 2H), 7.32 (dd, $J$ = 7.6, 1.7 Hz, 1H), 7.27 (d, $J$ = 8.0 Hz, 2H), 7.18 (td, $J$ = 7.7, 1.7 Hz, 1H), 7.00 (t, $J$ = 7.8 Hz, 2H), 6.80 (q, $J$ = 4.2, 3.2 Hz, 2H), 6.02 (t, $J$ = 2.1 Hz, 2H), 5.44 – 5.36 (m, 1H), 5.19 (s, 2H), 4.52 (dd, $J$ = 13.3, 3.1 Hz, 1H), 4.29 (dd, $J$ = 13.3, 4.3 Hz, 1H), 3.31 – 3.22 (m, 2H).
$^{13}$C NMR (151 MHz, DMSO-$d_6$) δ 165.1, 159.3, 145.4, 131.6, 130.1, 128.9, 128.7, 127.7, 126.7, 124.2, 122.3, 121.5, 108.7, 73.0, 73.0, 52.2, 33.4.
LC/MS (APSI) m/z [M+H] calculated for $C_{21}H_{20}NO_3S$: 366.1; found: 366.0.

**(2,3-dihydrobenzo[b][1,4]oxathiin-2-yl)methyl 4-phenoxypicolinate – '50267, Z1309468381 (Method 2).**

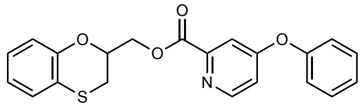

The compound was synthesized from (2,3-dihydro-1,4-benzoxathiin-2-yl)methanol (Catalog # EN300-59707, 100 mg, 0.55 mmol) and 4-phenoxypyridine-2-carboxylic acid hydrochloride (Catalog # EN300-98150, 151 mg, 0.6 mmol).
Yield: 48%; purity, >95% (assessed by LC/MS).
$^{1}$H NMR (500 MHz, DMSO-$d_6$) δ 8.60 (d, $J$ = 5.5 Hz, 1H), 7.55 – 7.46 (m, 3H), 7.36 – 7.28 (m, 2H), 7.25 – 7.13 (m, 4H), 7.03 – 6.95 (m, 2H), 5.42 (dtd, $J$ = 7.9, 4.7, 3.1 Hz, 1H), 4.55 (dd, $J$ = 13.2, 3.2 Hz, 1H), 4.27 (dd, $J$ = 13.2, 4.4 Hz, 1H), 3.35 – 3.23 (m, 2H).
$^{13}$C NMR (151 MHz, DMSO-$d_6$) δ 165.5, 164.0, 159.1, 153.7, 152.2, 149.9, 131.6, 131.1, 128.7, 126.5, 126.4, 124.1, 122.2, 121.2, 115.6, 113.6, 73.8, 72.8, 33.2.
LC/MS (APSI) m/z [M+H] calculated for $C_{21}H_{18}NO_4S$: 380.1; found: 380.0.

**(2,3-dihydrobenzo[b][1,4]oxathiin-2-yl)methyl 1-(3-fluorobenzyl)-1H-1,2,3-triazole-4-carboxylate – '23346, Z1415595403 (Method 2).**

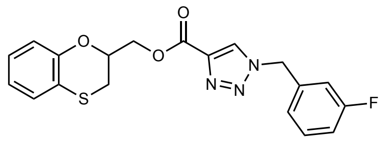

The compound was synthesized from (2,3-dihydro-1,4-benzoxathiin-2-yl)methanol (Catalog # EN300-59707, 100 mg, 0.55 mmol) and 1-[(3-fluorophenyl)methyl]-1H-1,2,3-triazole-4-carboxylic acid (Catalog # EN300-106975, 133 mg, 0.6 mmol).
Yield: 47%; purity, >95% (assessed by LC/MS).
$^1$H NMR (500 MHz, DMSO-$d_6$) δ 7.42 (td, $J$ = 8.0, 6.0 Hz, 1H), 7.33 – 7.26 (m, 1H), 7.22 (dt, $J$ = 9.7, 2.2 Hz, 1H), 7.17 (dq, $J$ = 9.2, 3.4, 2.7 Hz, 3H), 7.02 – 6.96 (m, 2H), 5.69 (s, 2H), 5.43 (dq, $J$ = 8.2, 4.0 Hz, 1H), 4.56 (dd, $J$ = 13.3, 3.2 Hz, 1H), 4.27 (dd, $J$ = 13.3, 4.3 Hz, 1H), 3.32 – 3.23 (m, 2H).
$^{13}$C NMR (151 MHz, DMSO-$d_6$) δ 163.4, 161.8, 159.7, 159.0, 139.1, 138.5, 138.5, 131.5, 131.4, 131.4, 130.2, 128.7, 126.4, 124.7, 124.7, 124.1, 122.2, 115.8, 115.6, 115.6, 115.4, 73.0, 72.9, 52.9, 33.2.
LC/MS (APSI) m/z [M+H] calculated for $C_{19}H_{17}FN_3O3S$: 386.1; found: 386.0.

**8-(2-(4-fluorobenzyl)thiazol-4-yl)-5,6-dihydro-1H-pyrrolo[3,2,1-ij]quinolin-2(4H)-one – '1038, Z371852066 (Method 4).**

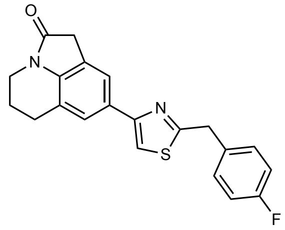


The compound was synthesized from 2-(4-fluorophenyl)ethanethioamide (Catalog # EN300-36372, 100 mg, 0.6 mmol) and 6-(2-chloroacetyl)-1-azatricyclo[6.3.1.0,4,12]dodeca-4(12),5,7-trien-2-one (Catalog # EN300-13775, 162 mg, 0.65 mmol).
Yield: 22%; purity, >95% (assessed by LC/MS).
$^1$H NMR (500 MHz, DMSO-$d_6$) δ 7.74 (s, 1H), 7.68 – 7.63 (m, 2H), 7.40 (ddd, $J$ = 9.5, 6.0, 3.0 Hz, 2H), 7.21 – 7.11 (m, 2H), 4.34 (s, 2H), 3.59 (t, $J$ = 5.8 Hz, 2H), 3.54 (s, 2H), 2.75 (t, $J$ = 6.0 Hz, 2H), 1.91 (p, $J$ = 5.9 Hz, 2H).
$^{13}$C NMR (151 MHz, DMSO-$d_6$) δ 173.7, 155.2, 141.4, 134.8, 131.4, 131.3, 128.3, 124.8, 124.1, 120.5, 120.2, 115.9, 115.8, 112.2, 38.8, 38.2, 36.4, 24.3, 21.3.
LC/MS (APSI) m/z [M+H] calculated for $C_{21}H_{18}FN_2OS$: 365.1; found: 365.2.

**(2,3-dihydrobenzo[b][1,4]dioxin-2-yl)methyl 1-benzyl-5-methyl-1H-pyrazole-4-carboxylate – '32445, Z419297242 (Method 2).**

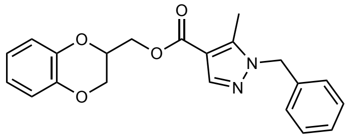


The compound was synthesized from (2,3-dihydro-1,4-benzodioxin-2-yl)methanol (Catalog # EN300-03215, 100 mg, 0.6 mmol) and 1-benzyl-5-methyl-1H-pyrazole-4-carboxylic acid (Catalog # EN300-40120, 143 mmol, 0.66 mmol).
Yield: 30%; purity, >95% (assessed by LC/MS).
$^1$H NMR (500 MHz, DMSO-$d_6$) δ 7.85 (s, 1H), 7.32 (dd, $J$ = 8.2, 6.6 Hz, 2H), 7.31 – 7.23 (m, 1H), 7.14 – 7.09 (m, 2H), 6.91 – 6.77 (m, 4H), 5.36 (s, 2H), 4.50 (ddt, $J$ = 7.0, 4.4, 2.7 Hz, 1H), 4.47 – 4.34 (m, 3H), 4.11 (dd, $J$ = 11.6, 7.0 Hz, 1H), 2.45 (s, 3H).
$^{13}$C NMR (151 MHz, DMSO-$d_6$) δ 163.0, 143.8, 143.3, 143.2, 141.0, 136.9, 129.1, 128.1, 127.5, 122.0, 121.8, 117.6, 117.4, 111.2, 71.3, 65.1, 62.3, 52.7, 10.7.
LC/MS (APSI) m/z [M+H] calculated for $C_{21}H_{21}N_2O_4$: 365.1; found: 365.2.

**rac-methyl 2-(N,5-dimethyl-1-(3-(trifluoromethyl)phenyl)-1H-pyrazole-3-carboxamido)-2-(thiophen-2-yl)acetate – ‘3234, Z4971163234 (Method 3).**

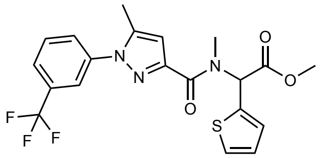

The compound was synthesized from methyl 2-(methylamino)-2-(thiophen-2-yl)acetate hydrochloride (Catalog # EN300-60031, 100 mg, 0.48 mmol) and 5-methyl-1-[3-(trifluoromethyl)phenyl]-1H-pyrazole-3-carboxylic acid (Catalog # EN300-260239, 143 mg,0.53 mmol).

Yield: 68%; purity, >95% (assessed by LC/MS).

$^1$H NMR (500 MHz, DMSO-$d_6$) δ 8.68 (s, 1H), 7.99 (d, *J* = 2.0 Hz, 1H), 7.95 (dd, *J* = 7.9, 2.2 Hz, 1H), 7.89 – 7.83 (m, 1H), 7.79 (t, *J* = 7.9 Hz, 1H), 7.45 (dd, *J* = 5.1, 1.3 Hz, 1H), 7.14 (dd, *J* = 3.7, 1.3 Hz, 1H), 6.96 (dd, *J* = 5.1, 3.7 Hz, 1H), 6.71 (d, *J* = 0.9 Hz, 1H), 3.62 (s, 3H), 2.36 (s, 3H), 1.91 (s, 3H).

$^{13}$C NMR (151 MHz, DMSO-$d_6$) δ 172.3, 161.0, 146.8, 145.2, 142.0, 139.9, 131.1, 130.7, 130.5, 129.3, 126.9, 126.4, 126.0, 125.5, 125.4, 125.0, 123.2, 122.2, 122.2, 122.2, 108.2, 59.5, 53.1, 25.7, 12.5.

LC/MS (APSI) m/z [M-H] calculated for $C_{20}H_{17}F_3N_3O_3S$: 436.1; found: 436.0.

***rel*-methyl (S)-2-(N,5-dimethyl-1-(3-(trifluoromethyl)phenyl)-1H-pyrazole-3-carboxamido)-2-(thiophen-2-yl)acetate – ‘6000, Z5199560000 (Method 3).**

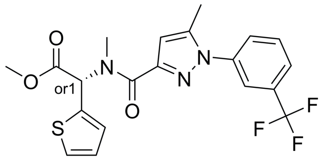

The compound was obtained by a chiral separation of Z4971163234.

Yield: 20%; purity, >95% (assessed by LC/MS).

$[\alpha]^{21}_D$ = 57.7 (*c* 2.0, $CH_3OH$).

$^1$H NMR (400 MHz, DMSO-$d_6$) δ 7.94 (s, 2H), 7.86 – 7.77 (m, 2H), 7.58 (s, 1H), 7.14 (d, *J* = 17.0 Hz, 1H), 7.04 (s, 1H), 6.72 (d, *J* = 16.4 Hz, 1H), 6.22 (s, 1H), 3.70 (d, *J* = 6.6 Hz, 3H), 3.25 (s, 2H), 2.83 (s, 1H), 2.42 (s, 1H), 2.38 (s, 2H).

LC/MS (APSI) m/z [M+H] calculated for $C_{20}H_{19}F_3N_3O3S$: 438.1; found: 438.2.

***rel*-methyl (R)-2-(N,5-dimethyl-1-(3-(trifluoromethyl)phenyl)-1H-pyrazole-3-carboxamido)-2-(thiophen-2-yl)acetate – ‘1066, Z5199561066 (Method 3).**

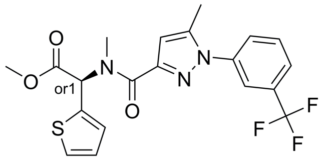

The compound was obtained by a chiral separation of Z4971163234.

Yield: 23%; purity, >95% (assessed by LC/MS).

$[\alpha]^{21}_D$ = -58.6 (*c* 2.0, $CH_3OH$).

$^1$H NMR (400 MHz, Chloroform-*d*) δ 7.84 (s, 1H), 7.73 (s, 1H), 7.71 – 7.58 (m, 3H), 7.25 (dd, *J* = 5.2, 1.3 Hz, 1H), 7.16 (dd, *J* = 3.6, 1.3 Hz, 1H), 6.96 (dd, *J* = 5.2, 3.6 Hz, 1H), 6.72 (s, 1H), 3.76 (s, 3H), 2.35 (s, 3H), 2.16 (s, 3H).

LC/MS (APSI) m/z [M+H] calculated for $C_{20}H_{19}F_3N_3O_3S$: 438.1; found: 438.2.

**4-(5-(2-(4-chlorophenoxy)propan-2-yl)-1,3,4-oxadiazol-2-yl)-2-methylphthalazin-1(2H)-one – ‘0450, Z685931568 (Method 1).**

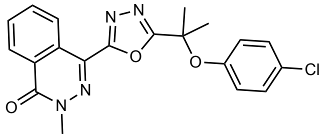

The compound was synthesized from 3-methyl-4-oxo-3,4-dihydrophthalazine-1-carbohydrazide (Catalog # EN300-04897, 112 mg. 0.51 mmol) and 2-(4-chlorophenoxy)-2-methylpropanoic acid (Catalog # EN300-18267, 100 mg, 0.47 mmol).
Yield: 24%; purity, >95% (assessed by LC/MS).
$^{1}$H NMR (400 MHz, DMSO-$d_6$) δ 8.93 (d, $J$ = 8.2 Hz, 1H), 8.40 (d, $J$ = 7.8 Hz, 1H), 8.09 (t, $J$ = 7.7 Hz, 1H), 7.99 (t, $J$ = 7.6 Hz, 1H), 7.35 – 7.28 (m, 2H), 6.87 – 6.81 (m, 2H), 3.87 (s, 3H), 1.84 (s, 6H).
$^{13}$C NMR (151 MHz, DMSO-$d_6$) δ 168.1, 161.8, 158.8, 153.4, 134.6, 133.0, 130.2, 129.9, 129.8, 128.5, 127.4, 127.3, 126.9, 126.6, 124.1, 123.4, 76.0, 26.0.
LC/MS (APSI) m/z [M+H] calculated for $C_{20}H_{18}ClN_4O_3$: 397.1; found: 397.2.

**(2,3-dihydrobenzo[b][1,4]oxathiin-2-yl)methyl 4-(pyridin-3-yloxy)benzoate – ‘82778, Z975741758 (Method 2).**

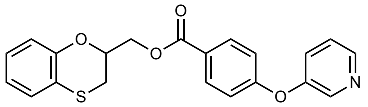

The compound was synthesized from (2,3-dihydro-1,4-benzoxathiin-2-yl)methanol (Catalog # EN300-59707, 100 mg, 0.55 mmol) and 4-(pyridin-3-yloxy)benzoic acid (Catalog # EN300-54901, 130 mg, 0.61 mmol).
Yield: 39%; purity, >95% (assessed by LC/MS).
$^{1}$H NMR (500 MHz, DMSO-$d_6$) δ 8.48 – 8.41 (m, 2H), 8.06 – 7.98 (m, 2H), 7.58 (ddd, $J$ = 8.3, 2.8, 1.4 Hz, 1H), 7.51 – 7.44 (m, 1H), 7.33 (dd, $J$ = 7.7, 1.7 Hz, 1H), 7.19 (ddd, $J$ = 8.7, 7.3, 1.7 Hz, 1H), 7.17 – 7.10 (m, 2H), 7.05 – 6.97 (m, 2H), 5.41 (dtd, $J$ = 7.6, 4.5, 3.1 Hz, 1H), 4.52 (dd, $J$ = 13.3, 3.1 Hz, 1H), 4.30 (dd, $J$ = 13.2, 4.3 Hz, 1H), 3.35 – 3.23 (m, 2H).
$^{13}$C NMR (151 MHz, DMSO-$d_6$) δ 164.7, 161.3, 159.3, 152.3, 146.3, 142.4, 132.4, 131.6, 128.8, 127.7, 126.8, 125.4, 125.0, 124.2, 122.3, 118.2, 73.1, 72.9, 33.5.
LC/MS (APSI) m/z [M+H] calculated for $C_{21}H_{18}NO_4S$: 380.1; found: 380.0.

**(2,3-dihydrobenzo[b][1,4]oxathiin-2-yl)methyl 2-benzylthiazole-4-carboxylate – ‘86798, Z975742428 (Method 2).**

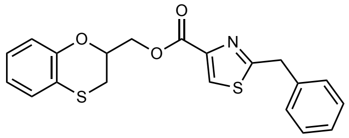

The compound was synthesized from (2,3-dihydro-1,4-benzoxathiin-2-yl)methanol (Catalog # EN300-59707, 100 mg, 0.55 mmol) and 2-benzyl-1,3-thiazole-4-carboxylic acid (Catalog # EN300-38897, 134 mg, 0.61 mmol).
Yield: 32%; purity, >95% (assessed by LC/MS).
$^{1}$H NMR (500 MHz, DMSO-$d_6$) δ 8.43 (d, $J$ = 1.6 Hz, 1H), 7.38 – 7.23 (m, 6H), 7.21 – 7.13 (m, 1H), 6.99 (td, $J$ = 7.3, 6.9, 1.3 Hz, 2H), 5.44 – 5.36 (m, 1H), 4.57 (dd, $J$ = 13.2, 3.2 Hz, 1H), 4.38 (s, 1H), 4.26 (dd, $J$ = 13.2, 4.3 Hz, 1H), 3.33 – 3.23 (m, 2H).
$^{13}$C NMR (151 MHz, DMSO-$d_6$) δ 171.6, 160.3, 159.0, 145.7, 138.2, 131.5, 130.7, 129.5, 129.2, 128.7, 127.6, 126.4, 124.1, 122.2, 73.2, 73.0, 38.9, 33.2.
LC/MS (APSI) m/z [M+H] calculated for $C_{20}H_{18}NO_3S_2$: 384.1; found: 384.1.

**(2,3-dihydrobenzo[b][1,4]oxathiin-2-yl)methyl 1-benzyl-5-methyl-1H-pyrazole-4-carboxylate – '7800, Z975742882 (Method 2).**

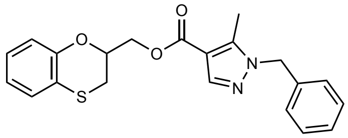

The compound was synthesized from (2,3-dihydro-1,4-benzoxathiin-2-yl)methanol (Catalog # EN300-59707, 100 mg, 0.55 mmol) and 1-benzyl-5-methyl-1H-pyrazole-4-carboxylic acid (Catalog # EN300-40120, 132 mg,0.61 mmol).
Yield: 33%; purity, >95% (assessed by LC/MS).
$^1$H NMR (500 MHz, DMSO-$d_6$) δ 7.86 (s, 1H), 7.36 – 7.23 (m, 5H), 7.18 (ddd, *J* = 8.6, 7.3, 1.7 Hz, 1H), 7.15 – 7.09 (m, 2H), 7.00 (t, *J* = 7.6 Hz, 2H), 5.35 (d, *J* = 19.3 Hz, 3H), 4.46 (dd, *J* = 13.2, 3.1 Hz, 1H), 4.24 (dd, *J* = 13.2, 4.5 Hz, 1H), 3.31 – 3.16 (m, 2H).
$^{13}$C NMR (126 MHz, Chloroform-*d*) δ 161.9, 158.9, 158.5, 143.3, 140.5, 136.4, 131.1, 128.6, 128.2, 127.6, 127.0, 126.4, 123.7, 121.8, 111.0, 72.7, 71.2, 52.3, 38.9, 33.1, 10.4.
LC/MS (APSI) m/z [M+H] calculated for $C_{21}H_{21}N_2O3S$: 381.1; found: 381.2.

**methyl 2-(2-benzylthiazole-4-carboxamido)-2-(thiophen-2-yl)acetate – '61815, Z997021386 (Method 3).**

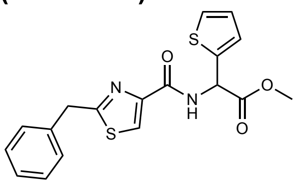

The compound was synthesized from methyl 2-amino-2-(thiophen-2-yl)acetate hydrochloride (Catalog # EN300-60031, 100 mg, 0.48 mmol) and 2-benzyl-1,3-thiazole-4-carboxylic acid (Catalog # EN300-38897, 116 mg, 0.53 mmol).
Yield: 56%; purity, >95% (assessed by LC/MS).
$^1$H NMR (500 MHz, DMSO-$d_6$) δ 8.81 (d, *J* = 7.4 Hz, 1H), 8.21 (d, *J* = 1.4 Hz, 1H), 7.50 (dd, *J* = 5.1, 1.3 Hz, 1H), 7.38 – 7.31 (m, 4H), 7.31 – 7.23 (m, 1H), 7.18 – 7.13 (m, 1H), 7.00 (dd, *J* = 5.1, 3.5 Hz, 1H), 5.87 (d, *J* = 7.4 Hz, 1H), 4.38 (s, 2H), 3.69 (s, 3H).
$^{13}$C NMR (151 MHz, DMSO-$d_6$) δ 171.6, 170.3, 160.3, 148.8, 139.0, 138.1, 129.5, 129.2, 127.6, 127.6, 127.3, 126.9, 125.7, 53.2, 52.1, 38.9, 7.4.
LC/MS (APSI) m/z [M+H] calculated for $C_{18}H_{17}N_2O_3S_2$: 373.1; found: 373.0.

**N-(1-(3-chlorophenyl)-1H-pyrazol-5-yl)-1-(1-cyclopropylethyl)-5-methyl-1H-pyrazole-3-carboxamide – 7902, Z1607601216 (Method 3).**

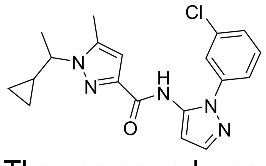

The compound was synthesized from 1-(3-chlorophenyl)-1H-pyrazol-5-amine (Catalog # EN300-68701, 100 mg, 0.52 mmol) and 1-(1-cyclopropylethyl)-5-methyl-1H-pyrazole-3-carboxylic acid (Catalog # EN300-124358 111 mg,0.57 mmol).
Yield: 15%; purity, 92% (assessed by LC/MS).
$^1$H NMR (500 MHz, DMSO-$d_6$) δ 9.88 (s, 1H), 7.74 – 7.66 (m, 2H), 7.58 – 7.53 (m, 1H), 7.49 (t, *J* = 8.0 Hz, 1H), 7.43 (dt, *J* = 7.9, 1.3 Hz, 1H), 6.50 – 6.43 (m, 2H), 3.74 (q, *J* = 7.1, 6.7 Hz, 1H),

2.24 (s, 3H), 1.47 (d, $J$ = 6.7 Hz, 3H), 1.35 (d, $J$ = 9.2 Hz, 1H), 0.56 (dp, $J$ = 8.3, 4.1 Hz, 1H), 0.32 (dddt, $J$ = 35.6, 13.6, 9.2, 4.2 Hz, 3H).
$^{13}$C NMR (126 MHz, DMSO-$d_6$) δ 140.3, 139.7, 136.1, 130.8, 127.1, 122.9, 121.8, 105.6, 103.3, 58.5, 20.5, 17.2, 10.6, 3.7, 3.4.
LC/MS (APSI) m/z [M+H] calculated for $C_{19}H_{21}ClN_5O$: 370.1; found: 370.2.

**(S)-methyl 2-(1-(3-chlorophenyl)-5-methyl-1H-pyrazole-3-carboxamido)-2-(4-methoxyphenyl)acetate – 69543, Z1607615042 (Method 3).**

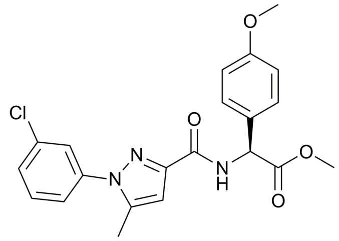


The compound was synthesized from methyl (2S)-2-amino-2-(4-methoxyphenyl)acetate hydrochloride (Catalog # EN300-7466819, 100 mg, 0.43 mmol) and 1-(3-chlorophenyl)-5-methyl-1H-pyrazole-3-carboxylic acid (Catalog # EN300-127105, 112 mg,0.48 mmol).
Yield: 19%; purity, >95% (assessed by LC/MS).
$^{1}$H NMR (500 MHz, DMSO-$d_6$) δ 8.52 (d, $J$ = 7.4 Hz, 1H), 7.75 (t, $J$ = 1.9 Hz, 1H), 7.62 – 7.51 (m, 3H), 7.39 – 7.33 (m, 2H), 6.94 – 6.88 (m, 2H), 6.69 (s, 1H), 5.57 (d, $J$ = 7.4 Hz, 1H), 3.73 (s, 3H), 3.63 (s, 3H), 2.35 (s, 3H).
$^{13}$C NMR (151 MHz, DMSO-$d_6$) δ 171.5, 161.3, 159.6, 146.5, 141.7, 140.6, 134.0, 131.4, 129.7, 129.0, 128.7, 125.2, 123.8, 114.4, 108.1, 55.9, 55.6, 52.8, 12.5.
LC/MS (APSI) m/z [M+H] calculated for $C_{21}H_{21}ClN_3O_4$: 414.1; found: 414.0.

**1-(3-fluorophenyl)-N-(furan-2-yl(phenyl)methyl)-5-methyl-1H-pyrazole-3-carboxamide – 88684, Z1614809888 (Method 3).**

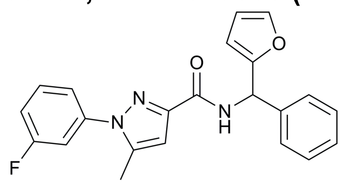


The compound was synthesized from (furan-2-yl)(phenyl)methanamine (Catalog # EN300-14256, 100 mg, 0.58 mmol) and 1-(3-fluorophenyl)-5-methyl-1H-pyrazole-3-carboxylic acid (Catalog # EN300-127391, 140 mg, 0.64 mmol).
Yield: 58%; purity, >95% (assessed by LC/MS).
$^{1}$H NMR (500 MHz, DMSO-$d_6$) δ 8.94 (d, $J$ = 8.9 Hz, 1H), 7.63 – 7.53 (m, 3H), 7.51 – 7.45 (m, 1H), 7.45 – 7.38 (m, 2H), 7.38 – 7.24 (m, 4H), 6.71 (d, $J$ = 1.0 Hz, 1H), 6.41 – 6.34 (m, 2H), 6.17 – 6.13 (m, 1H), 2.36 (s, 3H).
$^{13}$C NMR (151 MHz, DMSO-$d_6$) δ 163.3, 161.1, 154.6, 146.9, 143.0, 141.5, 140.9, 131.5, 128.8, 127.9, 127.9, 121.2, 121.2, 115.6, 112.8, 110.9, 108.2, 50.6, 12.6.
LC/MS (APSI) m/z [M+H] calculated for $C_{22}H_{19}FN_3O_2$: 376.1; found: 376.2.

**methyl 2-(1-(3-fluorophenyl)-5-methyl-1H-pyrazole-3-carboxamido)-2-(thiophen-2-yl)acetate – 51486, Z1615048293 (Method 3).**

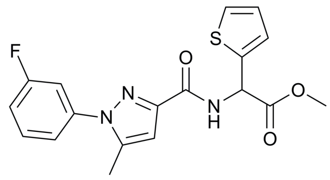

The compound was synthesized from methyl 2-amino-2-(thiophen-2-yl)acetate hydrochloride (Catalog # EN300-60031, 100 mg, 0.48 mmol) and 1-(3-fluorophenyl)-5-methyl-1H-pyrazole-3-carboxylic acid (Catalog # EN300-127391, 117 mg, 0.53 mmol).
Yield: 55%; purity, >95% (assessed by LC/MS).
$^{1}$H NMR (500 MHz, DMSO-*d*$_6$) δ 8.83 (d, *J* = 7.5 Hz, 1H), 7.63 – 7.54 (m, 2H), 7.51 – 7.45 (m, 2H), 7.33 (td, *J* = 8.6, 2.5 Hz, 1H), 7.15 (d, *J* = 3.5 Hz, 1H), 6.99 (dd, *J* = 5.1, 3.5 Hz, 1H), 6.72 (s, 1H), 5.87 (d, *J* = 7.5 Hz, 1H), 3.68 (s, 3H), 2.36 (s, 3H).
$^{13}$C NMR (151 MHz, DMSO-*d*$_6$) δ 170.4, 161.3, 146.3, 141.8, 139.0, 131.4, 127.6, 126.9, 121.3, 115.7, 112.6, 108.1, 53.1, 51.9, 12.5.
LC/MS (APSI) m/z [M+H] calculated for $C_{18}H_{17}FN_3O_3S$: 374.1; found: 374.0.

**methyl (S)-2-(1-(3-fluorophenyl)-5-methyl-1H-pyrazole-3-carboxamido)-2-(4-methoxyphenyl)acetate – 59513, Z1615091760 (Method 3).**

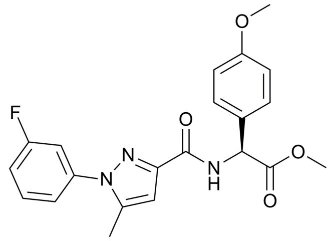

The compound was synthesized from methyl (2S)-2-amino-2-(4-methoxyphenyl)acetate hydrochloride (Catalog # EN300-7466819, 100 mg, 0.48 mmol) and 1-(3-fluorophenyl)-5-methyl-1H-pyrazole-3-carboxylic acid (Catalog # EN300-127391, 116 mg, 0.53 mmol).
Yield: 21%; purity, >95% (assessed by LC/MS).
$^{1}$H NMR (500 MHz, DMSO-*d*$_6$) δ 8.50 (dd, *J* = 7.2, 1.5 Hz, 1H), 7.62 – 7.53 (m, 2H), 7.47 (ddd, *J* = 8.1, 2.5, 1.2 Hz, 1H), 7.41 – 7.29 (m, 3H), 6.95 – 6.88 (m, 2H), 6.69 (d, *J* = 1.1 Hz, 1H), 5.57 (dd, *J* = 7.4, 1.3 Hz, 1H), 3.73 (d, *J* = 1.3 Hz, 3H), 3.63 (d, *J* = 1.3 Hz, 3H), 2.36 (d, *J* = 1.3 Hz, 3H).
$^{13}$C NMR (151 MHz, DMSO-*d*$_6$) δ 171.5, 161.3, 159.6, 146.5, 141.7, 131.5, 129.7, 129.0, 121.3, 115.7, 114.4, 112.8, 108.0, 55.8, 55.6, 52.8, 12.5.
LC/MS (APSI) m/z [M+H] calculated for $C_{21}H_{21}FN_3O_4$: 398.2; found: 398.0.

**1-(3-cyanophenyl)-N-(furan-2-yl(phenyl)methyl)-5-methyl-1H-pyrazole-3-carboxamide – 12565, Z1627119982 (Method 3).**

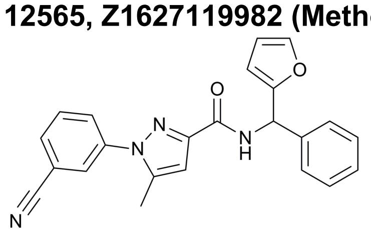

The compound was synthesized from (furan-2-yl)(phenyl)methanamine hydrochloride (Catalog # EN300-6734468, 100 mg, 0.48 mmol) and 1-(3-cyanophenyl)-5-methyl-1H-pyrazole-3-carboxylic acid (Catalog # EN300-128852, 119 mg, 0.53 mmol).

Yield: 51%; purity, >95% (assessed by LC/MS).
$^{1}$H NMR (500 MHz, DMSO-$d_6$) δ 8.98 (d, $J$ = 8.9 Hz, 1H), 8.20 (t, $J$ = 1.9 Hz, 1H), 8.03 – 7.97 (m, 1H), 7.93 (dt, $J$ = 7.8, 1.3 Hz, 1H), 7.74 (t, $J$ = 8.0 Hz, 1H), 7.61 (d, $J$ = 1.8 Hz, 1H), 7.44 – 7.39 (m, 2H), 7.35 (t, $J$ = 7.6 Hz, 2H), 7.31 – 7.25 (m, 1H), 6.73 (s, 1H), 6.41 – 6.34 (m, 2H), 6.14 (d, $J$ = 3.3 Hz, 1H), 2.38 (s, 3H).
$^{13}$C NMR (151 MHz, DMSO-$d_6$) δ 161.0, 154.6, 147.2, 143.0, 141.7, 140.0, 132.3, 131.1, 129.8, 128.8, 128.5, 128.0, 127.8, 118.4, 112.8, 110.9, 108.1, 50.6, 12.5.
LC/MS (APSI) m/z [M+H] calculated for $C_{23}H_{19}N_4O_2$: 383.2; found: 383.1.

**4-(5-(2-(3-chlorophenoxy)propan-2-yl)-1,3,4-oxadiazol-2-yl)-2-methylphthalazin-1(2H)-one – 0450_22, Z1664062039 (Method 1).**

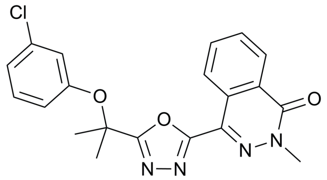


The compound was synthesized from 3-methyl-4-oxo-3,4-dihydrophthalazine-1-carbohydrazide (Catalog # EN300-04897, 112 mg, 0.51 mmol) and 2-(4-chlorophenoxy)-2-methylpropanoic acid (Catalog # EN300-18267, 100 mg, 0.47 mmol).
Yield: 48%; purity, >95% (assessed by LC/MS).
$^{1}$H NMR (500 MHz, DMSO-$d_6$) δ 8.88 (d, $J$ = 8.2 Hz, 1H), 8.35 (dd, $J$ = 8.0, 1.5 Hz, 1H), 8.05 (ddd, $J$ = 8.4, 7.2, 1.4 Hz, 1H), 7.99 – 7.92 (m, 1H), 7.27 (t, $J$ = 8.1 Hz, 1H), 7.15 (ddd, $J$ = 8.1, 2.1, 0.9 Hz, 1H), 6.95 (t, $J$ = 2.2 Hz, 1H), 6.78 – 6.72 (m, 1H), 3.84 (s, 3H), 1.84 (s, 6H).
$^{13}$C NMR (151 MHz, DMSO-$d_6$) δ 168.1, 161.7, 158.8, 155.5, 134.6, 133.8, 133.0, 131.4, 130.2, 127.4, 126.9, 126.6, 124.6, 122.5, 120.8, 76.2, 26.0.
LC/MS (APSI) m/z [M+H] calculated for $C_{20}H_{18}ClN_4O_3$: 397.1; found: 397.0.

**N-((4-chlorophenyl)(1-methyl-1H-pyrazol-3-yl)methyl)-1-(3-fluorophenyl)-5-methyl-1H-pyrazole-3-carboxamide – 17429, Z1686630553 (Method 3).**

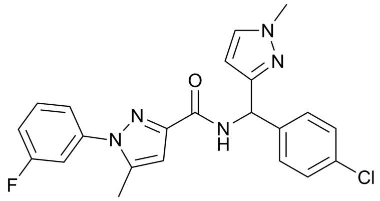


The compound was synthesized from (4-chlorophenyl)(1-methyl-1H-pyrazol-3-yl)methanamine (Catalog # EN300-126692, 100 mg, 0.45 mmol) and 1-(3-fluorophenyl)-5-methyl-1H-pyrazole-3-carboxylic acid (Catalog # EN300-127391, 110 mg, 0.5 mmol).
Yield: 42%; purity, >95% (assessed by LC/MS).
$^{1}$H NMR (500 MHz, DMSO-$d_6$) δ 8.66 (d, $J$ = 8.4 Hz, 1H), 7.63 – 7.53 (m, 3H), 7.50 – 7.45 (m, 1H), 7.41 – 7.30 (m, 5H), 6.68 (s, 1H), 6.25 (d, $J$ = 8.3 Hz, 1H), 6.18 (d, $J$ = 2.2 Hz, 1H), 3.78 (s, 3H), 2.35 (s, 3H).
$^{13}$C NMR (151 MHz, DMSO-$d_6$) δ 160.9, 151.7, 146.9, 141.7, 132.2, 131.5, 129.4, 128.6, 121.4, 115.7, 112.9, 108.0, 104.3, 50.8, 38.9, 12.5.
LC/MS (APSI) m/z [M+H] calculated for $C_{22}H_{20}ClFN_5O$: 424.1; found: 424.2.

**methyl 2-(1-(3-cyanophenyl)-5-methyl-1H-pyrazole-3-carboxamido)-2-(thiophen-3-yl)acetate – 31604, Z1711777464 (Method 3).**

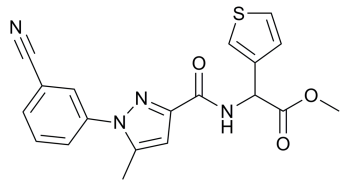

The compound was synthesized from methyl 2-amino-2-(thiophen-3-yl)acetate hydrochloride (Catalog # EN300-140330, 100 mg, 0.48 mmol) and 1-(3-cyanophenyl)-5-methyl-1H-pyrazole-3-carboxylic acid (Catalog # EN300-128852, 120 mg, 0.53 mmol).
Yield: 64%; purity, >95% (assessed by LC/MS).
$^{1}$H NMR (500 MHz, DMSO-$d_6$) δ 8.70 (d, $J$ = 7.7 Hz, 1H), 8.20 (t, $J$ = 2.0 Hz, 1H), 7.99 (ddd, $J$ = 8.2, 2.3, 1.1 Hz, 1H), 7.94 (dt, $J$ = 7.8, 1.4 Hz, 1H), 7.75 (t, $J$ = 8.0 Hz, 1H), 7.60 – 7.54 (m, 1H), 7.52 (dd, $J$ = 5.0, 2.9 Hz, 1H), 7.19 (dd, $J$ = 5.0, 1.4 Hz, 1H), 6.73 (s, 1H), 5.76 (d, $J$ = 7.7 Hz, 1H), 3.66 (s, 3H), 2.38 (s, 3H).
$^{13}$C NMR (151 MHz, DMSO-$d_6$) δ 171.0, 161.3, 146.8, 141.9, 140.0, 137.0, 132.4, 131.2, 129.9, 128.5, 127.9, 127.1, 124.7, 118.4, 112.8, 108.3, 52.9, 52.1, 12.5.
LC/MS (APSI) m/z [M+H] calculated for C19H17N4O3S: 381.1; found: 381.0.

**methyl (1S,3aS,6aR)-2-(1-(3-chlorophenyl)-5-methyl-1H-pyrazole-3-carbonyl)octahydrocyclopenta[c]pyrrole-1-carboxylate – 10010, Z1817976028 (Method 3).**

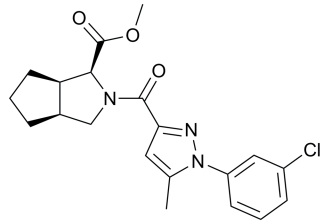

The compound was synthesized from methyl rac-methyl (1R,3aR,6aS)-octahydrocyclopenta[c]pyrrole-1-carboxylate hydrochloride (Catalog # EN300-6491228, 100 mg, 0.49 mmol) and 1-(3-chlorophenyl)-5-methyl-1H-pyrazole-3-carboxylic acid (Catalog # EN300-127105, 127 mg, 0.54 mmol).
Yield: 36%; purity, >95% (assessed by LC/MS).
$^{1}$H NMR (500 MHz, DMSO-$d_6$) δ 7.62 (q, $J$ = 1.7 Hz, 1H), 7.59 – 7.49 (m, 3H), 6.64 (s, 1H), 5.32 (d, $J$ = 9.2 Hz, 1H), 4.72 (d, $J$ = 8.8 Hz, 1H), 3.63 (d, $J$ = 1.1 Hz, 2H), 3.50 (d, $J$ = 1.2 Hz, 2H), 3.05 (ddd, $J$ = 31.7, 14.3, 8.7 Hz, 1H), 2.80 – 2.74 (m, 1H), 2.64 (td, $J$ = 8.6, 3.6 Hz, 1H), 2.35 (d, $J$ = 12.4 Hz, 3H), 1.69 (tdd, $J$ = 13.9, 10.2, 6.1 Hz, 2H), 1.62 – 1.52 (m, 2H), 1.38 – 1.29 (m, 1H).
$^{13}$C NMR (151 MHz, DMSO-$d_6$) δ 172.6, 161.2, 147.9, 134.0, 131.5, 128.7, 124.3, 122.9, 110.3, 64.1, 51.9, 44.3, 41.0, 29.3, 27.8, 26.2, 12.5.
LC/MS (APSI) m/z [M+H] calculated for $C_{20}H_{23}ClN_3O_3$: 388.1; found: 388.0.

**(2,3-dihydrobenzo[b][1,4]oxathiin-2-yl)methyl 1-(4-fluorobenzyl)-5-methyl-1H-1,2,3-triazole-4-carboxylate – 61926, Z1895651057 (Method 2).**

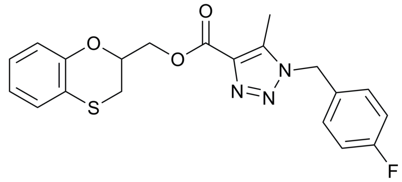

The compound was synthesized from methyl (2,3-dihydro-1,4-benzoxathiin-2-yl)methanol (Catalog # EN300-59707, 100 mg, 0.55 mmol) and 1-[(4-fluorophenyl)methyl]-5-methyl-1H-1,2,3-triazole-4-carboxylic acid (Catalog # EN300-180745, 142 mg, 0.6 mmol).

Yield: 28%; purity, >95% (assessed by LC/MS).
$^{1}$H NMR (500 MHz, DMSO-$d_6$) δ 7.35 – 7.24 (m, 3H), 7.19 (td, $J$ = 8.8, 2.5 Hz, 3H), 7.00 (t, $J$ = 7.6 Hz, 2H), 5.64 (s, 2H), 5.43 (qd, $J$ = 4.5, 2.2 Hz, 1H), 4.48 (dd, $J$ = 13.2, 3.1 Hz, 1H), 4.29 (dd, $J$ = 13.2, 4.4 Hz, 1H), 3.31 – 3.21 (m, 2H), 2.50 (s, 3H).
$^{13}$C NMR (151 MHz, DMSO-$d_6$) δ 160.5, 159.4, 139.5, 136.1, 131.7, 130.2, 128.8, 126.8, 124.2, 122.3, 116.1, 72.6, 50.5, 33.5, 9.4.
LC/MS (APSI) m/z [M+H] calculated for $C_{20}H_{19}FN_3O_3S$: 400.1; found: 400.2.

**3-(benzo[d][1,3]dioxol-5-ylmethyl)-5-(2-(cyclopropylmethyl)-4-methyloxazol-5-yl)-1,2,4-oxadiazole – 97058, Z2156297058 (Method 5).**

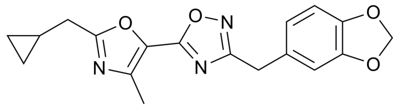

The compound was synthesized from 2-(1,3-dioxaindan-5-yl)acetonitrile (Catalog # EN300-26831, 100 mg, 0.62 mmol) and 2-(cyclopropylmethyl)-4-methyl-1,3-oxazole-5-carboxylic acid (Catalog # EN300-172627, 124 mg, 0.68 mmol).
Yield: 19%; purity, >95% (assessed by LC/MS).
$^{1}$H NMR (500 MHz, DMSO-$d_6$) δ 6.88 (d, $J$ = 1.7 Hz, 1H), 6.85 (d, $J$ = 7.9 Hz, 1H), 6.78 (dd, $J$ = 7.9, 1.8 Hz, 1H), 5.97 (s, 2H), 4.05 (s, 2H), 2.75 (d, $J$ = 7.0 Hz, 2H), 2.43 (s, 3H), 1.09 (tq, $J$ = 10.2, 3.6, 2.6 Hz, 1H), 0.56 – 0.45 (m, 2H), 0.24 (dt, $J$ = 6.1, 4.3 Hz, 2H).
$^{13}$C NMR (126 MHz, DMSO-$d_6$) δ 170.1, 167.2, 166.7, 147.8, 146.6, 144.4, 133.2, 129.5, 122.5, 109.8, 108.7, 101.4, 32.4, 31.2, 13.1, 8.7, 4.9.
LC/MS (APSI) m/z [M+H] calculated for $C_{18}H_{18}N_3O_4$: 340.1; found: 340.0.

**3-((6-bromobenzo[d][1,3]dioxol-5-yl)methyl)-5-(2-(cyclopropylmethyl)-4-methyloxazol-5-yl)-1,2,4-oxadiazole – 7019, Z2157628646 (Method 5).**

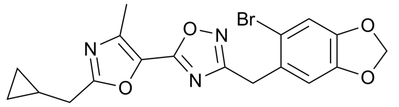

The compound was synthesized from 2-(6-bromo-1,3-dioxaindan-5-yl)acetonitrile (Catalog # EN300-53536, 100 mg, 0.42 mmol) and 2-(cyclopropylmethyl)-4-methyl-1,3-oxazole-5-carboxylic acid (Catalog # EN300-172627, 83 mg, 0.46 mmol).
Yield: 34%; purity, >95% (assessed by LC/MS).
$^{1}$H NMR (500 MHz, DMSO-$d_6$) δ 7.21 (d, $J$ = 1.5 Hz, 1H), 7.05 (s, 1H), 6.06 (s, 2H), 4.16 (s, 2H), 2.75 (d, $J$ = 7.0 Hz, 2H), 2.43 (s, 3H), 1.14 – 1.05 (m, 1H), 0.55 – 0.46 (m, 2H), 0.27 – 0.21 (m, 2H).
$^{13}$C NMR (151 MHz, DMSO-$d_6$) δ 169.2, 167.3, 166.7, 148.0, 147.7, 144.5, 133.1, 128.2, 114.9, 112.8, 111.5, 102.5, 32.4, 32.4, 13.1, 8.7, 4.9.
LC/MS (APSI) m/z [M+H] calculated for $C_{18}H_{17}BrN_3O_4$: 420.0; found: 420.0.

**3-((7-bromobenzo[d][1,3]dioxol-5-yl)methyl)-5-(2-(cyclopropylmethyl)-4-methyloxazol-5-yl)-1,2,4-oxadiazole – 53217, Z2157653217 (Method 5).**

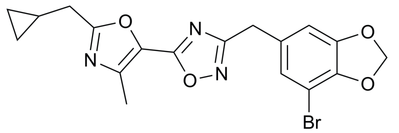

The compound was synthesized from 2-(7-bromo-1,3-dioxaindan-5-yl)acetonitrile (Catalog # EN300-54744, 100 mg, 0.42 mmol) and 2-(cyclopropylmethyl)-4-methyl-1,3-oxazole-5-carboxylic acid (Catalog # EN300-172627, 83 mg, 0.46 mmol).
Yield: 12%; purity, >95% (assessed by LC/MS).

$^1$H NMR (500 MHz, DMSO-$d_6$) δ 7.02 (d, *J* = 1.5 Hz, 1H), 6.90 (d, *J* = 1.5 Hz, 1H), 6.09 (s, 2H), 4.08 (s, 2H), 2.76 (d, *J* = 7.0 Hz, 2H), 2.43 (s, 3H), 1.14 – 1.01 (m, 1H), 0.55 – 0.46 (m, 2H), 0.27 – 0.21 (m, 2H).
$^{13}$C NMR (126 MHz, DMSO-$d_6$) δ 169.3, 166.7, 166.3, 148.0, 144.6, 144.0, 132.6, 130.9, 124.7, 108.8, 101.7, 99.4, 31.9, 30.4, 12.6, 8.2, 4.4.
LC/MS (APSI) m/z [M+H] calculated for $C_{18}H_{17}BrN_3O_4$: 420.0; found: 420.0.

**N-((2,3-dihydrobenzo[b][1,4]oxathiin-2-yl)methyl)-1-(4-fluorobenzyl)-5-methyl-1H-1,2,3-triazole-4-carboxamide – 22139, Z2454164835 (Method 3).**

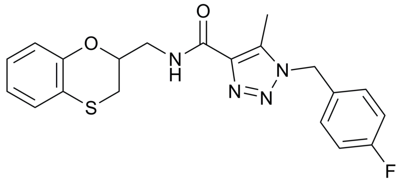

The compound was synthesized from (2,3-dihydro-1,4-benzoxathiin-2-yl)methanamine hydrochloride (Catalog # EN300-258384, 100 mg, 0.46 mmol) and 1-[(4-fluorophenyl)methyl]-5-methyl-1H-1,2,3-triazole-4-carboxylic acid (Catalog # EN300-180745, 120 mg, 0.51 mmol).
Yield: 42%; purity, >95% (assessed by LC/MS).
$^1$H NMR (500 MHz, DMSO-$d_6$) δ 8.68 (t, *J* = 6.1 Hz, 1H), 7.26 (dd, *J* = 8.6, 5.6 Hz, 2H), 7.22 – 7.14 (m, 2H), 7.06 (dd, *J* = 7.7, 1.6 Hz, 1H), 6.97 (tt, *J* = 7.2, 3.6 Hz, 1H), 6.86 – 6.76 (m, 2H), 5.61 (s, 2H), 4.33 (dtd, *J* = 8.3, 6.2, 2.0 Hz, 1H), 3.62 (dt, *J* = 13.7, 6.0 Hz, 1H), 3.53 (dt, *J* = 13.1, 6.3 Hz, 1H), 3.17 (dd, *J* = 13.2, 2.1 Hz, 1H), 2.96 (dd, *J* = 13.3, 8.5 Hz, 1H), 2.46 (s, 3H).
$^{13}$C NMR (151 MHz, DMSO-$d_6$) δ 161.7, 151.5, 138.6, 136.6, 130.2, 127.6, 126.1, 121.9, 118.7, 117.8, 116.2, 73.5, 50.3, 42.4, 27.6, 8.8.
LC/MS (APSI) m/z [M+H] calculated for $C_{20}H_{20}FN_4O_2S$: 399.1; found: 399.0.

**1-(1-(4-(4-fluorophenyl)tetrahydro-2H-pyran-4-yl)-1H-1,2,3-triazol-4-yl)-3-phenylpropan-1-one – 7337, Z2527311170 (Method 6).**

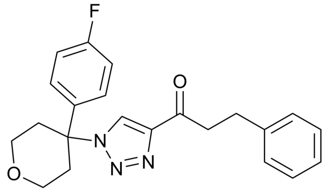

The compound was synthesized from 4-azido-4-(4-fluorophenyl)oxane (Catalog # EN300-71746, 100 mg, 0.45 mmol) and 5-phenylpent-1-yn-3-one (Catalog # EN300-180283, 78 mg, 0.5 mmol).
Yield: 27%; purity, >95% (assessed by LC/MS).
$^1$H NMR (400 MHz, DMSO-$d_6$) δ 8.97 (s, 1H), 7.30 (dd, *J* = 8.7, 5.3 Hz, 2H), 7.23 (d, *J* = 5.2 Hz, 4H), 7.14 (s, 1H), 7.07 (t, *J* = 8.6 Hz, 2H), 3.82 (d, *J* = 12.0 Hz, 2H), 3.43 – 3.31 (m, 4H), 3.03 – 2.95 (m, 6H).
$^{13}$C NMR (151 MHz, DMSO-$d_6$) δ 193.4, 147.6, 141.5, 139.9, 128.8, 128.7, 127.8, 127.1, 126.4, 116.1, 64.6, 63.4, 41.1, 35.6, 29.5.
LC/MS (APSI) m/z [M+Na] calculated for $C_{22}H_{22}FN_3NaO_2$: 402.2; found: 402.0.

**2-(7,7-difluorobicyclo[4.1.0]heptan-1-yl)-1-(3-(3-methoxyphenyl)-3-methylpyrrolidin-1-yl)ethan-1-one – 7218, Z2645906126 (Method 3).**

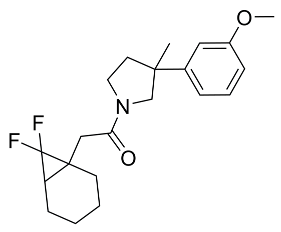

The compound was synthesized from 3-(3-methoxyphenyl)-3-methylpyrrolidine hydrochloride (Catalog # EN300-304264, 100 mg, 0.44 mmol) and 2-{7,7-difluorobicyclo[4.1.0]heptan-1-yl}acetic acid (Catalog # EN300-298624, 92 mg, 0.48 mmol).
Yield: 56%; purity, >95% (assessed by LC/MS).
$^1$H NMR (500 MHz, DMSO-$d_6$) δ 7.23 (tt, $J$ = 7.9, 2.2 Hz, 1H), 6.89 – 6.79 (m, 2H), 6.81 – 6.75 (m, 1H), 3.73 (dd, $J$ = 2.3, 1.5 Hz, 3H), 3.68 – 3.64 (m, 1H), 3.64 – 3.60 (m, 1H), 3.51 (d, $J$ = 9.7 Hz, 1H), 3.42 (q, $J$ = 3.9 Hz, 1H), 3.42 – 3.36 (m, 1H), 2.64 – 2.51 (m, 1H), 2.51 – 2.30 (m, 1H), 2.19 – 2.08 (m, 1H), 2.10 – 1.99 (m, 1H), 1.73 (td, $J$ = 12.8, 5.9 Hz, 1H), 1.60 – 1.52 (m, 1H), 1.38 (dtd, $J$ = 12.6, 8.9, 7.7, 4.7 Hz, 1H), 1.28 (s, 4H), 1.24 (dd, $J$ = 4.5, 2.4 Hz, 3H), 1.18 (tt, $J$ = 14.0, 7.8 Hz, 1H).
$^{13}$C NMR (151 MHz, DMSO-$d_6$) δ 159.7, 149.0, 129.8, 118.3, 112.4, 111.8, 55.4, 46.4, 44.4, 38.7, 37.8, 36.0, 27.7, 23.5, 23.0, 21.0.
LC/MS (APSI) m/z [M+H] calculated for $C_{21}H_{28}F_2NO_2$: 364.2; found: 364.2.

**5-(2-(cyclopropylmethyl)-4-methyloxazol-5-yl)-3-((3,4-dihydro-2H-benzo[b][1,4]dioxepin-7-yl)methyl)-1,2,4-oxadiazole – 7019_1, Z3555992696 (Method 5).**

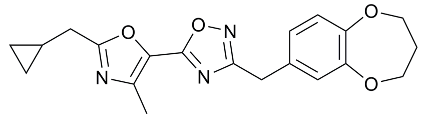

The compound was synthesized from 2-(3,4-dihydro-2H-1,5-benzodioxepin-7-yl)acetonitrile (Catalog # EN300-1602849, 100 mg, 0.53 mmol) and 2-(cyclopropylmethyl)-4-methyl-1,3-oxazole-5-carboxylic acid (Catalog # EN300-172627, 105 mg, 0.58 mmol).
Yield: 19%; purity, >95% (assessed by LC/MS).
$^1$H NMR (500 MHz, DMSO-$d_6$) δ 6.93 – 6.86 (m, 2H), 6.86 (dd, $J$ = 8.2, 2.1 Hz, 1H), 4.11 – 4.01 (m, 6H), 2.75 (d, $J$ = 7.1 Hz, 2H), 2.43 (s, 2H), 2.42 (s, 1H), 2.06 (p, $J$ = 5.5 Hz, 2H), 1.14 – 1.05 (m, 1H), 0.55 – 0.46 (m, 2H), 0.27 – 0.21 (m, 2H).
$^{13}$C NMR (151 MHz, DMSO-$d_6$) δ 169.9, 167.2, 166.7, 151.3, 150.4, 144.4, 133.2, 131.0, 124.2, 122.4, 122.1, 70.9, 70.8, 32.4, 32.0, 30.7, 13.1, 8.7, 4.9.
LC/MS (APSI) m/z [M+H] calculated for $C_{20}H_{22}N_3O_4$: 368.2; found: 368.2.

**3-((6-bromobenzo[d][1,3]dioxol-5-yl)methyl)-5-(2-(cyclopropylmethyl)thiazol-5-yl)-1,2,4-oxadiazole – 7019_analog_11, Z4766160626 (Method 5).**

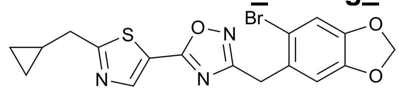

The compound was synthesized from 2-(6-bromo-1,3-dioxaindan-5-yl)acetonitrile (Catalog # EN300-53536, 100 mg, 0.42 mmol) and 2-(cyclopropylmethyl)-1,3-thiazole-5-carboxylic acid (Catalog # EN300-379019, 84 mg, 0.46 mmol).
Yield: 17%; purity, >95% (assessed by LC/MS).
$^1$H NMR (500 MHz, DMSO-$d_6$) δ 8.53 (s, 1H), 7.22 (s, 1H), 7.06 (s, 1H), 6.06 (s, 2H), 4.15 (s, 2H), 2.97 (d, $J$ = 7.1 Hz, 2H), 1.19 – 1.08 (m, 1H), 0.63 – 0.54 (m, 2H), 0.33 (dt, $J$ = 5.9, 2.9 Hz, 2H).
$^{13}$C NMR (151 MHz, DMSO-$d_6$) δ 178.0, 169.8, 169.5, 148.0, 147.7, 147.1, 128.1, 120.4, 114.9, 112.8, 111.5, 102.5, 37.7, 32.4, 11.2, 5.3.

LC/MS (APSI) m/z [M+H] calculated for $C_{17}H_{15}BrN_3O_3S$: 420.0; found: 420.0.

**4-(5-(2-(4-methoxyphenoxy)propan-2-yl)-1,3,4-oxadiazol-2-yl)-2-methylphthalazin-1(2H)-one – 9753, Z4877479753 (Method 1).**

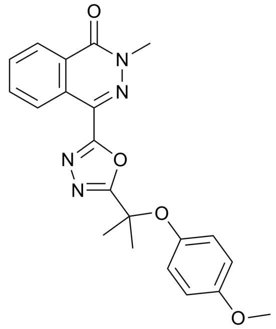

The compound was synthesized from 3-methyl-4-oxo-3,4-dihydrophthalazine-1-carbohydrazide (Catalog # EN300-04897, 114 mg, 0.52 mmol) and 2-(4-methoxyphenoxy)-2-methylpropanoic acid (Catalog # EN300-298355, 100 mg, 0.48 mmol).
Yield: 11%; purity, >95% (assessed by LC/MS).
$^1$H NMR (500 MHz, DMSO-$d_6$) δ 8.87 (d, $J$ = 7.8 Hz, 1H), 8.35 (s, 1H), 8.03 (s, 1H), 7.95 (s, 1H), 6.79 (d, $J$ = 8.3 Hz, 2H), 6.71 (d, $J$ = 9.3 Hz, 2H), 3.85 (s, 3H), 3.66 (s, 3H), 1.79 (s, 6H).
$^{13}$C NMR (101 MHz, DMSO-$d_6$) δ 158.8, 156.4, 147.7, 134.6, 133.0, 127.4, 126.9, 126.6, 124.1, 114.8, 75.8, 55.7, 25.9.
LC/MS (APSI) m/z [M+H] calculated for $C_{21}H_{21}N_4O_4$: 393.2; found: 393.0.

**1-benzyl-N-((2,3-dihydrobenzo[b][1,4]oxathiin-2-yl)methyl)-1H-1,2,3-triazole-4-carboxamide – 78184, Z4877482092 (Method 3).**

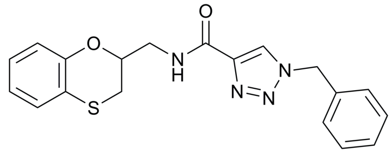

The compound was synthesized from (2,3-dihydro-1,4-benzoxathiin-2-yl)methanamine hydrochloride (Catalog # EN300-258384, 100 mg, 0.46 mmol) and 1-benzyl-1H-1,2,3-triazole-4-carboxylic acid (Catalog # EN300-26964, 103 mg, 0.51 mmol).
Yield: 72%; purity, >95% (assessed by LC/MS).
$^1$H NMR (500 MHz, DMSO-$d_6$) δ 8.75 (t, $J$ = 6.0 Hz, 1H), 8.67 (d, $J$ = 2.7 Hz, 1H), 7.40 – 7.29 (m, 5H), 7.05 (dd, $J$ = 7.8, 1.8 Hz, 1H), 6.97 (td, $J$ = 7.8, 1.8 Hz, 1H), 6.86 – 6.77 (m, 2H), 5.64 (s, 2H), 4.37 – 4.29 (m, 1H), 3.63 (dt, $J$ = 12.6, 6.0 Hz, 1H), 3.52 (dt, $J$ = 13.2, 6.1 Hz, 1H), 3.17 (dd, $J$ = 13.4, 2.3 Hz, 1H), 2.97 (dd, $J$ = 13.2, 8.4 Hz, 1H).
$^{13}$C NMR (151 MHz, DMSO-$d_6$) δ 160.5, 151.4, 143.2, 136.1, 129.3, 128.7, 128.5, 127.6, 127.2, 126.1, 121.9, 118.7, 117.7, 73.4, 53.6, 42.6, 27.5.
LC/MS (APSI) m/z [M+H] calculated for $C_{19}H_{19}N_4O_2S$+: 367.1; found: 367.4.

**N-(furan-2-yl(phenyl)methyl)-5-methyl-1-(3-(trifluoromethyl)phenyl)-1H-pyrazole-3-carboxamide – 1486_32, Z4967431081 (Method 3).**

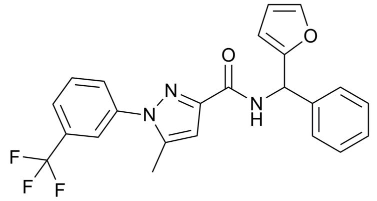

The compound was synthesized from (furan-2-yl)(phenyl)methanamine hydrochloride (Catalog # EN300-6734468, 100 mg, 0.48 mmol) and 5-methyl-1-[3-(trifluoromethyl)phenyl]-1H-pyrazole-3-carboxylic acid (Catalog # EN300-260239, 142 mg, 0.53 mmol).
Yield: 54%; purity, >95% (assessed by LC/MS).
$^{1}$H NMR (500 MHz, DMSO-$d_6$) δ 9.01 (d, *J* = 8.8 Hz, 1H), 8.00 (d, *J* = 2.0 Hz, 1H), 7.98 – 7.93 (m, 1H), 7.87 – 7.82 (m, 1H), 7.78 (t, *J* = 7.9 Hz, 1H), 7.61 (dd, *J* = 1.9, 0.9 Hz, 1H), 7.44 – 7.39 (m, 2H), 7.34 (t, *J* = 7.5 Hz, 2H), 7.31 – 7.24 (m, 1H), 6.74 (d, *J* = 1.0 Hz, 1H), 6.41 – 6.35 (m, 2H), 6.16 – 6.11 (m, 1H), 2.36 (s, 3H).
$^{13}$C NMR (151 MHz, DMSO-$d_6$) δ 161.1, 154.6, 147.2, 143.0, 141.7, 140.4, 140.0, 131.1, 129.2, 128.8, 127.9, 127.9, 125.3, 110.9, 108.3, 108.2, 50.6, 12.5.
LC/MS (APSI) m/z [M+H] calculated for $C_{23}H_{19}F_3N_3O_2$: 426.1; found: 426.2.

**N-(furan-2-yl(phenyl)methyl)-N,5-dimethyl-1-(3-(trifluoromethyl)phenyl)-1H-pyrazole-3-carboxamide – 1486_33, Z4967431082 (Method 3).**

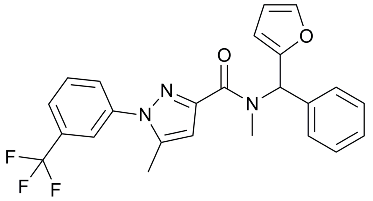


The compound was synthesized from [(furan-2-yl)(phenyl)methyl](methyl)amine (Catalog # EN300-8510002, 100 mg, 0.53 mmol) and 5-methyl-1-[3-(trifluoromethyl)phenyl]-1H-pyrazole-3-carboxylic acid (Catalog # EN300-260239, 159 mg, 0.59 mmol).
Yield: 67%; purity, >95% (assessed by LC/MS).
$^{1}$H NMR (500 MHz, DMSO-$d_6$) δ 7.95 – 7.89 (m, 1H), 7.89 – 7.79 (m, 1H), 7.79 (s, 1H), 7.79 – 7.66 (m, 2H), 7.38 (dq, *J* = 13.0, 7.4, 5.5 Hz, 3H), 7.31 (q, *J* = 7.7 Hz, 1H), 7.23 – 7.17 (m, 2H), 7.04 (s, 1H), 6.69 (d, *J* = 15.3 Hz, 1H), 6.48 (dt, *J* = 10.4, 2.6 Hz, 1H), 3.10 (s, 2H), 2.81 (s, 1H), 2.38 (d, *J* = 8.2 Hz, 3H).
$^{13}$C NMR (151 MHz, DMSO-$d_6$) δ 163.7, 152.8, 147.7, 143.8, 140.8, 140.0, 137.7, 131.2, 129.2, 128.4, 128.2, 127.9, 125.0, 121.8, 111.0, 110.2, 110.0, 59.1, 34.0, 12.5.
LC/MS (APSI) m/z [M+H] calculated for $C_{24}H_{21}F_3N_3O_2$: 440.2; found: 440.0.

**N-(di(furan-2-yl)methyl)-5-methyl-1-(3-(trifluoromethyl)phenyl)-1H-pyrazole-3-carboxamide – 1486_41, Z4967431090 (Method 3).**

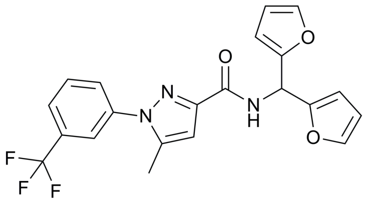


The compound was synthesized from bis(furan-2-yl)methanamine (Catalog # EN300-1258794, 100 mg, 0.61 mmol) and 5-methyl-1-[3-(trifluoromethyl)phenyl]-1H-pyrazole-3-carboxylic acid (Catalog # EN300-260239, 182 mg, 0.67 mmol).
Yield: 54%; purity, >95% (assessed by LC/MS).
$^{1}$H NMR (500 MHz, DMSO-$d_6$) δ 8.95 (d, *J* = 8.9 Hz, 1H), 8.00 (s, 1H), 7.95 (d, *J* = 8.0 Hz, 1H), 7.84 (d, *J* = 7.8 Hz, 1H), 7.78 (t, *J* = 7.9 Hz, 1H), 7.62 (d, *J* = 1.8 Hz, 2H), 6.76 (s, 1H), 6.46 (d, *J* = 8.8 Hz, 1H), 6.42 (t, *J* = 2.6 Hz, 2H), 6.32 (d, *J* = 3.2 Hz, 2H), 2.37 (s, 3H).
$^{13}$C NMR (151 MHz, DMSO-$d_6$) δ 161.0, 152.3, 146.9, 143.1, 141.7, 140.0, 131.0, 129.1, 125.3, 123.2, 122.0, 111.0, 108.4, 108.1, 44.8, 12.5.
LC/MS (APSI) m/z [M+H] calculated for $C_{21}H_{17}F_3N_3O_3$: 416.4; found: 416.1.

**(5-methyl-1-(3-(trifluoromethyl)phenyl)-1H-pyrazol-3-yl)(1-(tetrahydrofuran-2-yl)-2-azaspiro[3.3]heptan-2-yl)methanone – 10010_03, Z5030903737 (Method 3).**

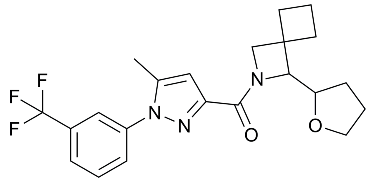

The compound was synthesized from 1-(oxolan-2-yl)-2-azaspiro[3.3]heptane (Catalog # EN300-216770, 100 mg, 0.6 mmol) and 5-methyl-1-[3-(trifluoromethyl)phenyl]-1H-pyrazole-3-carboxylic acid (Catalog # EN300-260239, 178 mg, 0.66 mmol).
Yield: 66%; purity, >95% (assessed by LC/MS).
$^{1}$H NMR (500 MHz, DMSO-$d_6$) δ 7.92 (d, $J$ = 9.4 Hz, 2H), 7.84 (d, $J$ = 7.9 Hz, 1H), 7.79 (t, $J$ = 7.8 Hz, 1H), 6.63 (s, 1H), 4.41 – 4.28 (m, 1H), 4.06 (d, $J$ = 3.4 Hz, 2H), 3.80 (q, $J$ = 7.0 Hz, 1H), 3.67 (td, $J$ = 16.1, 14.2, 7.2 Hz, 1H), 2.47 – 2.40 (m, 1H), 2.36 (s, 4H), 2.08 (s, 3H), 1.88 (d, $J$ = 8.1 Hz, 3H), 1.74 (dq, $J$ = 13.2, 7.2, 6.2 Hz, 3H).
$^{13}$C NMR (151 MHz, DMSO-$d_6$) δ 163.1 – 163.0 (m), 141.0, 140.1, 131.2, 129.2, 125.2, 121.6, 109.3, 79.0, 71.7, 68.0, 65.6, 42.2, 34.2, 29.5, 28.7, 25.6, 16.5, 12.4.
LC/MS (APSI) m/z [M+H] calculated for $C_{22}H_{25}F_3N_3O_2$: 420.2; found: 420.2.

**methyl (1S,3aS,6aR)-2-(5-methyl-1-(3-(trifluoromethyl)phenyl)-1H-pyrazole-3-carbonyl)octahydrocyclopenta[c]pyrrole-1-carboxylate – 10010_01, Z5030906439 (Method 3).**

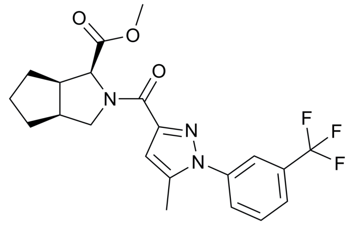

The compound was synthesized from rac-methyl (1R,3aR,6aS)-octahydrocyclopenta[c]pyrrole-1-carboxylate hydrochloride (Catalog # EN300-6491228, 100 mg, 0.49 mmol) and 5-methyl-1-[3-(trifluoromethyl)phenyl]-1H-pyrazole-3-carboxylic acid (Catalog # EN300-260239, 145 mg, 0.54 mmol).
Yield: 64%; purity, >95% (assessed by LC/MS).
$^{1}$H NMR (500 MHz, DMSO-$d_6$) δ 7.97 – 7.91 (m, 1H), 7.90 – 7.75 (m, 3H), 6.68 – 6.64 (m, 1H), 5.33 (d, $J$ = 9.3 Hz, 1H), 4.44 (dd, $J$ = 11.6, 8.7 Hz, 1H), 4.16 (dd, $J$ = 12.1, 8.8 Hz, 1H), 3.63 (s, 1H), 3.45 (s, 1H), 3.39 (dd, $J$ = 11.7, 8.9 Hz, 1H), 3.12 – 2.98 (m, 1H), 2.89 (p, $J$ = 8.3 Hz, 1H), 2.37 (d, $J$ = 11.5 Hz, 3H), 1.69 (tdd, $J$ = 19.5, 9.5, 5.5 Hz, 2H), 1.64 – 1.52 (m, 2H), 1.49 (dddd, $J$ = 15.6, 13.0, 6.8, 3.7 Hz, 1H), 1.38 – 1.29 (m, 1H).
$^{13}$C NMR (151 MHz, DMSO-$d_6$) δ 172.6, 161.2, 148.2, 140.9, 140.1, 131.2, 129.3, 125.3, 121.2, 110.4, 110.1, 64.1, 54.3, 51.9, 44.3, 41.0, 29.4, 28.3, 26.2, 12.5.
LC/MS (APSI) m/z [M+H] calculated for $C_{21}H_{23}F_3N_3O_3$: 422.2; found: 422.1.

**methyl (1R,2S,5S)-3-(5-methyl-1-(3-(trifluoromethyl)phenyl)-1H-pyrazole-3-carbonyl)-3-azabicyclo[3.2.0]heptane-2-carboxylate – 10010_02, Z5030906448 (Method 3).**

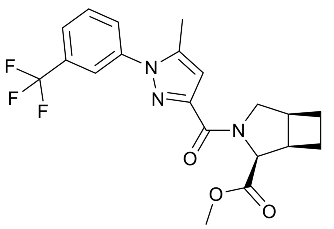

The compound was synthesized from rac-methyl (1R,2S,5S)-3-azabicyclo[3.2.0]heptane-2-carboxylate hydrochloride (Catalog # EN300-1717544, 100 mg, 0.52 mmol) and 5-methyl-1-[3-(trifluoromethyl)phenyl]-1H-pyrazole-3-carboxylic acid (Catalog # EN300-260239, 155 mg, 0.58 mmol).
Yield: 70%; purity, >95% (assessed by LC/MS).
$^1$H NMR (500 MHz, DMSO-$d_6$) δ 7.98 – 7.91 (m, 1H), 7.90 – 7.75 (m, 3H), 6.69 (s, 1H), 4.20 (dd, *J* = 12.6, 8.9 Hz, 1H), 3.64 (s, 1H), 3.52 (dd, *J* = 12.7, 6.0 Hz, 1H), 3.45 (s, 2H), 3.20 (qd, *J* = 8.6, 5.6 Hz, 1H), 3.03 (d, *J* = 8.1 Hz, 1H), 2.84 (s, 1H), 2.38 (d, *J* = 7.9 Hz, 3H), 2.17 – 2.06 (m, 1H), 2.06 – 1.94 (m, 1H), 1.81 – 1.59 (m, 2H).
$^{13}$C NMR (151 MHz, DMSO-$d_6$) δ 172.1, 161.7, 148.3, 140.9, 140.1, 131.2, 129.3, 128.2, 125.3, 121.1, 110.5, 110.2, 65.1, 55.7, 52.0, 42.4, 35.7, 23.9, 21.3, 12.5.
LC/MS (APSI) m/z [M+H] calculated for $C_{20}H_{21}F_3N_3O_3$: 408.2; found: 408.1.

**N-(di(furan-2-yl)methyl)-N,5-dimethyl-1-(3-(trifluoromethyl)phenyl)-1H-pyrazole-3-carboxamide – 1486_43, Z5199554388 (Method 3).**

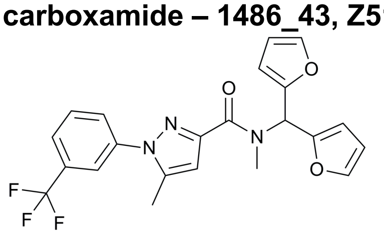

The compound was synthesized from [bis(furan-2-yl)methyl](methyl)amine (Catalog # EN300-2585655, 100 mg, 0.56 mmol) and 5-methyl-1-[3-(trifluoromethyl)phenyl]-1H-pyrazole-3-carboxylic acid (Catalog # EN300-260239, 168 mg, 0.62 mmol).
Yield: 54%; purity, >95% (assessed by LC/MS).
$^1$H NMR (500 MHz, DMSO-$d_6$) δ 7.97 (d, *J* = 7.1 Hz, 1H), 7.94 – 7.86 (m, 2H), 7.79 (dt, *J* = 22.3, 7.8 Hz, 3H), 7.69 (dd, *J* = 14.7, 1.7 Hz, 2H), 6.70 (d, *J* = 5.4 Hz, 1H), 6.51 – 6.41 (m, 2H), 6.37 (d, *J* = 3.3 Hz, 1H), 3.10 (s, 2H), 2.81 (s, 1H), 2.41 (s, 1H), 2.37 (s, 2H).
$^{13}$C NMR (151 MHz, DMSO-$d_6$) δ 163.3, 163.3, 162.1, 150.7, 150.2, 147.5, 147.5, 143.8, 143.7, 141.6, 141.2, 140.8, 140.0, 131.2, 131.0, 130.7, 130.4, 129.2, 128.8, 128.6, 125.2, 125.1, 125.0, 123.2, 121.8, 121.7, 111.1, 111.1, 110.6, 110.3, 110.1, 109.7, 107.9, 53.8, 49.7, 33.3, 30.4, 26.0, 12.5, 12.5, 12.3.
LC/MS (APSI) m/z [M-H] calculated for $C_{22}H_{19}F_3N_3O_3$: 430.1; found: 430.2.

**N,5-dimethyl-N-(2-oxo-1-(thiophen-2-yl)propyl)-1-(3-(trifluoromethyl)phenyl)-1H-pyrazole-3-carboxamide – 1486_44, Z5204106829 (Method 3).**

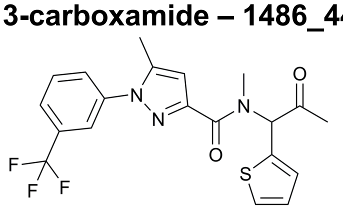

The compound was synthesized from 1-(methylamino)-1-(thiophen-2-yl)propan-2-one hydrochloride (Catalog # EN300-6869532, 100 mg, 0.59 mmol) and 5-methyl-1-[3-

(trifluoromethyl)phenyl]-1H-pyrazole-3-carboxylic acid (Catalog # EN300-260239, 176 mg, 0.65 mmol).
Yield: 22%; purity, >95% (assessed by LC/MS).
$^{1}H$ NMR (500 MHz, DMSO-$d_6$) δ 7.99 – 7.89 (m, 2H), 7.87 – 7.69 (m, 2H), 7.11 (d, J = 3.2 Hz, 1H), 7.08 (d, J = 2.5 Hz, 1H), 7.04 (ddd, J = 12.6, 5.1, 3.5 Hz, 1H), 6.74 – 6.67 (m, 1H), 3.28 (s, 2H), 3.12 (s, 1H), 2.77 (s, 1H), 2.28 (t, J = 2.2 Hz, 1H), 2.14 (d, J = 13.8 Hz, 3H), 1.84 (s, 1H).
$^{13}C$ NMR (151 MHz, DMSO-$d_6$) δ 203.9, 202.2, 164.7, 163.6, 163.2, 150.6, 147.4, 147.3, 147.1, 141.3, 141.0, 140.4, 140.2, 140.0, 139.6, 136.6, 131.2, 131.1, 130.9, 130.5, 129.4, 129.3, 129.1, 128.9, 128.3, 128.1, 127.8, 127.5, 126.8, 126.5, 125.3, 123.8, 121.9, 121.0, 115.3, 110.7, 110.4, 108.9, 66.1, 65.9, 36.4, 36.2, 31.5, 27.7, 27.4, 17.7, 12.6, 12.4, 12.3.
LC/MS (APSI) m/z [M+H] calculated for $C_{20}H_{19}F_3N_3O_2S$: 422.1; found: 422.0.

**2-methyl-4-(5-(2-(4-(trifluoromethyl)phenoxy)propan-2-yl)-1,3,4-oxadiazol-2-yl)phthalazin-1(2H)-one – 0450_21, Z5468612153 (Method 1).**

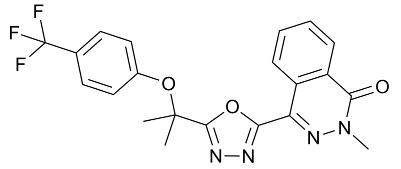

The compound was synthesized from 3-methyl-4-oxo-3,4-dihydrophthalazine-1-carbohydrazide (Catalog # EN300-04897, 97 mg, 0.44 mmol) and 2-methyl-2-[4-(trifluoromethyl)phenoxy]propanoic acid (Catalog # EN300-305386 100 mg, 0.4 mmol).
Yield: 23%; purity, >95% (assessed by LC/MS).
$^{1}H$ NMR (500 MHz, DMSO-$d_6$) δ 8.90 (d, *J* = 8.2 Hz, 1H), 8.36 (dd, *J* = 8.1, 1.4 Hz, 1H), 8.08 – 8.01 (m, 1H), 7.99 – 7.92 (m, 1H), 7.62 (d, *J* = 8.5 Hz, 2H), 7.00 (d, *J* = 8.4 Hz, 2H), 3.82 (s, 3H), 1.88 (s, 6H).
$^{13}C$ NMR (151 MHz, DMSO-$d_6$) δ 168.0, 161.8, 158.8, 157.9, 134.6, 133.0, 130.1, 127.5, 127.4, 127.4, 127.4, 126.9, 126.6, 121.7, 76.1, 26.1.
LC/MS (APSI) m/z [M+H] calculated for $C_{21}H_{18}F_3N_4O_3$: 431.1; found: 431.0.

***rel*-(5-methyl-1-(3-(trifluoromethyl)phenyl)-1H-pyrazol-3-yl)((S)-1-((R)-tetrahydrofuran-2-yl)-2-azaspiro[3.3]heptan-2-yl)methanone – EN300-37364332, Z5889661651 (Method 3).**

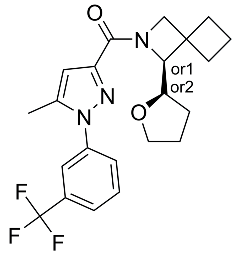

The compound was synthesized from 1-(oxolan-2-yl)-2-azaspiro[3.3]heptane (Catalog # EN300-216770, 100 mg, 0.6 mmol) and 5-methyl-1-[3-(trifluoromethyl)phenyl]-1H-pyrazole-3-carboxylic acid (Catalog # EN300-260239, 178mg, 0.66 mmol) utilizing chiral separation of the mixture.
Yield: 15%; purity, >95% (assessed by LC/MS).
$^{1}H$ NMR (500 MHz, DMSO-$d_6$) δ 7.92 (d, *J* = 9.3 Hz, 2H), 7.84 (d, *J* = 7.8 Hz, 1H), 7.79 (t, *J* = 7.8 Hz, 1H), 6.63 (s, 1H), 4.41 – 4.29 (m, 1H), 4.05 (d, *J* = 3.6 Hz, 2H), 3.82 (d, *J* = 6.9 Hz, 1H), 3.79 (d, *J* = 7.0 Hz, 1H), 3.66 (d, *J* = 7.1 Hz, 2H), 2.36 (s, 3H), 2.07 (d, *J* = 8.1 Hz, 3H), 1.99 (dd, *J* = 13.8, 6.7 Hz, 1H), 1.87 (tq, *J* = 10.9, 5.3 Hz, 3H), 1.82 – 1.69 (m, 2H).
$^{13}C$ NMR (151 MHz, DMSO-$d_6$) δ 163.0, 147.2, 141.0, 140.1, 131.2, 129.2, 125.2, 121.7, 109.3, 79.0, 71.7, 68.0, 65.6, 42.2, 34.2, 29.5, 25.6, 16.5, 12.4.
LC/MS (APSI) m/z [M+H] calculated for $C_{22}H_{25}F_3N_3O_2$: 420.2; found: 420.2.

**_rel_-(5-methyl-1-(3-(trifluoromethyl)phenyl)-1H-pyrazol-3-yl)((R)-1-((R)-tetrahydrofuran-2-yl)-2-azaspiro[3.3]heptan-2-yl)methanone – EN300-37364334, Z5889670430 (Method 3).**

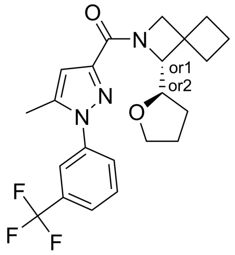


The compound was synthesized from 1-(oxolan-2-yl)-2-azaspiro[3.3]heptane (Catalog # EN300-216770) and 5-methyl-1-[3-(trifluoromethyl)phenyl]-1H-pyrazole-3-carboxylic acid (Catalog # EN300-260239) utilizing chiral separation of the mixture.
Yield: 13%; purity, >95% (assessed by LC/MS).
$^1$H NMR (500 MHz, DMSO-$d_6$) δ 7.92 (d, $J$ = 9.2 Hz, 2H), 7.84 (d, $J$ = 7.9 Hz, 1H), 7.79 (t, $J$ = 7.8 Hz, 1H), 6.63 (s, 1H), 4.39 (d, $J$ = 9.8 Hz, 1H), 4.31 (d, $J$ = 9.8 Hz, 1H), 4.06 (d, $J$ = 3.6 Hz, 2H), 3.80 (q, $J$ = 6.9 Hz, 1H), 3.67 (p, $J$ = 8.9, 7.2 Hz, 1H), 2.47 – 2.37 (m, 1H), 2.36 (s, 3H), 2.12 – 2.04 (m, 2H), 1.99 (dd, $J$ = 13.6, 6.3 Hz, 1H), 1.88 (qd, $J$ = 11.4, 10.4, 6.0 Hz, 3H), 1.78 (s, 1H), 1.83 – 1.69 (m, 2H).
$^{13}$C NMR (151 MHz, DMSO-$d_6$) δ 163.0, 147.2, 141.0, 140.1, 131.2, 129.2, 125.1 (d, $J$ = 32.8 Hz), 121.6, 109.3, 79.0, 71.7, 68.0, 65.6, 42.2, 34.2, 29.5, 25.6, 16.5, 12.4.
LC/MS (APSI) m/z [M+H] calculated for $C_{22}H_{25}F_3N_3O_2$: 420.2; found: 420.2.

**_rel_-(5-methyl-1-(3-(trifluoromethyl)phenyl)-1H-pyrazol-3-yl)((R)-1-((S)-tetrahydrofuran-2-yl)-2-azaspiro[3.3]heptan-2-yl)methanone – EN300-37364335, Z5889673386 (Method 3).**

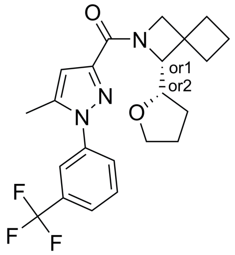


The compound was synthesized from 1-(oxolan-2-yl)-2-azaspiro[3.3]heptane (Catalog # EN300-216770) and 5-methyl-1-[3-(trifluoromethyl)phenyl]-1H-pyrazole-3-carboxylic acid (Catalog # EN300-260239) utilizing chiral separation of the mixture.
Yield: 10%; purity, >95% (assessed by LC/MS).
$^1$H NMR (500 MHz, DMSO-$d_6$) δ 7.92 (d, $J$ = 9.9 Hz, 2H), 7.84 (d, $J$ = 7.8 Hz, 1H), 7.79 (t, $J$ = 7.8 Hz, 1H), 6.63 (s, 1H), 4.34 (q, $J$ = 9.5 Hz, 2H), 4.28 – 4.24 (m, 1H), 4.16 (td, $J$ = 7.3, 2.4 Hz, 1H), 3.67 (dq, $J$ = 32.2, 7.3 Hz, 2H), 2.36 (s, 3H), 2.28 (dt, $J$ = 11.8, 8.5 Hz, 1H), 2.15 – 2.03 (m, 3H), 1.90 (ddq, $J$ = 18.3, 12.9, 7.3, 5.9 Hz, 2H), 1.73 (q, $J$ = 7.1 Hz, 3H), 1.72 – 1.64 (m, 1H).
$^{13}$C NMR (151 MHz, DMSO-$d_6$) δ 162.7, 147.3, 141.0, 140.1, 131.2, 129.2, 125.2, 121.6, 109.3, 78.5, 72.6, 68.4, 66.4, 42.6, 34.5, 28.9, 25.8, 16.3, 12.4.
LC/MS (APSI) m/z [M+H] calculated for $C_{22}H_{25}F_3N_3O_2$: 420.2; found: 420.2.

**rel-(5-methyl-1-(3-(trifluoromethyl)phenyl)-1H-pyrazol-3-yl)((S)-1-((S)-tetrahydrofuran-2-yl)-2-azaspiro[3.3]heptan-2-yl)methanone – EN300-37364336, Z5889675490 (Method 3).**

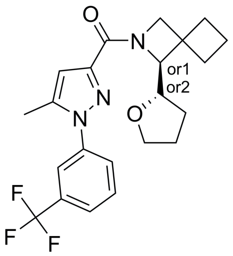


The compound was synthesized from 1-(oxolan-2-yl)-2-azaspiro[3.3]heptane (Catalog # EN300-216770) and 5-methyl-1-[3-(trifluoromethyl)phenyl]-1H-pyrazole-3-carboxylic acid (Catalog # EN300-260239) utilizing chiral separation of the mixture.
Yield: 11%; purity, >95% (assessed by LC/MS).
$^{1}$H NMR (500 MHz, DMSO-$d_6$) δ 7.92 (d, *J* = 9.6 Hz, 2H), 7.84 (d, *J* = 7.9 Hz, 1H), 7.79 (t, *J* = 7.8 Hz, 1H), 6.63 (s, 1H), 4.36 (d, *J* = 9.5 Hz, 1H), 4.32 (d, *J* = 9.5 Hz, 1H), 4.28 – 4.24 (m, 1H), 4.16 (td, *J* = 7.3, 2.4 Hz, 1H), 3.67 (dq, *J* = 32.3, 7.3 Hz, 2H), 2.48 (p, *J* = 1.9 Hz, 3H), 2.28 (d, *J* = 11.4 Hz, 1H), 2.13 – 2.05 (m, 3H), 1.90 (dq, *J* = 13.4, 5.5, 4.5 Hz, 2H), 1.72 (dq, *J* = 12.8, 5.8, 4.7 Hz, 4H).
$^{13}$C NMR (151 MHz, DMSO-$d_6$) δ 162.7, 147.3, 141.0, 140.1, 131.2, 129.2, 125.2, 121.6, 109.3, 78.5, 72.6, 68.4, 66.4, 42.6, 34.5, 28.9, 28.6, 25.8, 16.3, 12.4.
LC/MS (APSI) m/z [M+H] calculated for $C_{22}H_{25}F_3N_3O_2$: 420.2; found: 420.2.

**4-(5-(2-(4-fluorophenoxy)propan-2-yl)-1,3,4-oxadiazol-2-yl)-2-methylphthalazin-1(2H)-one – 31486, Z685931486 (Method 1).**

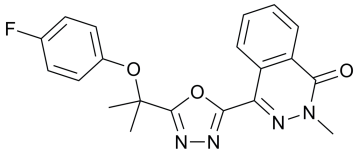


The compound was synthesized from 3-methyl-4-oxo-3,4-dihydrophthalazine-1-carbohydrazide (Catalog # EN300-04897, 121 mg,0.56 mmol) and 2-(4-fluorophenoxy)-2-methylpropanoic acid (Catalog # EN300-11485, 100 mg, 0.51 mmol).
Yield: 14%; purity, >95% (assessed by LC/MS).
$^{1}$H NMR (400 MHz, DMSO-$d_6$) δ 8.86 (d, *J* = 8.2 Hz, 1H), 8.31 (d, *J* = 7.9 Hz, 1H), 8.01 (t, *J* = 7.8 Hz, 1H), 7.92 (t, *J* = 7.7 Hz, 1H), 7.08 (t, *J* = 8.8 Hz, 2H), 6.87 – 6.79 (m, 2H), 1.81 (s, 9H).
$^{13}$C NMR (101 MHz, DMSO-$d_6$) δ 168.1, 161.7, 158.7, 158.0, 150.6, 134.5, 132.9, 130.1, 127.3, 126.8, 126.6, 124.6, 116.6, 76.0, 40.3, 25.9.
LC/MS (APSI) m/z [M+H] calculated for $C_{20}H_{18}FN_4O_3$: 381.1; found: 381.0.

**4-(5-(2-(4-chlorophenoxy)propan-2-yl)-1,3,4-oxadiazol-2-yl)-2-isopropylphthalazin-1(2H)-one – 41360, Z685941360 (Method 1).**

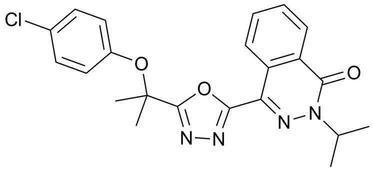


The compound was synthesized from 4-oxo-3-(propan-2-yl)-3,4-dihydrophthalazine-1-carbohydrazide (Catalog # EN300-05133, 126 mg, 0.51 mmol) and 2-(4-chlorophenoxy)-2-methylpropanoic acid (Catalog # EN300-18267, 100 mg, 0.47 mmol).
Yield: 28%; purity, >95% (assessed by LC/MS).
$^{1}$H NMR (500 MHz, DMSO-$d_6$) δ 8.83 (s, 1H), 8.39 – 8.35 (m, 1H), 8.04 (s, 1H), 7.95 (s, 1H), 7.31 – 7.26 (m, 2H), 6.90 – 6.84 (m, 2H), 5.31 (s, 1H), 2.49 (s, 2H), 1.85 (s, 5H), 1.37 (s, 5H).

$^{13}C$ NMR (101 MHz, DMSO-$d_6$) δ 168.0, 161.9, 158.1, 153.5, 134.7, 132.9, 130.2, 129.8, 128.7, 127.4, 127.2, 126.8, 126.4, 124.5, 76.3, 49.7, 26.0, 21.3.
LC/MS (APSI) m/z [M+H] calculated for $C_{22}H_{22}ClN_4O_3$: 425.1; found: 425.0.

**methyl 2-(N,5-dimethyl-1-(3-(trifluoromethyl)phenyl)-1H-pyrazole-3-carboxamido)-2-(furan-2-yl)acetate – 1486_46, Z5422676425 (Method 3).**

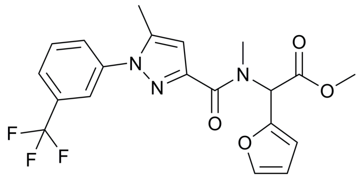

The compound was synthesized from methyl 2-(furan-2-yl)-2-(methylamino)acetate (Catalog # EN300-37160511, 100 mg, 0.59 mmol) and 5-methyl-1-[3-(trifluoromethyl)phenyl]-1H-pyrazole-3-carboxylic acid (Catalog # EN300-260239, 176 mg, 0.65 mmol).
Yield: 27%; purity, >95% (assessed by LC/MS).
$^1H$ NMR (500 MHz, DMSO-$d_6$) δ 8.54 (s, 1H), 7.99 (d, *J* = 2.1 Hz, 1H), 7.95 (dd, *J* = 7.8, 2.2 Hz, 1H), 7.87 (d, *J* = 7.8 Hz, 1H), 7.80 (t, *J* = 7.9 Hz, 1H), 7.39 (d, *J* = 5.1 Hz, 1H), 7.07 (d, *J* = 3.5 Hz, 1H), 6.94 (dd, *J* = 5.1, 3.5 Hz, 1H), 6.67 (s, 1H), 2.35 (s, 3H), 1.96 (s, 3H).
$^{13}C$ NMR (151 MHz, DMSO-$d_6$) δ 169.4, 168.7, 163.9, 148.2, 147.7, 147.3, 144.4, 144.2, 141.0, 139.9, 131.2, 131.2, 131.1, 129.2, 128.5, 125.3, 125.1, 124.8, 121.8, 121.5, 111.7, 111.3, 110.9, 110.4, 108.9, 59.0, 56.0, 53.1, 52.9, 49.5, 35.5, 34.7, 31.3, 21.5, 20.5, 15.1, 12.5, 12.3.
LC/MS (APSI) m/z [M+H] calculated for $C_{20}H_{19}F_3N_3O_4$: 422.1; found: 422.2.

**methyl 2-(5-phenyl-1-(3-(trifluoromethyl)phenyl)-1H-pyrazole-3-carboxamido)-2-(thiophen-2-yl)acetate – 1486_71, Z6314888079 (Method 3).**

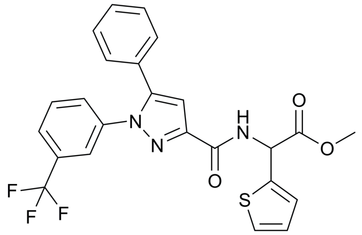

The compound was synthesized from methyl 2-amino-2-(thiophen-2-yl)acetate hydrochloride (Catalog # EN300-60031, 100 mg, 0.48 mmol) and 5-phenyl-1-[3-(trifluoromethyl)phenyl]-1H-pyrazole-3-carboxylic acid (Catalog # EN300-37430088, 175 mg, 0.53 mmol).
Yield: 33%; purity, >95% (assessed by LC/MS).
$^1H$ NMR (500 MHz, DMSO-$d_6$) δ 9.10 (d, *J* = 7.5 Hz, 1H), 7.78 (d, *J* = 7.7 Hz, 1H), 7.75 (d, *J* = 2.1 Hz, 1H), 7.65 (t, *J* = 7.9 Hz, 1H), 7.60 (d, *J* = 8.4 Hz, 1H), 7.50 (dd, *J* = 5.1, 1.3 Hz, 1H), 7.43 – 7.34 (m, 3H), 7.28 (dq, *J* = 4.7, 2.9 Hz, 2H), 7.17 (d, *J* = 3.6 Hz, 1H), 7.11 (s, 1H), 7.00 (dd, *J* = 5.1, 3.5 Hz, 1H), 5.91 (d, *J* = 7.5 Hz, 1H), 3.69 (s, 3H).
$^{13}C$ NMR (126 MHz, DMSO-$d_6$) δ 169.9, 160.5, 146.6, 144.7, 139.6, 138.4, 130.4, 129.3, 129.0, 128.8, 128.8, 127.2, 126.6, 126.4, 122.1, 108.4, 52.6, 51.5.
LC/MS (APSI) m/z [M+H] calculated for $C_{24}H_{19}F_3N_3O_3S$: 486.1; found: 486.0.

**(1-(furan-2-yl)-2-azaspiro[3.3]heptan-2-yl)(5-methyl-1-(3-(trifluoromethyl)phenyl)-1H-pyrazol-3-yl)methanone – 10_10, Z6314892034 (Method 3).**

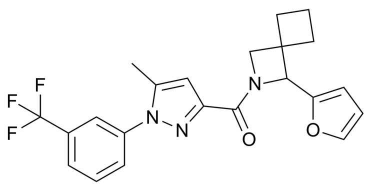

The compound was synthesized from 1-(furan-2-yl)-2-azaspiro[3.3]heptane (Catalog # EN300-244752, 100 mg, 0.61 mmol) and 5-methyl-1-[3-(trifluoromethyl)phenyl]-1H-pyrazole-3-carboxylic acid (Catalog # EN300-260239, 182 mg, 0.67 mmol).
Yield: 26%; purity, 92% (assessed by LC/MS).
$^{1}$H NMR (600 MHz, DMSO-$d_6$) δ 8.05 (t, *J* = 6.0 Hz, 1H), 7.95 (s, 1H), 7.92 (d, *J* = 8.0 Hz, 1H), 7.85 (d, *J* = 7.9 Hz, 1H), 7.79 (t, *J* = 7.9 Hz, 1H), 7.55 (d, *J* = 1.8 Hz, 1H), 6.67 (s, 1H), 6.37 (dd, *J* = 3.2, 1.7 Hz, 1H), 6.30 (d, *J* = 3.2 Hz, 1H), 5.67 (d, *J* = 4.7 Hz, 1H), 4.62 (d, *J* = 4.7 Hz, 1H), 3.45 (dd, *J* = 13.6, 6.7 Hz, 1H), 2.36 (s, 3H), 2.06 – 1.91 (m, 2H), 1.84 – 1.80 (m, 1H), 1.81 – 1.73 (m, 1H), 1.61 (tq, *J* = 9.9, 4.6 Hz, 1H).
$^{13}$C NMR (151 MHz, DMSO-$d_6$) δ 162.0, 156.4, 147.7, 142.2, 141.8, 140.0, 131.1, 129.1, 125.3, 122.0, 110.5, 108.0, 107.2, 72.2, 46.0, 43.8, 26.6, 25.8, 14.9, 12.5.
LC/MS (APSI) m/z [M+H] calculated for $C_{22}H_{21}F_3N_3O_2$: 416.2; found: 416.0.

**(5-methyl-1-(3-(trifluoromethyl)phenyl)-1H-pyrazol-3-yl)((3a'S,6a'R)-tetrahydrospiro[cyclobutane-1,3'-furo[3,4-b]pyrrol]-1'(2'H)-yl)methanone – 10_14, Z6314892089 (Method 3).**

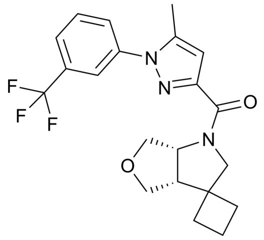

The compound was synthesized from rac-(3'aR,6'aS)-hexahydrospiro[cyclobutane-1,3'-furo[3,4-b]pyrrole] hydrochloride (Catalog # EN300-1425927, 100 mg, 0.53 mmol) and 5-methyl-1-[3-(trifluoromethyl)phenyl]-1H-pyrazole-3-carboxylic acid (Catalog # EN300-260239, 157 mg, 0.58 mmol).
Yield: 31%; purity, >95% (assessed by LC/MS).
$^{1}$H NMR (600 MHz, DMSO-$d_6$) δ 7.97 – 7.89 (m, 2H), 7.82 (dt, *J* = 21.6, 8.0 Hz, 2H), 6.68 (d, *J* = 20.5 Hz, 1H), 5.07 (t, *J* = 5.5 Hz, 1H), 4.58 (dd, *J* = 7.4, 4.2 Hz, 1H), 4.28 (d, *J* = 11.5 Hz, 1H), 3.80 (dd, *J* = 9.9, 3.8 Hz, 1H), 3.71 (dddd, *J* = 25.7, 16.8, 9.8, 4.4 Hz, 2H), 3.59 – 3.50 (m, 2H), 2.87 (q, *J* = 7.2 Hz, 1H), 2.81 (td, *J* = 7.8, 5.1 Hz, 1H), 2.38 (d, *J* = 3.4 Hz, 3H), 2.01 – 1.83 (m, 2H), 1.83 – 1.71 (m, 2H).
$^{13}$C NMR (151 MHz, DMSO-$d_6$) δ 161.0, 148.4, 140.9, 140.8, 140.1, 131.2, 130.4, 129.1, 128.8, 125.2, 121.7, 121.5, 110.5, 109.9, 77.2, 74.6, 69.8, 69.4, 63.6, 62.5, 58.6, 56.2, 53.5, 51.0, 47.0, 45.0, 40.5, 34.9, 34.7, 26.2, 26.0, 16.1, 15.9, 12.4.
LC/MS (APSI) m/z [M+H] calculated for $C_{21}H_{23}F_3N_3O_2$: 406.2; found: 406.0.

**3-((6-bromobenzo[d][1,3]dioxol-5-yl)methyl)-5-(2-((tetrahydrofuran-3-yl)oxy)pyridin-4-yl)-1,2,4-oxadiazole – 7019_11, Z1684296820 (Method 5).**

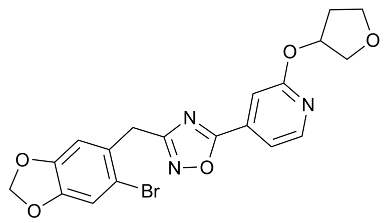

The compound was synthesized from 2-(6-bromo-1,3-dioxaindan-5-yl)acetonitrile (Catalog # EN300-53536, 100 mg, 0.42 mmol) and 2-(oxolan-3-yloxy)pyridine-4-carboxylic acid (Catalog # EN300-122851, 96 mg, 0.46 mmol).
Yield: 27%; purity, >95% (assessed by LC/MS).
$^1$H NMR (500 MHz, DMSO-$d_6$) δ 8.39 (d, $J$ = 5.3 Hz, 1H), 7.56 – 7.51 (m, 1H), 7.31 (s, 1H), 7.21 (d, $J$ = 1.6 Hz, 1H), 7.06 (d, $J$ = 1.5 Hz, 1H), 6.06 (s, 2H), 5.57 – 5.51 (m, 1H), 3.91 (dd, $J$ = 10.4, 4.6 Hz, 1H), 3.85 (q, $J$ = 7.9 Hz, 1H), 3.76 (td, $J$ = 12.9, 11.8, 7.6 Hz, 2H), 3.32 (s, 1H), 2.53 (d, $J$ = 1.5 Hz, 1H), 2.29 – 2.18 (m, 1H), 2.01 (dt, $J$ = 12.8, 5.7 Hz, 1H).
$^{13}$C NMR (126 MHz, DMSO-$d_6$) δ 173.2, 169.4, 163.3, 148.8, 147.5, 147.2, 133.2, 127.6, 114.4, 112.3, 111.0, 109.2, 102.0, 76.4, 72.4, 66.3, 32.4, 32.0.
LC/MS (APSI) m/z [M+H] calculated for $C_{19}H_{17}BrN_3O_5$: 446.0; found: 446.0.

**3-((6-bromobenzo[d][1,3]dioxol-5-yl)methyl)-5-(1-(cyclopropylmethyl)-1H-pyrazol-4-yl)-1,2,4-oxadiazole – 7019_31, Z5722895424 (Method 5).**

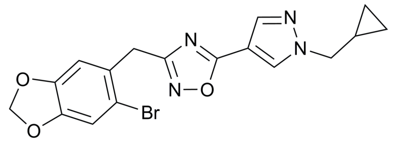

The compound was synthesized from 2-(6-bromo-1,3-dioxaindan-5-yl)acetonitrile (Catalog # EN300-53536, 100 mg, 0.42 mmol) and 1-(cyclopropylmethyl)-1H-pyrazole-4-carboxylic acid (Catalog # EN300-305583, 76 mg, 0.46 mmol).
Yield: 31%; purity, >95% (assessed by LC/MS).
$^1$H NMR (400 MHz, DMSO-$d_6$) δ 8.51 (s, 1H), 7.93 (s, 1H), 7.05 (s, 1H), 6.89 (s, 1H), 6.03 (s, 2H), 4.08 (s, 2H), 4.05 (d, $J$ = 7.2 Hz, 2H), 1.28 (d, $J$ = 31.5 Hz, 1H), 0.59 (d, $J$ = 7.5 Hz, 2H), 0.44 (d, $J$ = 5.2 Hz, 2H).
$^{13}$C NMR (126 MHz, DMSO-$d_6$) δ 170.7, 168.6, 147.5, 147.2, 138.6, 131.7, 128.0, 114.3, 112.3, 111.0, 106.2, 102.0, 56.1, 32.0, 11.3, 3.5.
LC/MS (APSI) m/z [M+H] calculated for $C_{17}H_{16}BrN_4O_3$: 405.0; found: 405.0.

**(2,3-dihydrobenzo[b][1,4]oxathiin-2-yl)methyl 1-(4-chlorobenzyl)-3,5-dimethyl-1H-pyrazole-4-carboxylate – 7800_29, Z5722895463 (Method 2).**

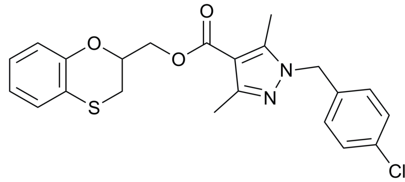

The compound was synthesized from (2,3-dihydro-1,4-benzoxathiin-2-yl)methanol (Catalog # EN300-59707, 100 mg, 0.55 mmol) and 1-[(4-chlorophenyl)methyl]-3,5-dimethyl-1H-pyrazole-4-carboxylic acid (Catalog # EN300-99633, 145 mg, 0.6 mmol).
Yield: 19%; purity, >95% (assessed by LC/MS).
$^1$H NMR (500 MHz, DMSO-$d_6$) δ 7.42 – 7.36 (m, 2H), 7.34 (dd, $J$ = 7.6, 1.7 Hz, 1H), 7.19 (td, $J$ = 7.7, 1.7 Hz, 1H), 7.14 (d, $J$ = 8.4 Hz, 2H), 7.00 (ddt, $J$ = 10.7, 7.4, 1.5 Hz, 2H), 5.34 (q, $J$ = 3.6 Hz, 1H), 5.28 (s, 2H), 4.39 (dd, $J$ = 13.2, 3.0 Hz, 1H), 4.27 (dd, $J$ = 13.0, 4.6 Hz, 1H), 3.22 – 3.14 (m, 1H), 2.45 (s, 4H), 2.32 (s, 3H).

LC/MS (APSI) m/z [M+H] calculated for $C_{22}H_{22}ClN_2O_3S$: 429.1; found: 429.0.

**6-(3-(difluoro(4-fluorophenyl)methyl)-1,2,4-oxadiazol-5-yl)-2-(4-fluorophenyl)pyridazin-3(2H)-one – 2443, Z1684426368 (Method 5).**

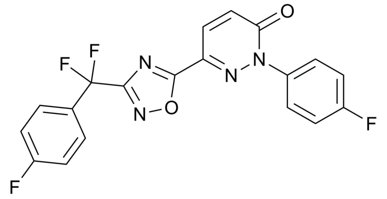

The compound was synthesized from 2,2-difluoro-2-(4-fluorophenyl)acetonitrile (Catalog # EN300-55799, 100 mg, 0.58 mmol) and 1-(4-fluorophenyl)-6-oxo-1,6-dihydropyridazine-3-carboxylic acid (Catalog # EN300-36469, 151 mg, 0.64 mmol).
Yield: 33%; purity, >95% (assessed by LC/MS).
$^1$H NMR (400 MHz, DMSO-$d_6$) δ 8.10 (d, $J$ = 9.9 Hz, 1H), 7.70 (dt, $J$ = 21.8, 7.5 Hz, 4H), 7.32 (s, 1H), 7.27 (dd, $J$ = 20.9, 8.9 Hz, 4H).
$^{13}$C NMR (151 MHz, DMSO-$d_6$) δ 173.3, 166.9, 165.1, 163.4, 163.0, 161.4, 159.1, 137.5, 132.4, 131.7, 129.5, 128.9, 128.6, 116.7.
LC/MS (APSI) m/z [M+H] calculated for $C_{19}H_{11}F_4N_4O_2$: 403.1; found: 403.0.

**N-(furan-2-yl(3-methoxyphenyl)methyl)-5-methyl-1-(3-(trifluoromethyl)phenyl)-1H-pyrazole-3-carboxamide – 1486_38, Z4967431087 (Method 3).**

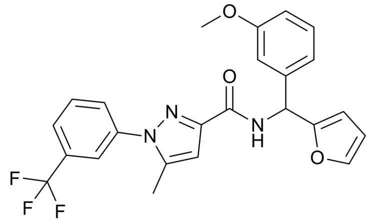

The compound was synthesized from (furan-2-yl)(3-methoxyphenyl)methanamine (Catalog # EN300-2209969, 100 mg, 0.49 mmol) and 5-methyl-1-[3-(trifluoromethyl)phenyl]-1H-pyrazole-3-carboxylic acid (Catalog # EN300-260239, 146 mg, 0.54 mmol).
Yield: 34%; purity, >95% (assessed by LC/MS).
$^1$H NMR (400 MHz, Chloroform-$d$) δ 7.74 (d, $J$ = 21.2 Hz, 2H), 7.67 (d, $J$ = 6.5 Hz, 2H), 7.66 – 7.56 (m, 1H), 7.40 (d, $J$ = 1.8 Hz, 1H), 7.28 (d, $J$ = 15.8 Hz, 1H), 6.98 (d, $J$ = 7.7 Hz, 1H), 6.93 (t, $J$ = 2.2 Hz, 1H), 6.88 – 6.78 (m, 2H), 6.48 (d, $J$ = 8.6 Hz, 1H), 6.37 – 6.31 (m, 1H), 6.24 (d, $J$ = 3.2 Hz, 1H), 3.80 (s, 3H), 2.39 (s, 3H).
$^{13}$C NMR (151 MHz, DMSO-$d_6$) δ 161.1, 159.7, 154.6, 147.2, 143.0, 141.9, 140.0, 131.1, 129.9, 129.2, 125.3, 122.1, 120.2, 113.9, 113.2, 110.9, 108.3, 108.1, 55.5, 50.6, 12.5.
LC/MS (APSI) m/z [M+H] calculated for $C_{24}H_{21}F_3N_3O_3$: 456.2; found: 456.2.

**2-(N,5-dimethyl-1-(3-(trifluoromethyl)phenyl)-1H-pyrazole-3-carboxamido)-2-(thiophen-2-yl)acetic acid – 9056, Z5003379056 (Method 3).**

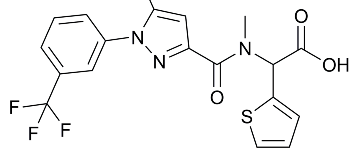

The compound was synthesized from 2-(methylamino)-2-(thiophen-2-yl)acetic acid (Catalog # EN300-1259865, 100 mg, 0.58 mmol) and 5-methyl-1-[3-(trifluoromethyl)phenyl]-1H-pyrazole-3-carboxylic acid (Catalog # EN300-260239, 174 mg,0.64 mmol).

Yield: 35%; purity, >95% (assessed by LC/MS).
$^{1}$H NMR (500 MHz, DMSO-$d_6$) δ 13.27 (s, 1H), 8.54 (s, 1H), 7.99 (d, *J* = 2.1 Hz, 1H), 7.95 (dd, *J* = 7.8, 2.1 Hz, 1H), 7.87 (d, *J* = 7.8 Hz, 1H), 7.80 (t, *J* = 7.9 Hz, 1H), 7.39 (d, *J* = 5.0 Hz, 1H), 7.07 (d, *J* = 3.6 Hz, 1H), 6.94 (dd, *J* = 5.2, 3.5 Hz, 1H), 6.67 (s, 1H), 2.35 (s, 3H), 1.96 (s, 3H).
$^{13}$C NMR (151 MHz, DMSO-$d_6$) δ 160.5, 147.3, 142.1, 139.9, 131.2, 129.5, 126.9, 125.7, 125.5, 122.2, 107.9, 25.0, 12.4.
LC/MS (APSI) m/z [M+H] calculated for $C_{19}H_{17}F_3N_3O_3S$: 424.1; found: 424.0.

**methyl 2-(5-methyl-1-(3-(trifluoromethyl)phenyl)-1H-pyrazole-3-carboxamido)-2-(thiophen-2-yl)propanoate – 4042, Z8504214042 (Method 3).**

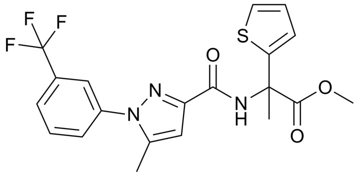


The compound was synthesized from methyl 2-amino-2-(thiophen-2-yl)propanoate hydrochloride (Catalog # EN300-46816185, 100 mg, 0.45 mmol) and 5-methyl-1-[3-(trifluoromethyl)phenyl]-1H-pyrazole-3-carboxylic acid (Catalog # EN300-260239, 134 mg, 0.5 mmol).
Yield: 30%; purity, >95% (assessed by LC/MS).
$^{1}$H NMR (600 MHz, DMSO-$d_6$) δ 8.65 (s, 1H), 7.99 (d, *J* = 2.3 Hz, 1H), 7.95 (d, *J* = 7.9 Hz, 1H), 7.86 (d, *J* = 7.8 Hz, 1H), 7.80 (t, *J* = 7.9 Hz, 1H), 7.45 (dd, *J* = 5.1, 1.2 Hz, 1H), 7.14 (dd, *J* = 3.6, 1.3 Hz, 1H), 6.96 (dd, *J* = 5.1, 3.6 Hz, 1H), 6.71 (s, 1H), 3.62 (s, 3H), 2.36 (s, 3H), 1.92 (s, 3H).
$^{13}$C NMR (151 MHz, DMSO-$d_6$) δ 172.3, 161.0, 146.8, 145.2, 142.0, 139.9, 131.1, 130.7, 130.5, 129.3, 125.0, 123.2, 122.2 (2), 108.2, 59.5, 53.1, 40.2, 25.7, 12.5.
LC/MS (APSI) m/z [M+H] calculated for $C_{20}H_{19}F_3N_3O_3S$: 438.1; found: 438.2.

**methyl (R)-2-(5-methyl-1-(3-(trifluoromethyl)phenyl)-1H-pyrazole-3-carboxamido)-2-(thiophen-2-yl)propanoate – 1350, Z8526711350.**

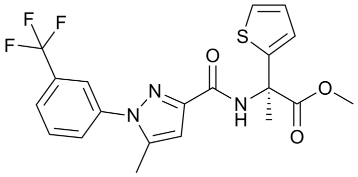


The compound was obtained by a chiral separation of Z8504214042.
Yield: 15%; purity, >95% (assessed by LC/MS).
$[\alpha]^{21}_{D}$ = -29.1 (*c* 1.0, $CH_3OH$).
$^{1}$H NMR (500 MHz, DMSO-$d_6$) δ 8.66 (s, 1H), 7.99 (d, *J* = 2.0 Hz, 1H), 7.95 (d, *J* = 8.4 Hz, 1H), 7.86 (d, *J* = 7.7 Hz, 1H), 7.79 (t, *J* = 7.9 Hz, 1H), 7.45 (dd, *J* = 5.1, 1.3 Hz, 1H), 7.14 (dd, *J* = 3.6, 1.3 Hz, 1H), 6.96 (dd, *J* = 5.1, 3.6 Hz, 1H), 6.70 (s, 1H), 3.62 (s, 3H), 2.36 (s, 3H), 1.91 (s, 3H).
LC/MS (APSI) m/z [M+H] calculated for $C_{20}H_{19}F_3N_3O_3S$: 438.1; found: 438.2.

**methyl (S)-2-(5-methyl-1-(3-(trifluoromethyl)phenyl)-1H-pyrazole-3-carboxamido)-2-(thiophen-2-yl)propanoate – 8690, Z8526708690.**

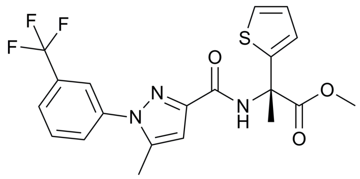

The compound was obtained by a chiral separation of Z8504214042.
Yield: 12%; purity, >95% (assessed by LC/MS).
$[\alpha]^{21}_D = 27.7$ (*c* 2.0, $CH_3OH$).
$^1$H NMR (500 MHz, DMSO-$d_6$) δ 8.66 (s, 1H), 7.99 (d, *J* = 2.1 Hz, 1H), 7.95 (d, *J* = 8.1 Hz, 1H), 7.86 (d, *J* = 7.8 Hz, 1H), 7.79 (t, *J* = 7.9 Hz, 1H), 7.45 (dd, *J* = 5.1, 1.3 Hz, 1H), 7.14 (dd, *J* = 3.6, 1.3 Hz, 1H), 6.96 (dd, *J* = 5.1, 3.6 Hz, 1H), 6.70 (s, 1H), 3.62 (s, 3H), 2.36 (s, 3H), 1.92 (s, 3H).
LC/MS (APSI) m/z [M+H] calculated for $C_{20}H_{19}F_3N_3O_3S$: 438.1; found: 438.2.

**N-(1,1-di(furan-2-yl)ethyl)-5-methyl-1-(3-(trifluoromethyl)phenyl)-1H-pyrazole-3-carboxamide – Z8703004936 (Method 3).**

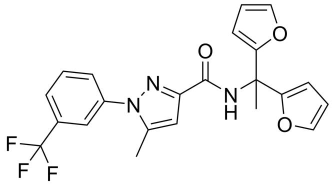

The compound was synthesized from 1,1-bis(furan-2-yl)ethan-1-amine (Catalog # EN300-46161469, 100 mg, 0.56 mmol) and 5-methyl-1-[3-(trifluoromethyl)phenyl]-1H-pyrazole-3-carboxylic acid (Catalog # EN300-260239, 168 mg, 0.62 mmol).
Yield: 28%; purity, >95% (assessed by LC/MS).
$^1$H NMR (500 MHz, DMSO-$d_6$) δ 7.98 (s, 1H), 7.97 – 7.91 (m, 2H), 7.85 (d, *J* = 7.8 Hz, 1H), 7.79 (t, *J* = 7.9 Hz, 1H), 7.57 (d, *J* = 1.9 Hz, 2H), 6.65 (s, 1H), 6.39 (dd, *J* = 3.2, 1.8 Hz, 2H), 6.22 (d, *J* = 3.3 Hz, 2H), 2.34 (s, 3H), 2.05 (s, 3H).
$^{13}$C NMR (151 MHz, DMSO-$d_6$) δ 160.5, 155.5, 147.4, 142.6, 142.1, 139.9, 131.2, 130.7, 129.5, 125.5, 123.2, 122.2, 110.9, 107.9, 106.7, 54.5, 24.2, 12.4.
LC/MS (APSI) m/z [2M+Na] calculated for C44H36F6N6Na1O6: 881.3; found: 881.2.

**Spectral Data for ‘4042, its enantiomers, and ‘4936.**

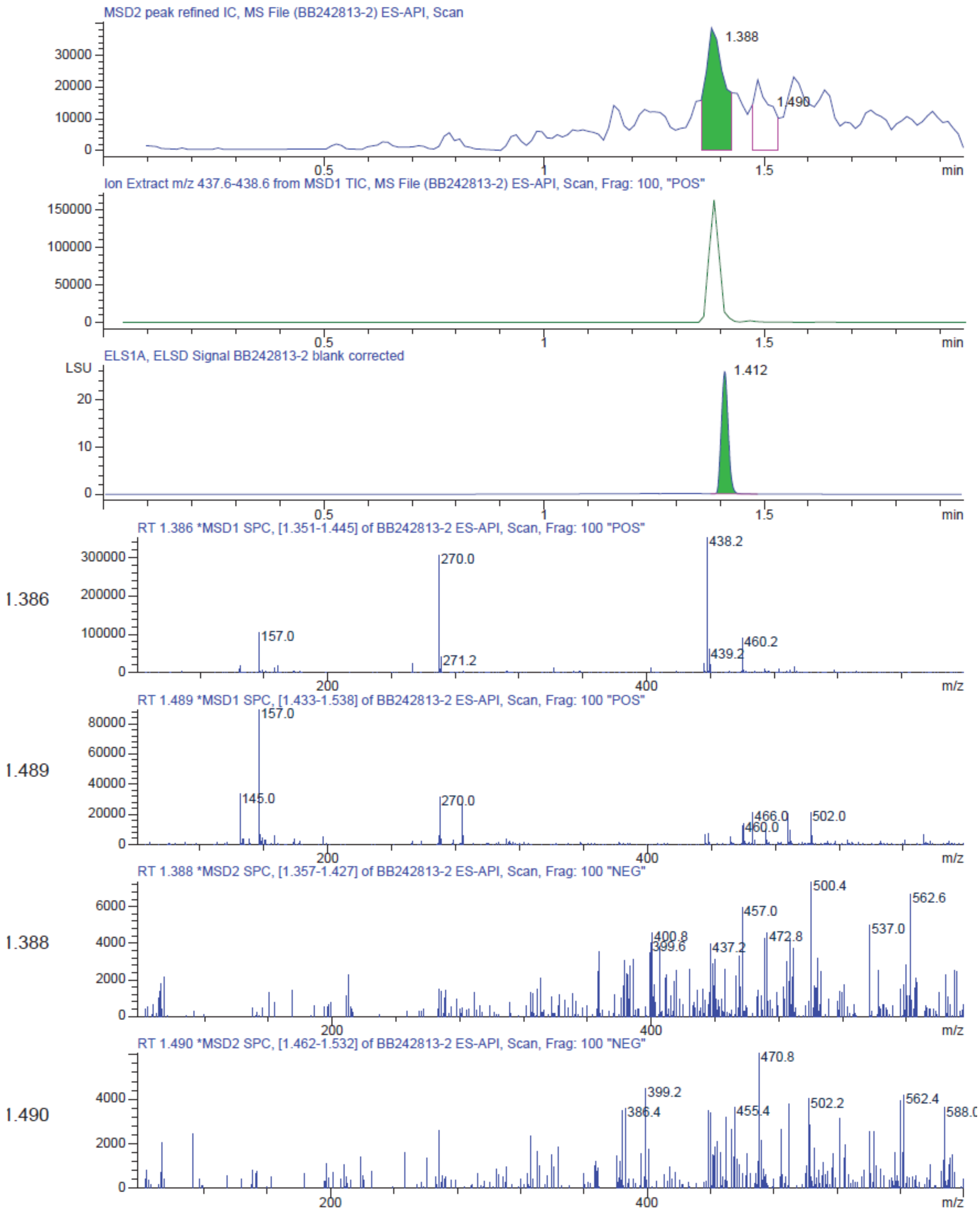


Z8504214042

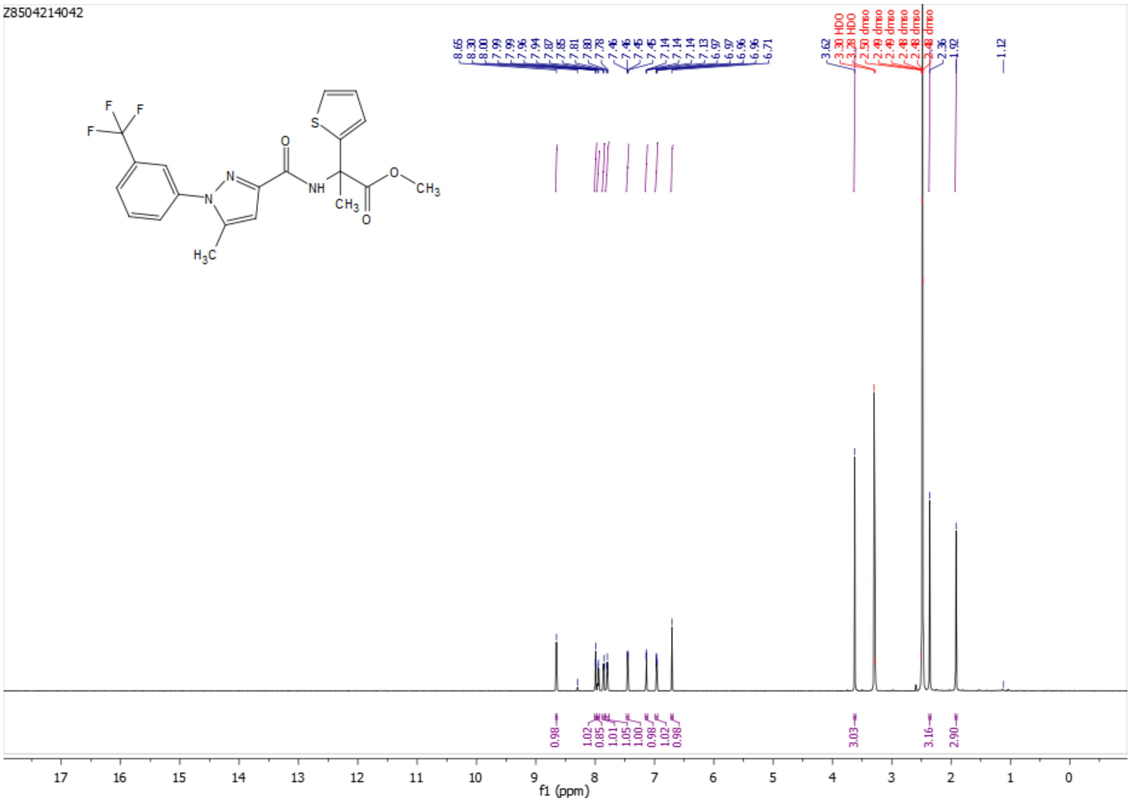


Z8504214042

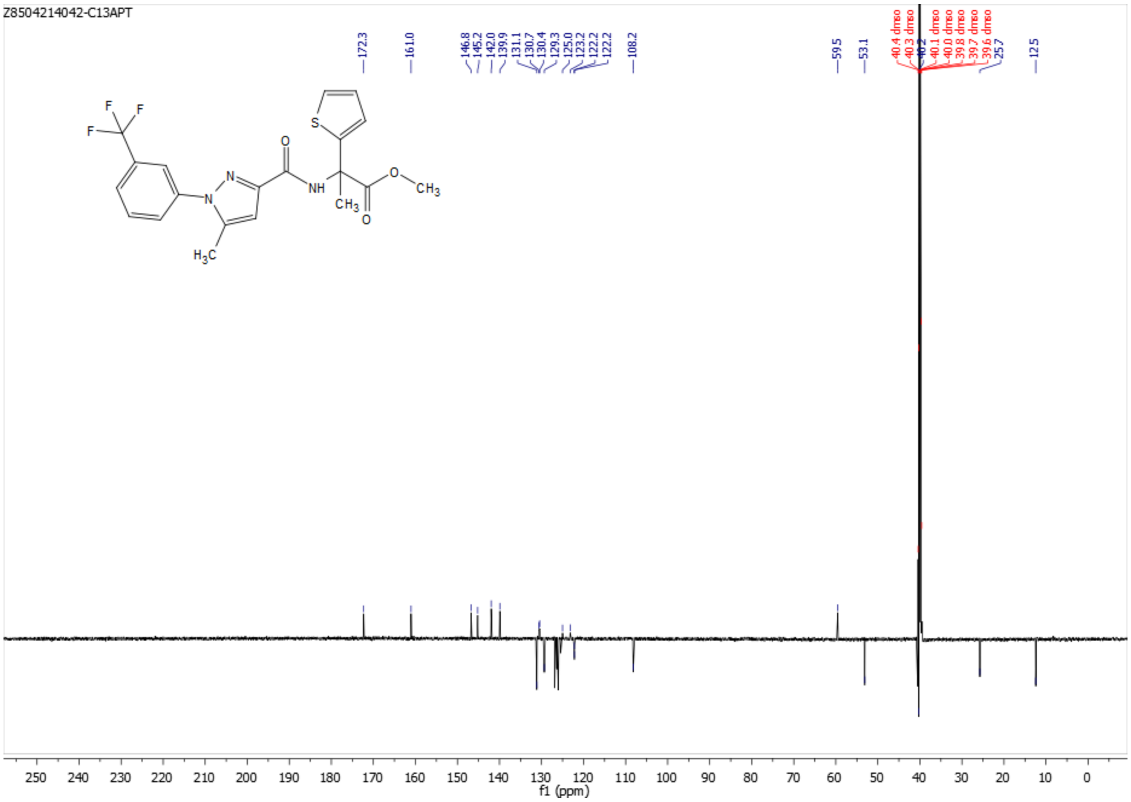


Z8504214042

**Compound Table**

| Label | Tgt Score | Mass Error (ppm) | Tgt Formula | Obs. RT | Ref. Mass | Obs. Mass |
|---|---|---|---|---|---|---|
| Cpd 2: C20 H18 F3 N3 O3 S; 3.105 | 98.89 | -0.92 | C20 H18 F3 N3 O3 S | 3.105 | 437.1021 | 437.10169 |

| Obs. *m/z* | Obs. RT | Obs. Mass | Tgt Formula | Tgt Mass | Tgt Mass Error (ppm) | RT Diff. | Find Cpds Algorithm |
|---|---|---|---|---|---|---|---|
| 460.09092 | 3.105 | 437.10169 | C20 H18 F3 N3 O3 S | 437.1021 | -0.92 | Find By Formula | |

**Compound Chromatograms**

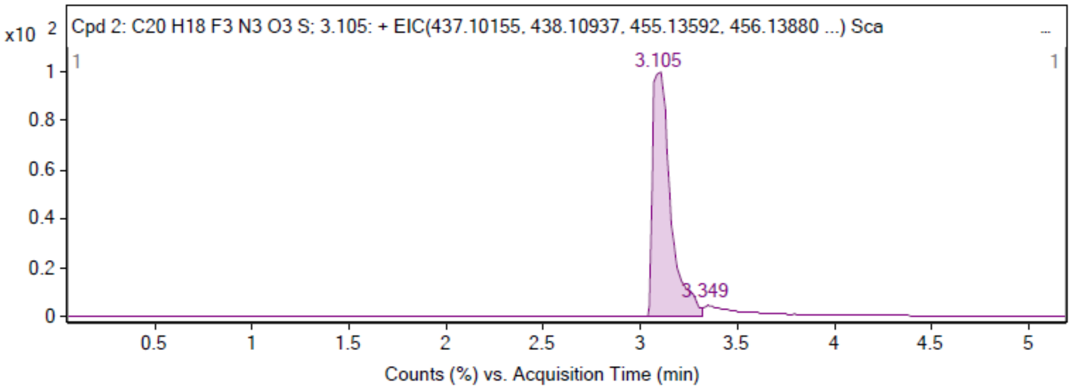

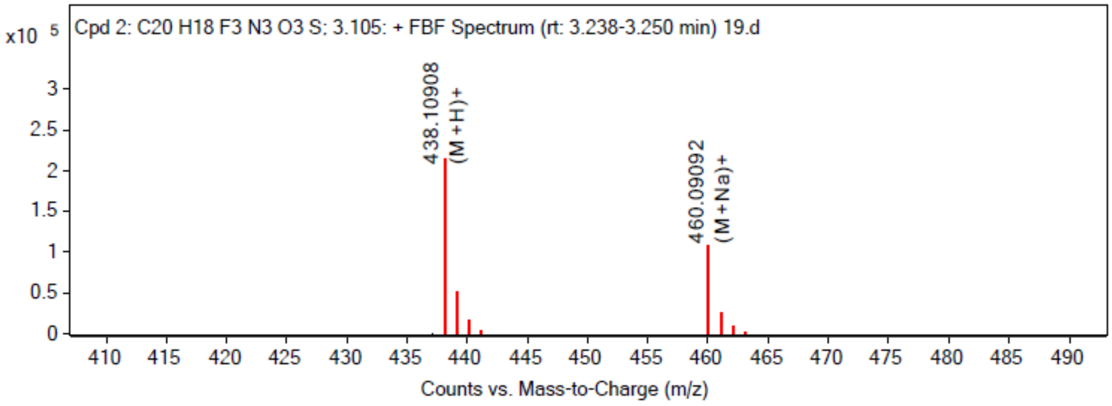


**MS Spectrum Peak List**

| Obs. *m/z* | Charge | Abund | Ion/Isotope |
|---|---|---|---|
| 437.09646 | 1 | 721.56 | M+ |
| 438.10908 | 1 | 214832.16 | (M+H)+ |
| 439.11167 | 1 | 44635.38 | (M+H)+ |
| 440.10898 | 1 | 14234.8 | (M+H)+ |
| 441.10958 | 1 | 2337.3 | (M+H)+ |
| 460.09092 | 1 | 107890.51 | (M+Na)+ |
| 461.09352 | 1 | 24119.89 | (M+Na)+ |
| 462.0906 | 1 | 7645.8 | (M+Na)+ |
| 463.09444 | 1 | 1390.56 | (M+Na)+ |

MS Zoomed Spectrum

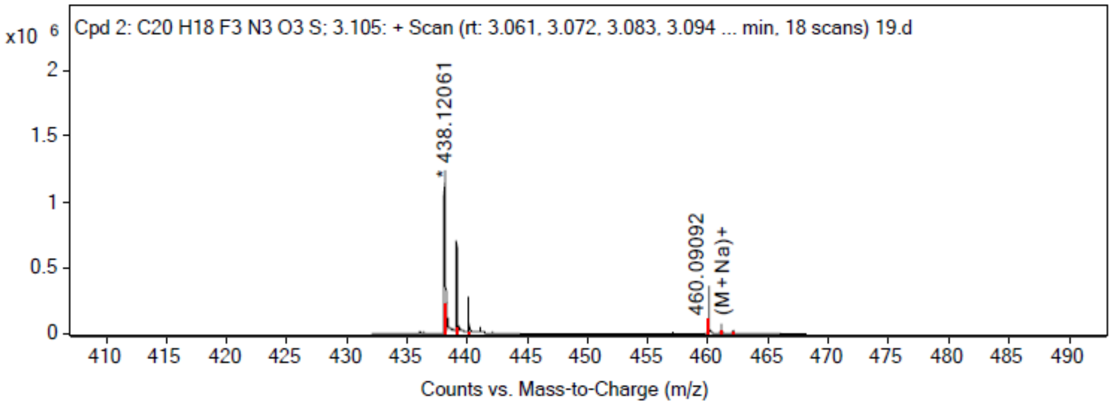


**MS Spectrum Peak List**

| Obs. *m/z* | Charge | Abund | Ion/Isotope | Tgt Mass Error (ppm) |
|---|---|---|---|---|
| 437.09646 | 1 | 721.56 | M+ | 11.65 |
| 438.10908 | 1 | 214832.16 | (M+H)+ | 0.67 |
| 438.12061 | | 1248557.22 | | |
| 439.11167 | 1 | 44635.38 | (M+H)+ | 1.52 |
| 440.10898 | 1 | 14234.8 | (M+H)+ | 0.85 |
| 441.10958 | 1 | 2337.3 | (M+H)+ | 2.26 |
| 460.09092 | 1 | 107890.51 | (M+Na)+ | 0.86 |
| 461.09352 | 1 | 24119.89 | (M+Na)+ | 1.65 |
| 462.0906 | 1 | 7645.8 | (M+Na)+ | 1.53 |
| 463.09444 | 1 | 1390.56 | (M+Na)+ | -4.15 |

--- End Of Report ---

Z8504214042

Data File: C:\USERS\PUBLIC\DOCUMENTS\CHEMSTATION\1\DATA\2023-05-12_NIGHT\2023-05-12 2023-05-12 17-42-28\BB338254$1_8199_RA->
Sample Name: BB338254$1_8199_RAC

Acq. Operator : SYSTEM Location: 91
Acq. Instrument : HPLC_07
Injection Date : 6:14:56 PM 5/12/2023
Injection Volume: 5 mkl
Column: Chiralpak IC (250x4.6 mm, 5 mkm)--833VJ002-DA181--7
Mobile Phase: Hexane:MeOH:IPA, 70:15:15
Flow Rate: 0.6 ml/min

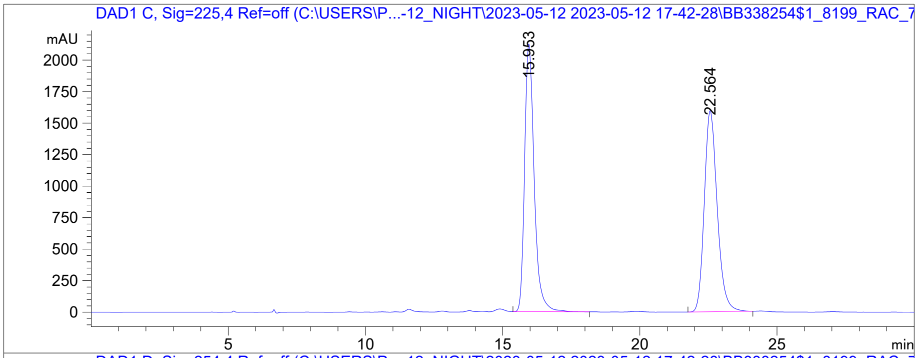


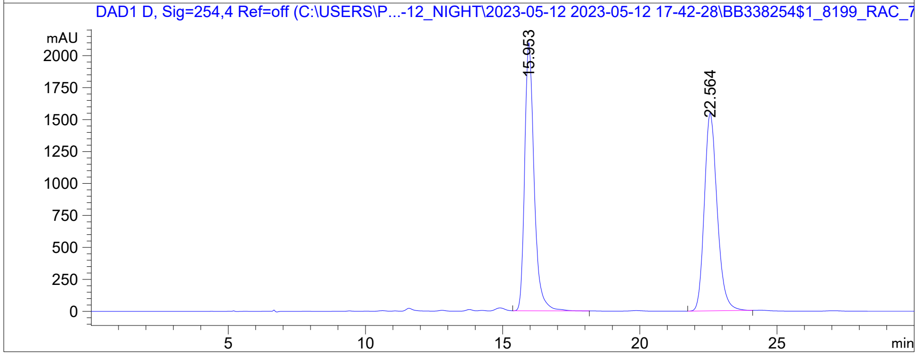


Signal: DAD1 C, Sig=225,4 Ref=off

| RetTime(min) | Area,% | Area | Resolution | Selectivity | 30.000 |
|---|---|---|---|---|---|
| 15.9532 | 49.67 | 51534 | | | |
| 22.5642 | 50.33 | 52220 | 9.08 | 1.62 | |

Signal: DAD1 D, Sig=254,4 Ref=off

| RetTime(min) | Area,% | Area | Resolution | Selectivity | 30.000 |
|---|---|---|---|---|---|
| 15.9532 | 49.88 | 50153 | | | |
| 22.5642 | 50.12 | 50389 | 9.17 | 1.62 | |



Z8504214042

MaxPeak: 100.00%
Ret_Time: 4.019 min

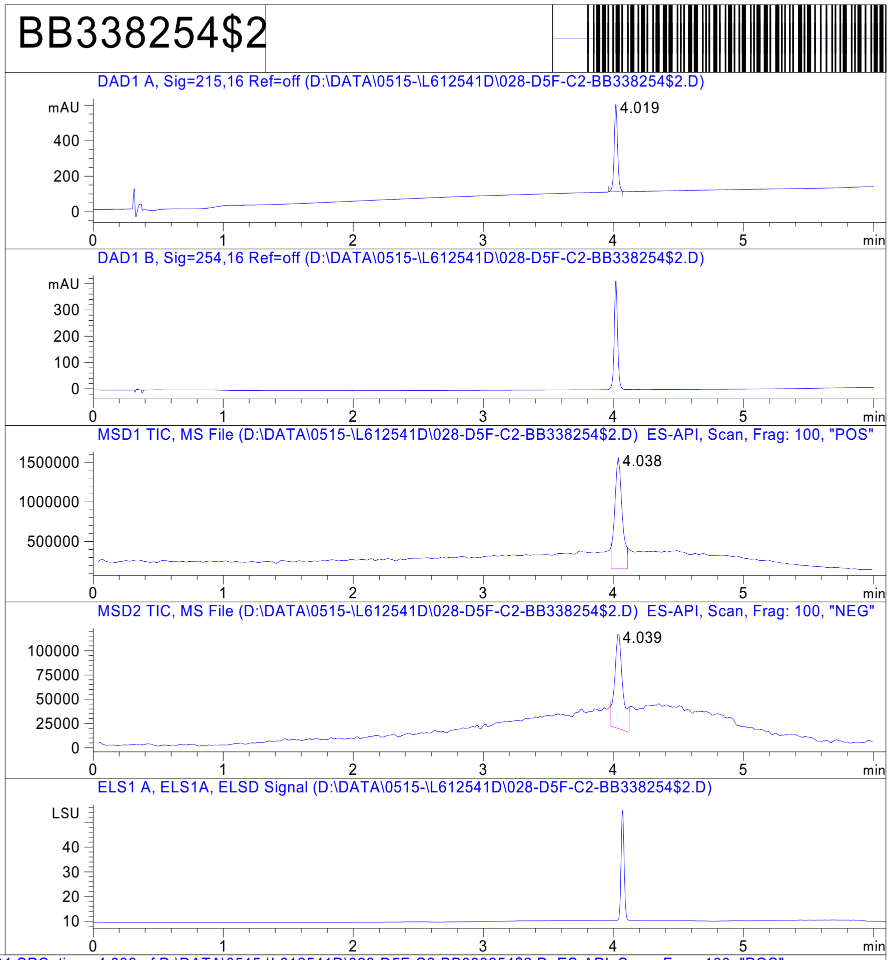


**Mol Wt** 437.44

**Exact Mass** 437.12

| # | Time | Area% |
|---|---|---|
| 1 | 4.019 | 100.00 |

RT 4.038

RT 4.039

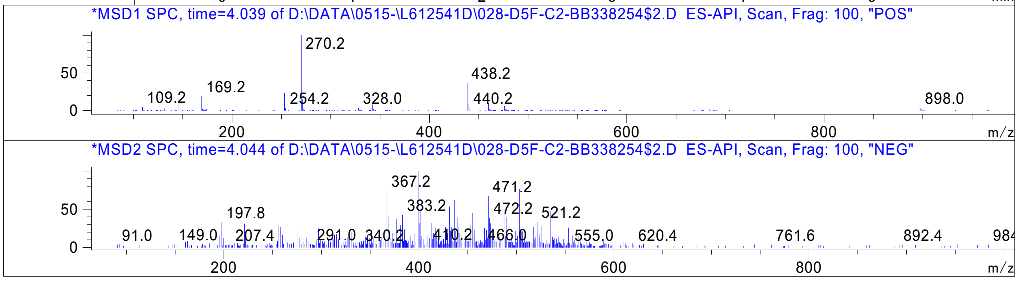


Inj.Date 5/15/2023 H -26- Acq. Method C:\Users\ -> ->

Z8526711350

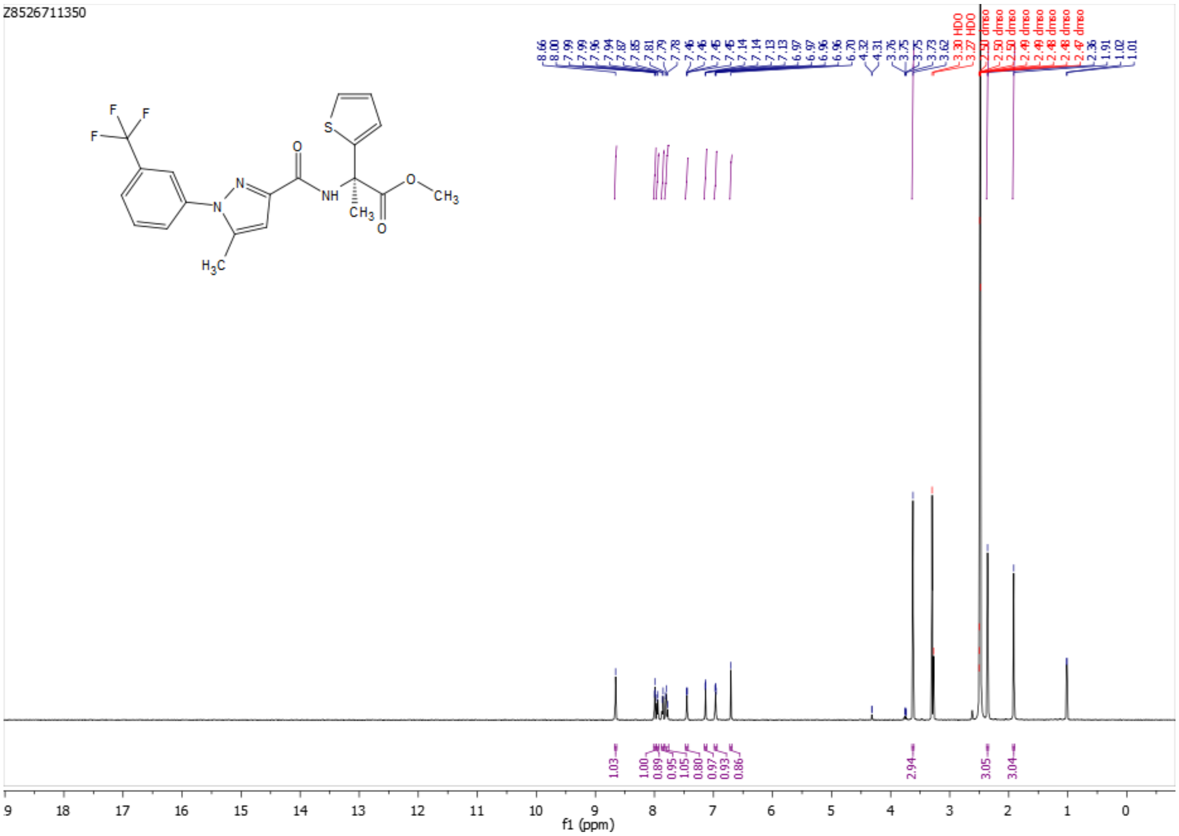


Z8526711350

**Compound Table**

| Label | Tgt Score | Mass Error (ppm) | Tgt Formula | Obs. RT | Ref. Mass | Obs. Mass |
|---|---|---|---|---|---|---|
| Cpd 3: C20 H18 F3 N3 O3 S; 3.129 | 98.44 | -1.13 | C20 H18 F3 N3 O3 S | 3.129 | 437.1021 | 437.1016 |

| Obs. *m/z* | Obs. RT | Obs. Mass | Tgt Formula | Tgt Mass | Tgt Mass Error (ppm) | RT Diff. | Find Cpds Algorithm |
|---|---|---|---|---|---|---|---|
| 438.10883 | 3.129 | 437.1016 | C20 H18 F3 N3 O3 S | 437.1021 | -1.13 | Find By Formula | |

**Compound Chromatograms**

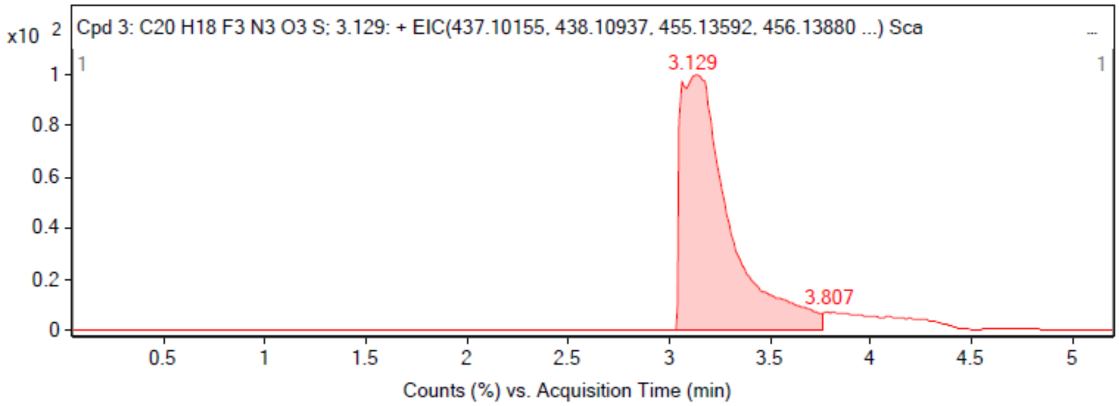

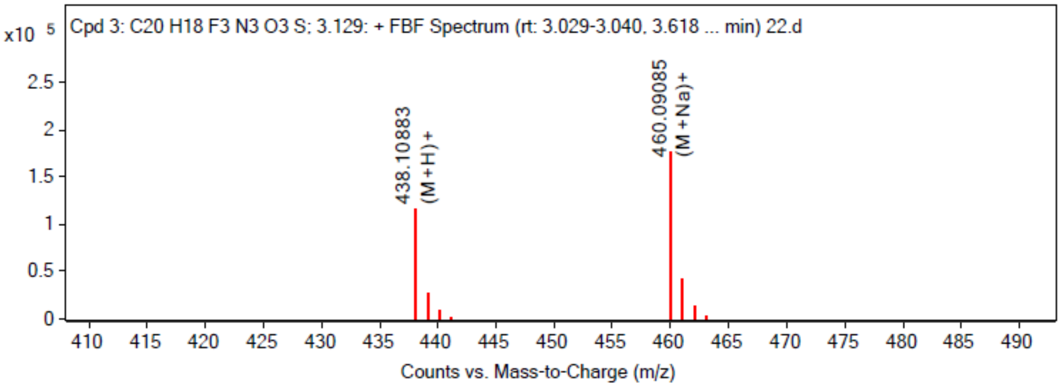


**MS Spectrum Peak List**

| Obs. *m/z* | Charge | Abund | Ion/Isotope |
|---|---|---|---|
| 438.10883 | 1 | 117409.48 | (M+H)+ |
| 439.11148 | 1 | 26160.09 | (M+H)+ |
| 440.1095 | 1 | 8223.04 | (M+H)+ |
| 441.10948 | 1 | 1344.91 | (M+H)+ |
| 460.09085 | 1 | 177480.36 | (M+Na)+ |
| 461.09388 | 1 | 37935.23 | (M+Na)+ |
| 462.09065 | 1 | 11314.79 | (M+Na)+ |
| 463.09462 | 1 | 1967.56 | (M+Na)+ |

MS Zoomed Spectrum

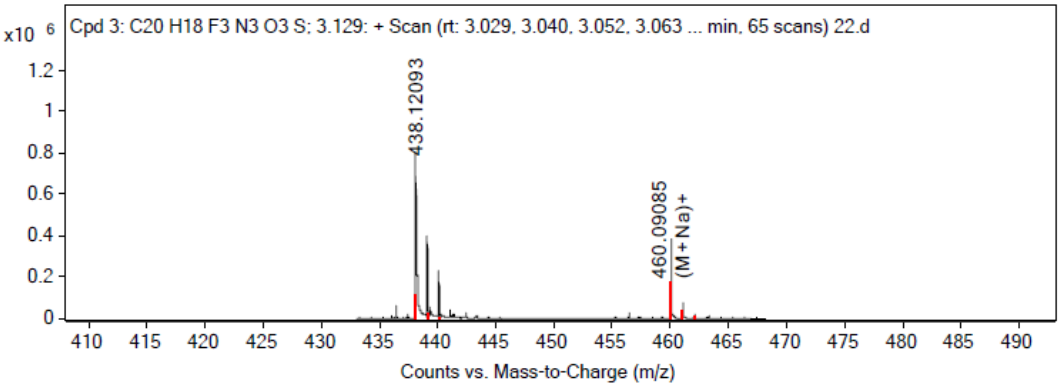


**MS Spectrum Peak List**

| Obs. *m/z* | Charge | Abund | Ion/Isotope | Tgt Mass Error (ppm) |
|---|---|---|---|---|
| 438.10883 | 1 | 117409.48 | (M+H)+ | 1.24 |
| 438.12093 | | 817354.68 | | |
| 439.11148 | 1 | 26160.09 | (M+H)+ | 1.94 |
| 440.1095 | 1 | 8223.04 | (M+H)+ | -0.32 |
| 441.10948 | 1 | 1344.91 | (M+H)+ | 2.48 |
| 460.09085 | 1 | 177480.36 | (M+Na)+ | 1.02 |
| 461.09388 | 1 | 37935.23 | (M+Na)+ | 0.88 |
| 462.09065 | 1 | 11314.79 | (M+Na)+ | 1.41 |
| 463.09462 | 1 | 1967.56 | (M+Na)+ | -4.55 |

--- End Of Report ---

Z8526711350

Data File: C:\USERS\PUBLIC\DOCUMENTS\CHEMSTATION\1\DATA\2023-05-12_NIGHT\2023-05-12 2023-05-12 17-42-28\BB338254$2_8199_F2->
Sample Name: BB338254$2_8199_F2

Acq. Operator : SYSTEM Location: 93
Acq. Instrument : HPLC_07
Injection Date : 7:17:16 PM 5/12/2023
Injection Volume: 5 mkl
Column: Chiralpak IC (250x4.6 mm, 5 mkm)--833VJ002-DA181--7
Mobile Phase: Hexane:MeOH:IPA, 70:15:15
Flow Rate: 0.6 ml/min

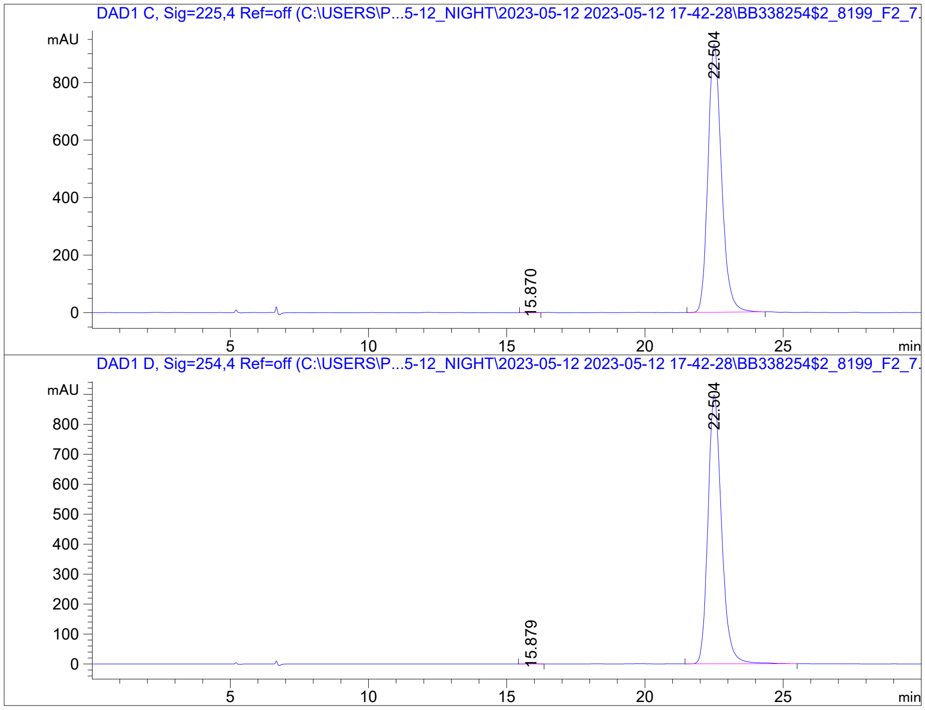


Signal: DAD1 C, Sig=225,4 Ref=off

| RetTime(min) | Area,% | Area | Resolution | Selectivity | 29.997 |
|---|---|---|---|---|---|
| 15.8702 | 0.11 | 35 | | | |
| 22.5042 | 99.89 | 31614 | 9.14 | 1.63 | |

Signal: DAD1 D, Sig=254,4 Ref=off

| RetTime(min) | Area,% | Area | Resolution | Selectivity | 29.997 |
|---|---|---|---|---|---|
| 15.8792 | 0.12 | 37 | | | |
| 22.5042 | 99.88 | 30708 | 9.02 | 1.63 | |



Z8526711350

MaxPeak: 100.00%
Ret_Time: 4.022 min

# BB338254$1

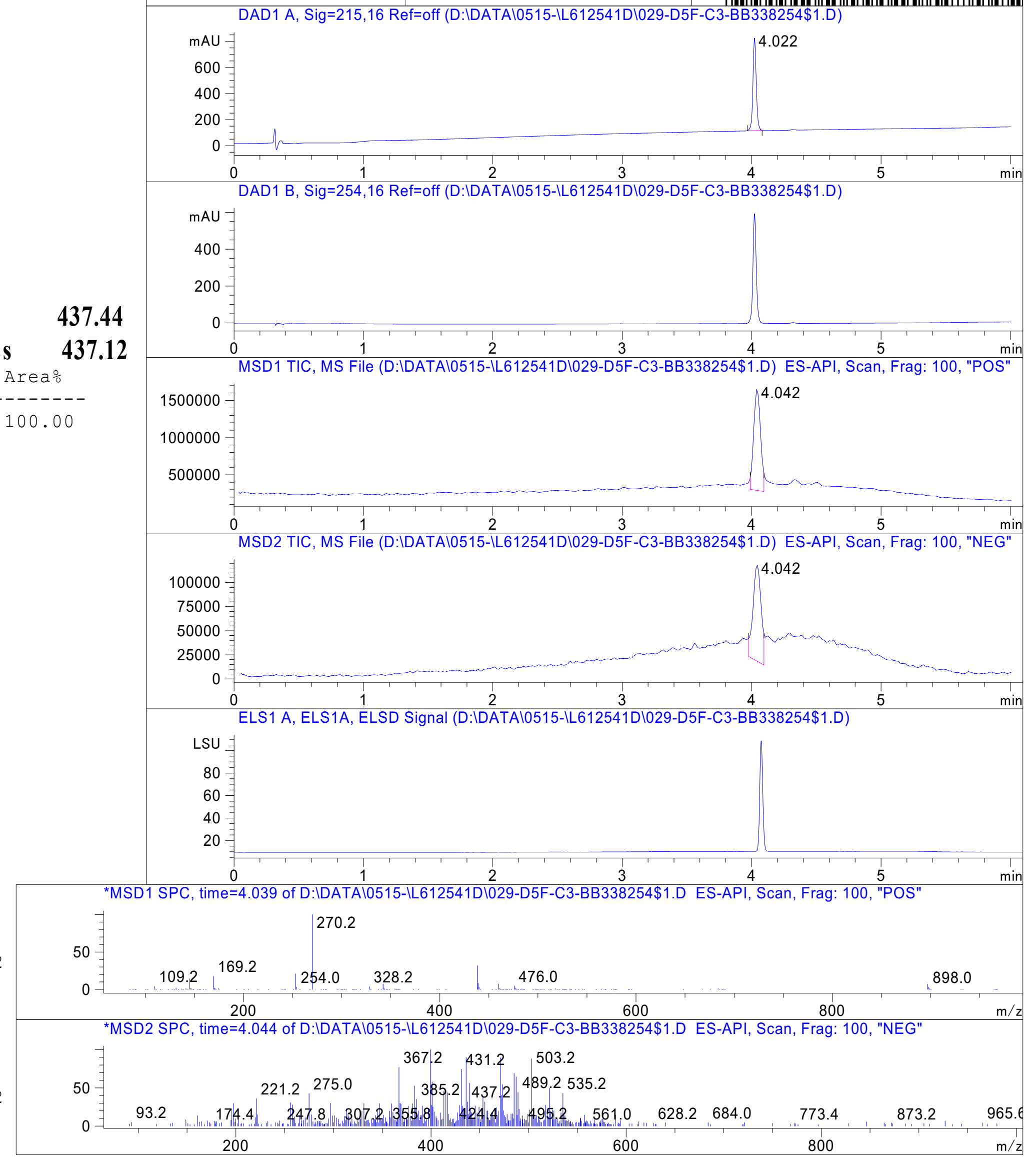


**Mol Wt** 437.44
**Exact Mass** 437.12

| # | Time | Area% |
|---|---|---|
| 1 | 4.022 | 100.00 |

RT 4.042

RT 4.042

Inj.Date 5/15/2023 H  Acq. Method C:\Users\ -> ->

Z8526708690

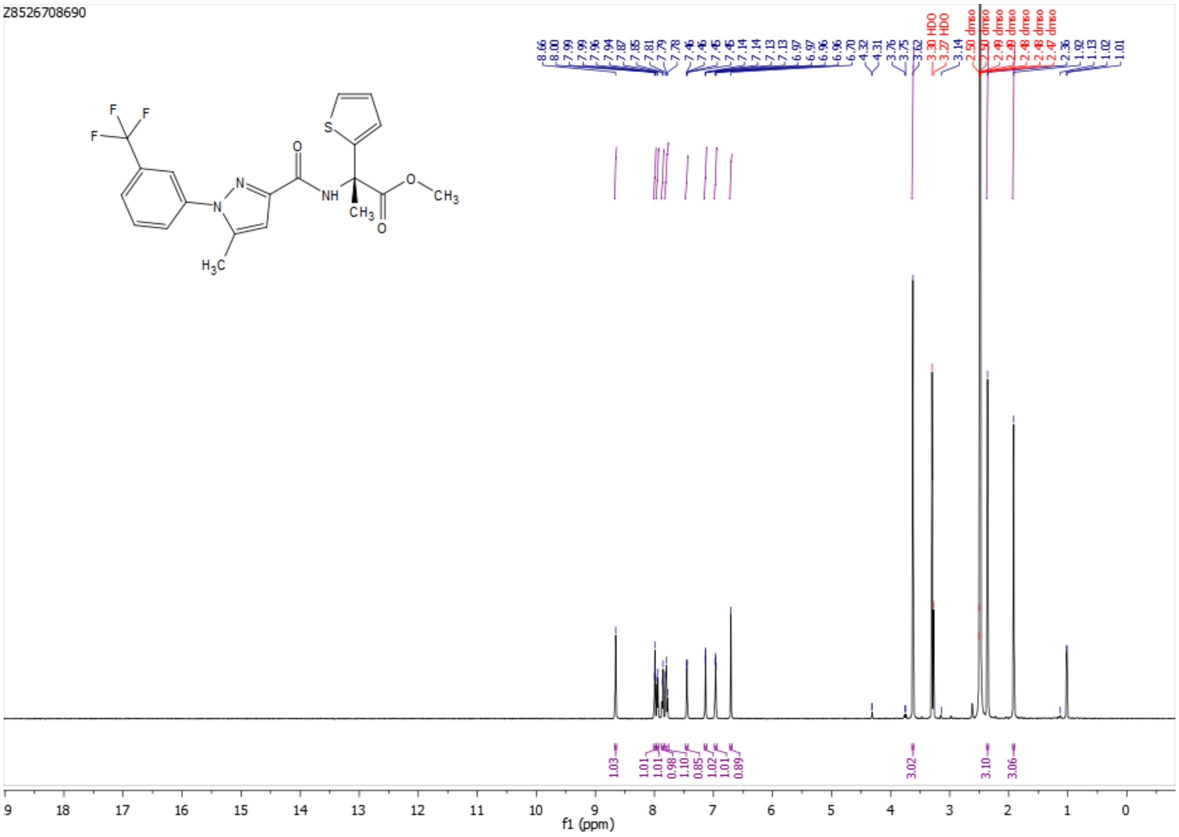


Z8526708690

**Compound Table**

| Label | Tgt Score | Mass Error (ppm) | Tgt Formula | Obs. RT | Ref. Mass | Obs. Mass |
|---|---|---|---|---|---|---|
| Cpd 2: C20 H18 F3 N3 O3 S; 3.112 | 86.33 | 2.54 | C20 H18 F3 N3 O3 S | 3.112 | 437.1021 | 437.10321 |

| Obs. *m/z* | Obs. RT | Obs. Mass | Tgt Formula | Tgt Mass | Tgt Mass Error (ppm) | RT Diff. | Find Cpds Algorithm |
|---|---|---|---|---|---|---|---|
| 438.10908 | 3.112 | 437.10321 | C20 H18 F3 N3 O3 S | 437.1021 | 2.54 | Find By Formula | |

**Compound Chromatograms**

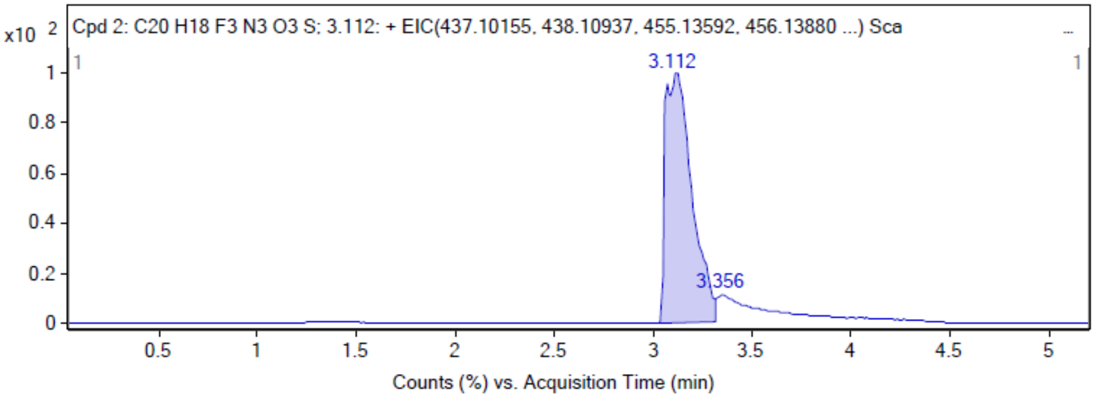

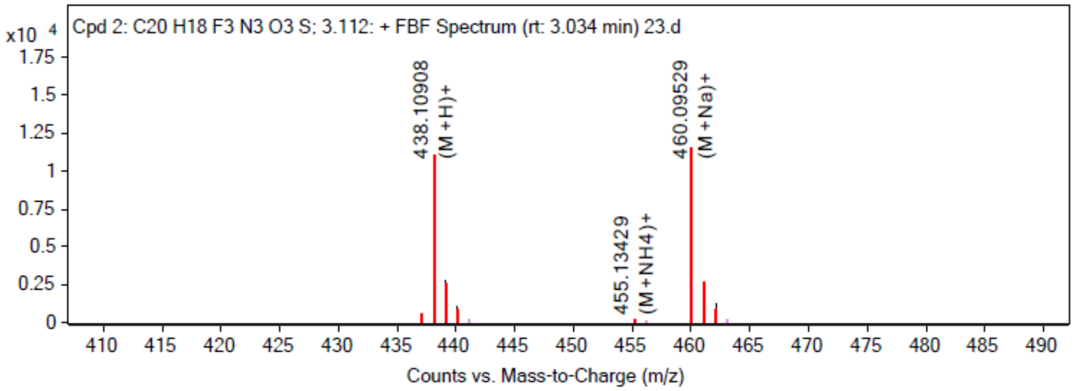


**MS Spectrum Peak List**

| Obs. *m/z* | Charge | Abund | Ion/Isotope |
|---|---|---|---|
| 437.09596 | 1 | 614.12 | M+ |
| 438.10908 | 1 | 10678.29 | (M+H)+ |
| 439.11323 | 1 | 2842.31 | (M+H)+ |
| 440.10169 | 1 | 1031.5 | (M+H)+ |
| 455.13429 | 1 | 229.03 | (M+NH4)+ |
| 460.09529 | 1 | 11240.28 | (M+Na)+ |
| 461.0982 | 1 | 2619.27 | (M+Na)+ |
| 462.08445 | 1 | 1270.68 | (M+Na)+ |

MS Zoomed Spectrum

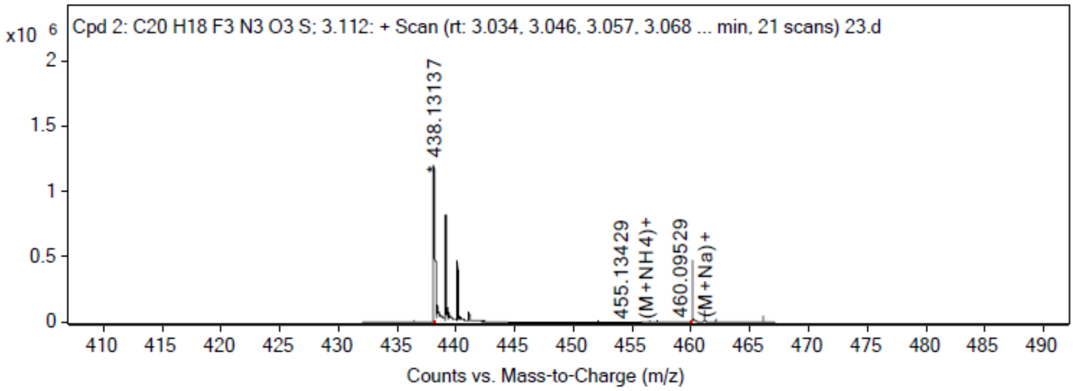


**MS Spectrum Peak List**

| Obs. *m/z* | Charge | Abund | Ion/Isotope | Tgt Mass Error (ppm) |
|---|---|---|---|---|
| 437.09596 | 1 | 614.12 | M+ | 12.78 |
| 438.10908 | 1 | 10678.29 | (M+H)+ | 0.66 |
| 438.13137 | | 1206603.73 | | |
| 439.11323 | 1 | 2842.31 | (M+H)+ | -2.04 |
| 440.10169 | 1 | 1031.5 | (M+H)+ | 17.42 |
| 455.13429 | 1 | 229.03 | (M+NH4)+ | 3.58 |
| 460.09529 | 1 | 11240.28 | (M+Na)+ | -8.63 |
| 461.0982 | 1 | 2619.27 | (M+Na)+ | -8.5 |
| 462.08445 | 1 | 1270.68 | (M+Na)+ | 14.83 |

--- End Of Report ---

Z8526708690

Data File: C:\USERS\PUBLIC\DOCUMENTS\CHEMSTATION\1\DATA\2023-05-12_NIGHT\2023-05-12 2023-05-12 17-42-28\BB338254$1_8199_F1->
Sample Name: BB338254$1_8199_F1

=====================================================================

Acq. Operator : SYSTEM Location: 92
Acq. Instrument : HPLC_07
Injection Date : 6:46:10 PM 5/12/2023
Injection Volume: 5 mkl
Column: Chiralpak IC (250x4.6 mm, 5 mkm)--833VJ002-DA181--7
Mobile Phase: Hexane:MeOH:IPA, 70:15:15
Flow Rate: 0.6 ml/min

=====================================================================

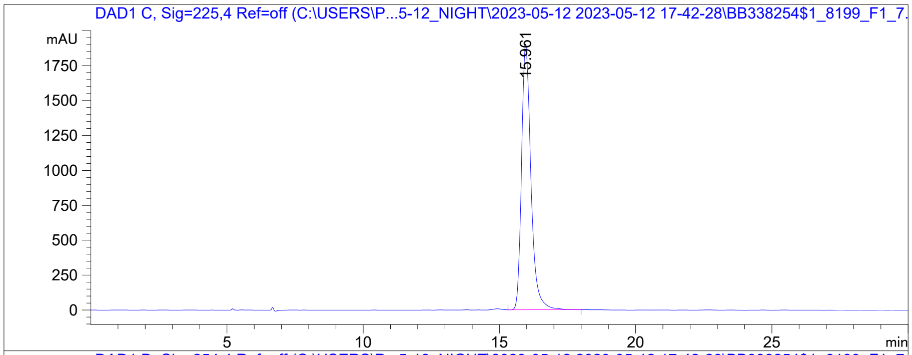


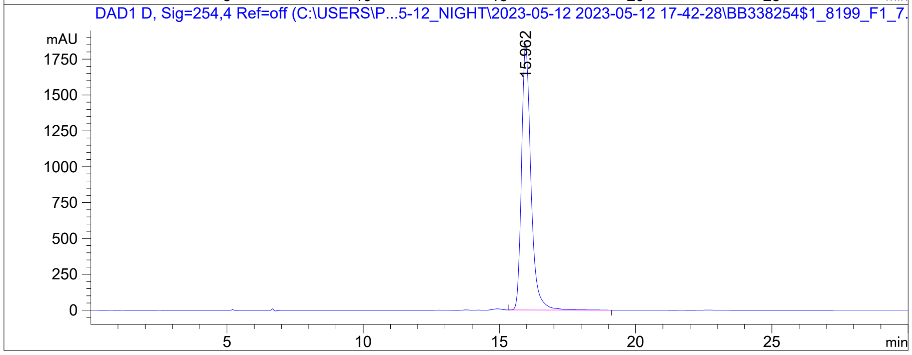


Signal: DAD1 C, Sig=225,4 Ref=off

| RetTime(min) | Area,% | Area | Resolution | Selectivity | 30.001 |
|---|---|---|---|---|---|
| 15.9612 | 100.00 | 45699 | | | |

Signal: DAD1 D, Sig=254,4 Ref=off

| RetTime(min) | Area,% | Area | Resolution | Selectivity | 30.001 |
|---|---|---|---|---|---|
| 15.9622 | 100.00 | 44470 | | | |



Z8526708690

R3946215

MaxPeak: 97.90%
Ret_Time: 3.410 min

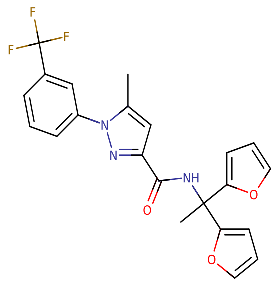

**Mol Wt** 429.39
**Exact Mass** 429.15

| # | Time | Area% |
|---|---|---|
| 1 | 3.410 | 97.90 |
| 2 | 3.526 | 2.10 |

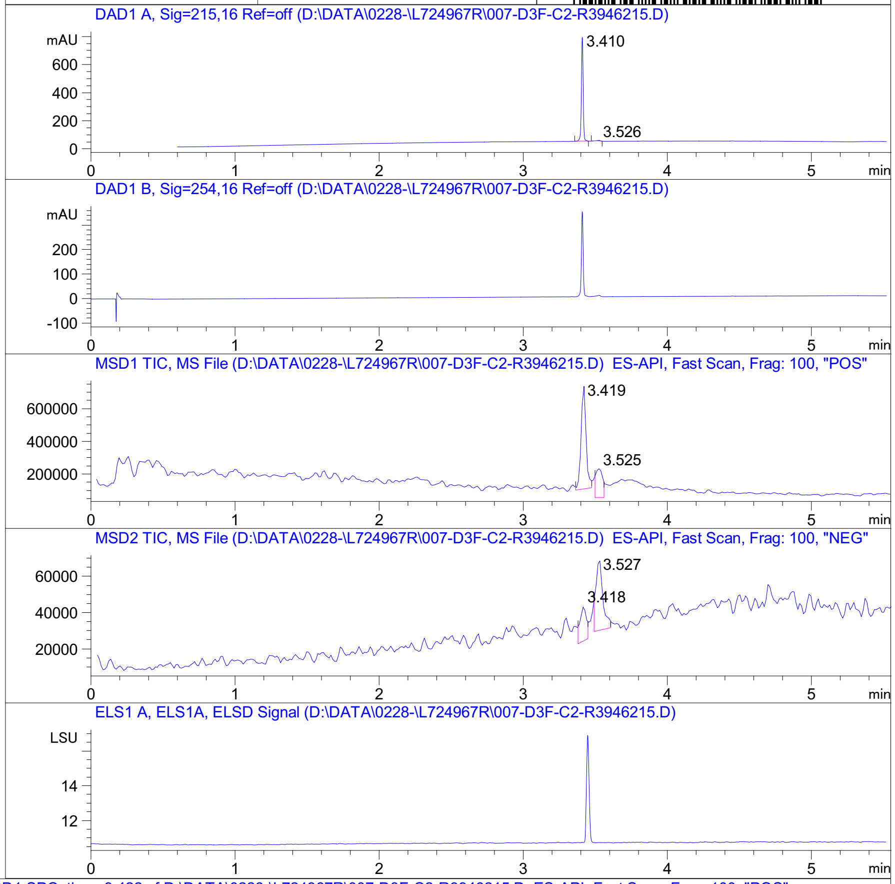


RT 3.419

RT 3.525

RT 3.418

RT 3.527

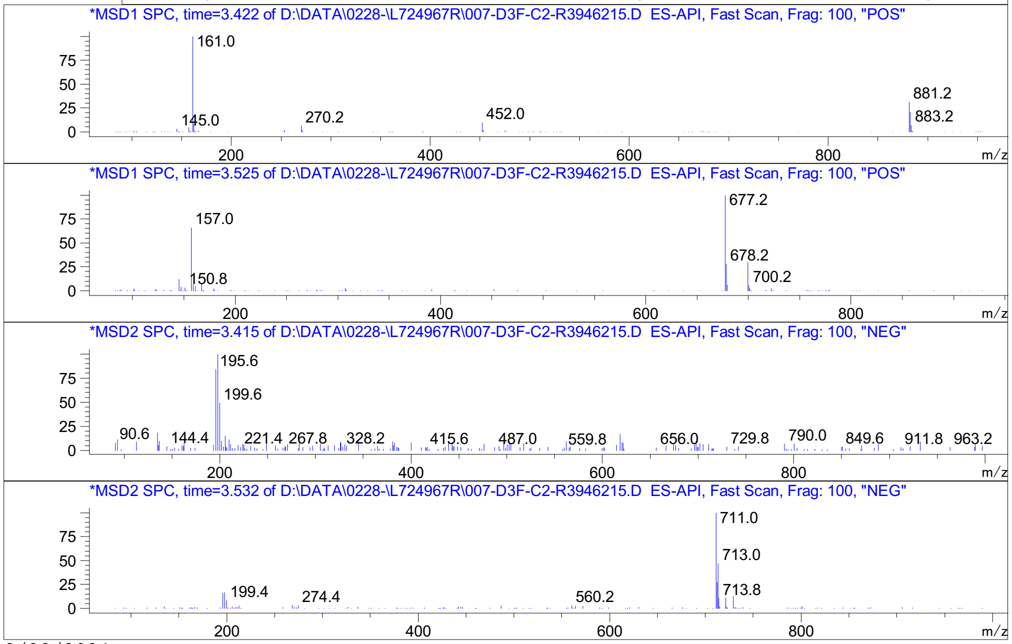


Inj.Date 2/28/2024 VB
C:\Users\Public\Documents\ChemStation\1\Data\02_28\L724967R\UZRX45_3-5V.M

Z8703004936

Z8703004936_1H

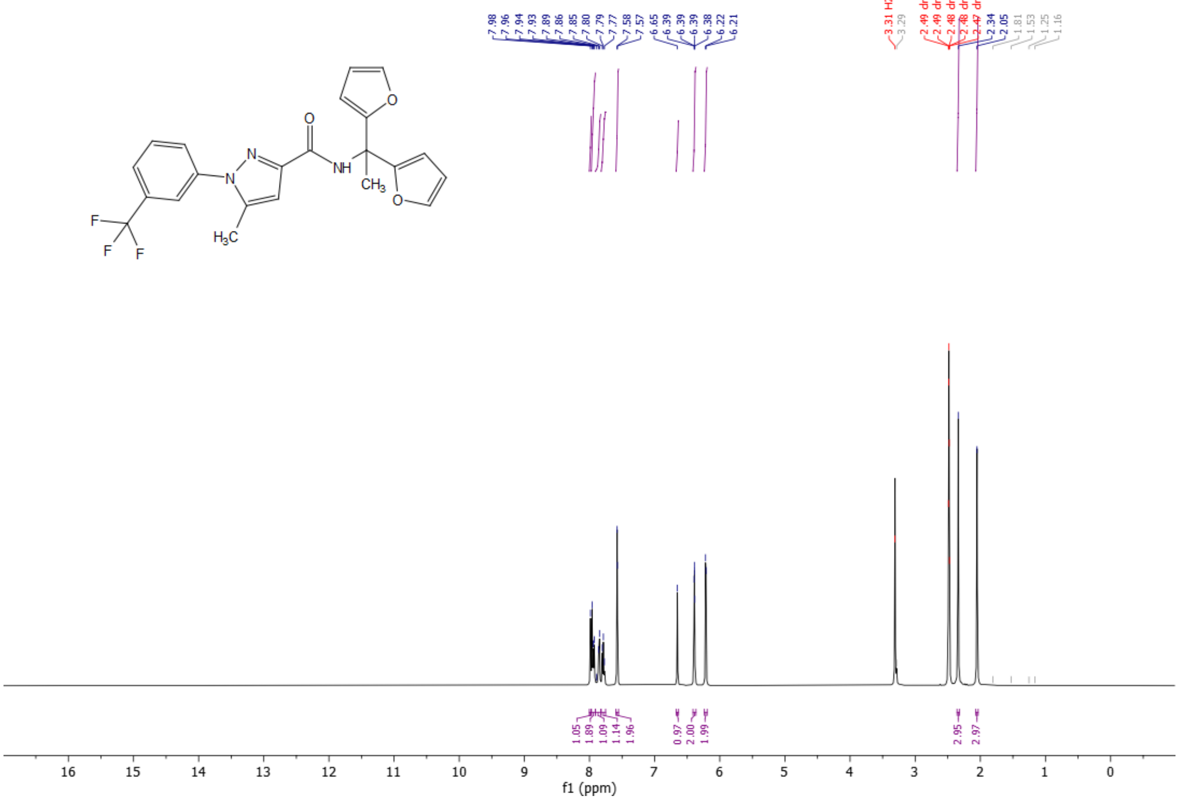


Z8703004936

Z8703004936_13C

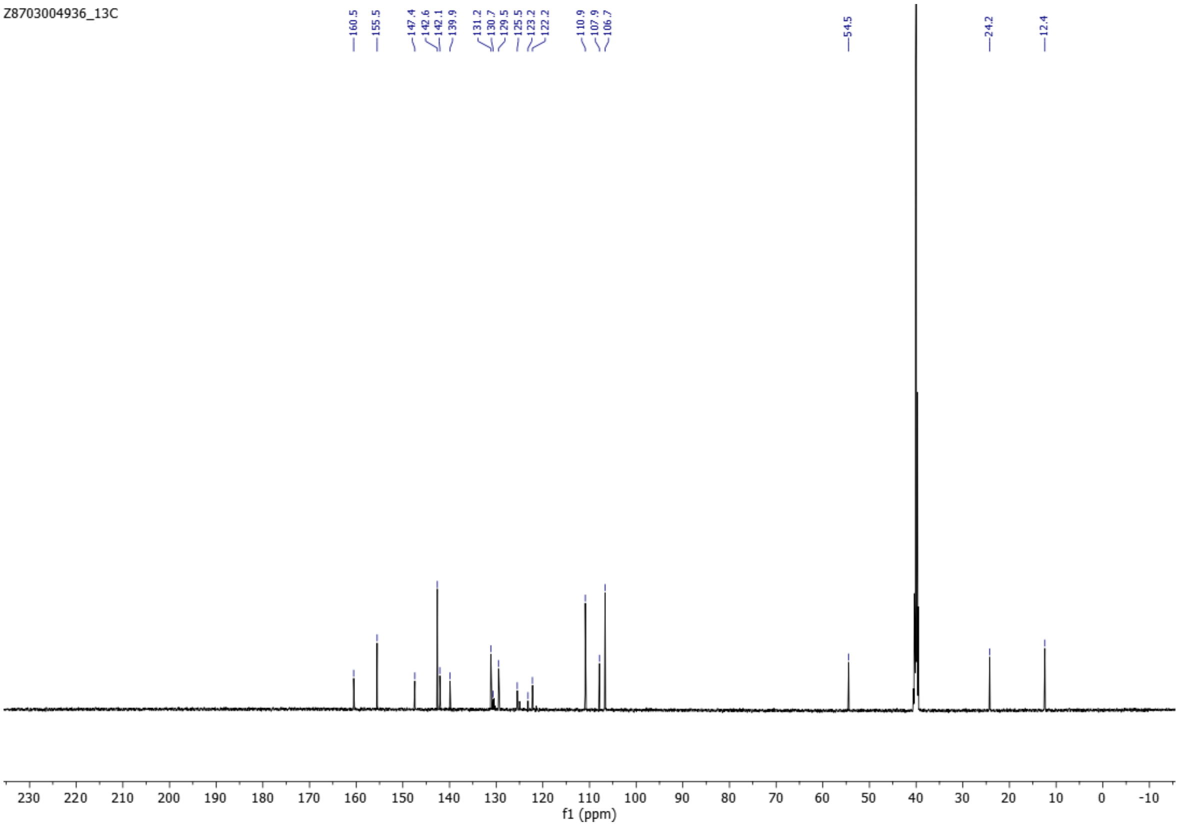


Z8703004936

## Supplementary References

80. Tolmachev, A. *et al.* Expanding Synthesizable Space of Disubstituted 1,2,4-Oxadiazoles. *Acs Comb. Sci.* **18**, 616–624 (2016).
