# Supplementary material for: Virtual library docking for cannabinoid-1 receptor agonists with reduced side effects
Source: Nat Commun. 2025 Mar 6;16:2237. doi: 10.1038/s41467-025-57136-7 (PMC11882969; doi:10.1038/s41467-025-57136-7)
Supplement: Supplementary file 2 — Description of Additional Supplementary Files [file 41467_2025_57136_MOESM2_ESM.docx]

**Description of Additional Supplementary Files**

**Supplementary Data 1:** This file contains a list of all compounds described in this work. For each compound, the general ID, the virtual library code (ZINC / Design ID), the Enamine catalog code (Enamine ID), 2D information (SMILES), molecular weight (MW, Da), purity percentage, and a brief description of the group the molecule was derived from are provided. For a subset of molecules tested in single point radioligand displacement assays, the % displacement (mean and SEM) of radiolabeled 1H-CP55940 is provided.
