# Supplementary material for: Virtual library docking for cannabinoid-1 receptor agonists with reduced side effects
Source: Nat Commun. 2025 Mar 6;16:2237. doi: 10.1038/s41467-025-57136-7 (PMC11882969; doi:10.1038/s41467-025-57136-7)
Supplement: Supplementary file 4 — Reporting Summary [file 41467_2025_57136_MOESM4_ESM.pdf]

nature portfolio

Corresponding author(s): Brian K. Shoichet
Allan I. Basbaum
Alexandros Makriyannis
Kaavya Krishna Kumar

Last updated by author(s): Jan 28, 2025

Reporting Summary

Nature Portfolio wishes to improve the reproducibility of the work that we publish. This form provides structure for consistency and transparency in reporting. For further information on Nature Portfolio policies, see our Editorial Policies and the Editorial Policy Checklist.

Statistics

For all statistical analyses, confirm that the following items are present in the figure legend, table legend, main text, or Methods section.

n/a	Confirmed	
☐	☒	The exact sample size (n) for each experimental group/condition, given as a discrete number and unit of measurement
☐	☒	A statement on whether measurements were taken from distinct samples or whether the same sample was measured repeatedly
☐	☒	The statistical test(s) used AND whether they are one- or two-sided<br>*Only common tests should be described solely by name; describe more complex techniques in the Methods section.*
☒	☐	A description of all covariates tested
☐	☒	A description of any assumptions or corrections, such as tests of normality and adjustment for multiple comparisons
☐	☒	A full description of the statistical parameters including central tendency (e.g. means) or other basic estimates (e.g. regression coefficient) AND variation (e.g. standard deviation) or associated estimates of uncertainty (e.g. confidence intervals)
☐	☒	For null hypothesis testing, the test statistic (e.g. F, t, r) with confidence intervals, effect sizes, degrees of freedom and P value noted<br>*Give P values as exact values whenever suitable.*
☒	☐	For Bayesian analysis, information on the choice of priors and Markov chain Monte Carlo settings
☒	☐	For hierarchical and complex designs, identification of the appropriate level for tests and full reporting of outcomes
☒	☐	Estimates of effect sizes (e.g. Cohen's d, Pearson's r), indicating how they were calculated

Our web collection on statistics for biologists contains articles on many of the points above.

Software and code

Policy information about availability of computer code

Data collection	Docking was done using DOCK3.7.2. CryoEM Data was collected automatically on a Titan Krios (FEI) using SerialEM.
Data analysis	DOCK3.7.2<br>PyMOL v2.5.5<br>CHEMGRID 3.2.1<br>AMBER v.14<br>QNIFFTv.22<br>REDUCE v.2<br>Arthor v.4.2.4<br>Smallworld v.5.6.5<br>CryoSPARC v.4.6.2<br>DOCK6.12<br>CHEMDRAW 21.0.0<br>Microsoft Excel v.16.77.1<br>WinNonlin 5.2<br>CHEMBL24<br>UCSF Chimera 1.15<br>GraphPad Prism 9<br>CSD 2020.0.1

Schrodinger 2019 Maestro v11.9
rdkit 2020.09.1
Analyst 1.6.3
FluorEssence v3.8
MotionCor2 v1.3.0
CTFFIND4
RELION v2.1.0
CryoSparc v4.1.1
UCSF ChimeraX v1.5
Coot v0.9.6
Phenix v1.19.2-4158
FluorEssence v3.8

For manuscripts utilizing custom algorithms or software that are central to the research but not yet described in published literature, software must be made available to editors and reviewers. We strongly encourage code deposition in a community repository (e.g. GitHub). See the Nature Portfolio guidelines for submitting code & software for further information.

Data

Policy information about availability of data

All manuscripts must include a data availability statement. This statement should provide the following information, where applicable:

- Accession codes, unique identifiers, or web links for publicly available datasets
- A description of any restrictions on data availability
- For clinical datasets or third party data, please ensure that the statement adheres to our policy

Additional behavioral and pharmacological data are provided in the Supplementary Information file. Source Data are provided with this paper as Source Data file. The 1350-CB1R (receptor alone) model (Fig. 3) generated in this study has been deposited in the deposited to the Protein Data Bank under accession code 9DGI [https://www.rcsb.org/structure/9DGI] and the map coordinates to the EMDB under accession code EMD-46828 [https://www.ebi.ac.uk/emdb/ EMD-46828]. The composite model was deposited to the Protein Data Bank under accession code 9EGO [https://www.rcsb.org/structure/9EGO] and the composite map to the EMDB under accession code EMD-47992 [https://www.ebi.ac.uk/emdb/ EMD-47992]. The '1350-CB1R-Gi/o model was deposited to the Protein Data Bank under accession code 8GAG [https://www.rcsb.org/structure/8GAG] and the map was deposited to the EMDB under accession code EMD-29898 [https://www.ebi.ac.uk/emdb/ EMD-29898]. The Protein Data Bank entries for the structures used for the docking and model building can be found under accession code 5XR8 [https://www.rcsb.org/structure/5XR8] and 6N4B [https://www.rcsb.org/structure/6N4B]. The docking results, including DOCK scores, smiles, and ZINC IDs for all scored molecules and 3D poses for the top 500,000 molecules as well as input docking grids and optimized structures are available on the LSD website [https://lsd.docking.org/targets/CB1R]. Synthetic methods, chemical identities, purities (LC/MS), yields and spectroscopic analysis (H-NMR) for active compounds are provided in Supplementary Methods. A list of all de novo compounds, their 2D structures, and their synthetic purities can be found in Supplementary Data 1. All compounds may be ordered from Enamine.

Research involving human participants, their data, or biological material

Policy information about studies with human participants or human data. See also policy information about sex, gender (identity/presentation), and sexual orientation and race, ethnicity and racism.

Reporting on sex and gender	NA
Reporting on race, ethnicity, or other socially relevant groupings	NA
Population characteristics	NA
Recruitment	NA
Ethics oversight	NA

Note that full information on the approval of the study protocol must also be provided in the manuscript.

Field-specific reporting

Please select the one below that is the best fit for your research. If you are not sure, read the appropriate sections before making your selection.

☒ Life sciences ☐ Behavioural & social sciences ☐ Ecological, evolutionary & environmental sciences

For a reference copy of the document with all sections, see nature.com/documents/nr-reporting-summary-flat.pdf

Life sciences study design

All studies must disclose on these points even when the disclosure is negative.

Sample size	We did not perform sample-size calculations. We modeled our sample sizes for behavioral studies on previous studies using a similar approach to our own, which have been demonstrated to be capable of detecting significant changes (Scherrer et al, 2009; Muralidharan, A. et

	al 2021).
Data exclusions	No data were excluded.
Replication	All replications were successful at least twice.
Randomization	The animals were randomly assigned to the treatment group and control group. For behavioral experiments, animals were initially placed into one cage and allowed to free run for a few minutes. Next, each animal was randomly picked up, injected with the drug or vehicle control and placed into a separate cylinder before the behavior test. All experiments were for animal behavior and followed this randomization protocol. For in vitro assays, randomization was not considered because no group allocations were made.
Blinding	For all behavioral tests, the experimenter was always blind to treatment. For in vitro tests, randomization is irrelevant and not considered in the study design because these experiments do not require subjective assessment by the experimenter.

Reporting for specific materials, systems and methods

We require information from authors about some types of materials, experimental systems and methods used in many studies. Here, indicate whether each material, system or method listed is relevant to your study. If you are not sure if a list item applies to your research, read the appropriate section before selecting a response.

Materials & experimental systems

n/a	Involved in the study
☐	☒ Antibodies
☐	☒ Eukaryotic cell lines
☒	☐ Palaeontology and archaeology
☐	☒ Animals and other organisms
☒	☐ Clinical data
☒	☐ Dual use research of concern
☒	☐ Plants

Methods

n/a	Involved in the study
☒	☐ ChIP-seq
☒	☐ Flow cytometry
☒	☐ MRI-based neuroimaging

Antibodies

Antibodies used	scFV16 was used to stabilize the CB1-Gi complex. It is described in Maeda et al. Nature Comm, 2018.
Validation	scFv16 was produced in Brian Kobilka's lab and was not used for probing purposes, therefore no validation is required.

Eukaryotic cell lines

Policy information about cell lines and Sex and Gender in Research

Cell line source(s)	1) Sf9, Expression Systems, Cat 94-001S.Tni Cells (Hi-5), Expression Systems, Cat 94011S<br>2) HEK293 clonal cell line (HEK293SL cells) for bioSens-All experiments was derived and characterized in Namkung et al., Nat Commun. 2016 Jul 11;7:12178. Source of original HEK293 cells was American Type Culture Collection (ATCC).<br>3) Rat brains were purchased from Bioivt (Westbury, NY; product code: RAT00BRAINMZN)<br>4) The HEK293 cells expressing hCB1/2R were provided from the Laboratory of Ken Mackie.<br>5) All CHO-K1 cells were purchased from ATCC (CCL-61)<br>6) The TRUPATH HEK293T cells were purchased from ATCC (CRL-3216)<br>Sex of the cells was not considered in the study designs.<br>7) The HTLA cells used in the Tango assay (a HEK293 cell line stably expressing a tTA-dependent luciferase reporter and a β-arrestin2-TEV fusion gene) were a gift from the laboratory of R. Axel.<br>8) Cultured canine MDR1 Knockout, Human MDR1 Knockin MDCKII (MDR1-MDCKII) cells were purchased from Sigma-Aldrich Cat#MTOX1303.<br>8) hCB1 CHO cells for Lance assay were provided by Dr. Laura Bohn's lab.

Authentication	1) Sf9 cell lines are maintained by the supplier. No additional authentication was performed by the authors of this study.<br>2) HEK293 cell lines are maintained by the supplier. No additional authentication was performed by the authors of this study.<br>3) Rat brains are are maintained by the supplier. No additional authentication was performed by the authors of this study.<br>4) No authentication of the HEK293 cells for binding studies was performed by the authors of this study.<br>5) CHO-K1 cell lines are maintained by the supplier. No additional authentication was performed by the authors of this study.<br>6) HEK293T cell lines are maintained by the supplier. No additional authentication was performed by the authors of this study.<br>7) HTLA cells were not authenticated by the authors of this paper.<br>8) MDR1-MDCKII cells are maintained by the supplier. No additional authentication was performed by the authors of this study.<br>9) The cells were not authenticated for this study.
Mycoplasma contamination	1) Cell lines are tested by manufacturer for contamination, but not were not further tested by the authors of this study.<br>2) Cells were tested for mycoplasma contamination on a regular basis. Cells were free of contaminations.<br>3) Rat brains were not tested for mycoplasma contamination.<br>4) Cells were tested for mycoplasma contamination on a regular basis. Cells were free of contaminations.<br>5) Cells were tested for mycoplasma contamination on a regular basis. Cells were free of contaminations.<br>6)Cells were tested for mycoplasma contamination on a regular basis. Cells were free of contaminations.<br>7) Cells were tested for mycoplasma contamination on a regular basis. Cells were free of contaminations.<br>8) Cells were tested for mycoplasma contamination on a regular basis. Cells were free of contaminations.<br>9) The cells were not evaluated for mycoplasma contamination.
Commonly misidentified lines<br>(See ICLAC register)	No commonly misidentified lines were used in this study.

Animals and other research organisms

Policy information about studies involving animals; ARRIVE guidelines recommended for reporting animal research, and Sex and Gender in Research

Laboratory animals	Adult (8-10 weeks old) male C56BL/6 (strain # 664), CB1R knockout (strain #36108), and CB2R knockout (strain #5786) mice were purchased from the Jackson Laboratory. The housing conditions used a 12 hour light on-light off schedule. 22°C and relative humidity of 58%.
Wild animals	No wild animals were used in this study.
Reporting on sex	All behavioral experiments were performed with male mice. Sex was not considered in the study design and is not considered to significantly change study outcomes
Field-collected samples	No field collected samples were used in this study
Ethics oversight	All behavioral animal experiments were approved by the Institutional Animal Care and Use Committee at UCSF (protocol #AN195657) and were conducted in accordance with the NIH Guide for the Care and Use of Laboratory animals. Pharmacokinetic experiments were performed by Bienta (Enamine Biology Services) in accordance with Enamine pharmacokinetic study protocols and Institutional Animal Care and Use Guidelines (protocol number 1-22/2020).

Note that full information on the approval of the study protocol must also be provided in the manuscript.

Plants

Seed stocks	NA
Novel plant genotypes	NA
Authentication	NA
